# Comprehensive Identification and Annotation of Cell Type-Specific and Ubiquitous CTCF-Binding Sites in the Human Genome

**DOI:** 10.1371/journal.pone.0041374

**Published:** 2012-07-19

**Authors:** Hebing Chen, Yao Tian, Wenjie Shu, Xiaochen Bo, Shengqi Wang

**Affiliations:** Department of Biotechnology, Beijing Institute of Radiation Medicine, Beijing, China; Université Paris-Diderot, France

## Abstract

Chromatin insulators are DNA elements that regulate the level of gene expression either by preventing gene silencing through the maintenance of heterochromatin boundaries or by preventing gene activation by blocking interactions between enhancers and promoters. CCCTC-binding factor (CTCF), a ubiquitously expressed 11-zinc-finger DNA-binding protein, is the only protein implicated in the establishment of insulators in vertebrates. While CTCF has been implicated in diverse regulatory functions, CTCF has only been studied in a limited number of cell types across human genome. Thus, it is not clear whether the identified cell type-specific differences in CTCF-binding sites are functionally significant. Here, we identify and characterize cell type-specific and ubiquitous CTCF-binding sites in the human genome across 38 cell types designated by the Encyclopedia of DNA Elements (ENCODE) consortium. These cell type-specific and ubiquitous CTCF-binding sites show uniquely versatile transcriptional functions and characteristic chromatin features. In addition, we confirm the insulator barrier function of CTCF-binding and explore the novel function of CTCF in DNA replication. These results represent a critical step toward the comprehensive and systematic understanding of CTCF-dependent insulators and their versatile roles in the human genome.

## Introduction

Chromatin insulators are small segments of DNA that have an integral role in gene regulation through contributions to the formation and maintenance of active or inactive transcription programs. Insulators can prevent gene silencing by inhibiting heterochromatin spread and can prevent transcriptional enhancers from activating unrelated promoters. Insulators were originally identified in *Drosophila*, and six insulator-binding proteins that mediated insulator activity were subsequently identified [1]–[7]. However, CTCF (CCCTC-binding factor) remains the only protein implicated in the establishment of insulators in vertebrates, so far [1], [8]–[10].

In addition to binding chromatin insulators, CTCF, an evolutionarily conserved and ubiquitously expressed 11-zinc-finger DNA-binding protein [11], [12], has critical roles in transcriptional regulation [13]–[15]. CTCF was discovered as a negative regulator of the *MYC* oncogenes in chicken, mouse, and human [16]–[18], although this function has been challenged recently [19], [20]. Later, CTCF was found to be involved in several transcriptional mechanisms such as gene activation [21], [22] and enhancer blocking [8], [17], [23]–[30]. The insulator function of CTCF has also been implicated in imprinting at the Igf2/H19 locus [23], [29], [31]–[33] and in X chromosome inactivation and the escape from X-linked inactivation [34]–[36].

Many recent studies have been devoted to the identification and characterization of CTCF-binding sites in the human genome. A computational analysis of the human conserved noncoding elements identified nearly 15,000 potential CTCF-binding sites [37]. By employing chromatin immunoprecipitation in combination with microarray hybridization (ChIP-chip), Ren and colleagues reported 13,804 CTCF-binding sites in IMR90 human fibroblasts [38]. In further studies with IMR90 and U937 cells, this group also found that CTCF-binding site localization is largely invariant across different cell types [38]. In an independent study, Zhao and colleagues used ChIP in combination with high-throughput sequencing (ChIP-Seq) to identify 20,262 CTCF target sites in resting human CD4+ T cells [39]. Upon reanalysis with a new algorithm that enabled detection of binding events with enhanced sensitivity and specificity, the number of binding sites was increased to 26,814 [40]. Most recently, ChIP-Seq analyses revealed 19,308, and 19,572 CTCF-binding sites in HeLa and Jurkat cells, respectively [41]. Significant binding of CTCF was detected at the boundaries of repressive chromatin domains marked by H3K27me3, and the association of CTCF with the domain boundaries was found to be cell type-specific [41].

While these studies provide critical information regarding the insulator function of CTCF binding, the CTCF-binding sites were investigated in only a few human cell types. Thus, it is unclear whether the observed cell type-specific differences in CTCF-binding sites are functionally significant. In order to thoroughly investigate CTCF-binding sites across human cells and determine the differences in CTCF-mediated functions between cell types, it is important to examine CTCF across many more human cell types.

In this study we identified and characterized cell type-specific and ubiquitous CTCF-binding sites in the human genome across 38 human cell lines, covered cell types designated by the Encyclopedia of DNA Elements (ENCODE) consortium [42]–[44]. Collectively, our results provide a more comprehensive and systematic resource for understanding the role of cell type-specific and ubiquitous CTCF-binding sites in chromatin insulation, gene regulation, chromatin organization, and DNA replication in human cells.

## Results

### Comprehensive genome-wide mapping of CTCF-binding sites

#### Classification of CTCF-binding sites

Approximately 66,800 CTCF-binding sites were identified from each cell type (Table S1). Lineage analysis revealed that the closest clustering of CTCF-binding sites occurred with sites from cell lines derived from common progenitors (Figure S1). Indeed, while the overlap of CTCF-binding sites between most pairs of cell lines (694 out of 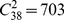) was more than 50%, the highest overlap (79.24%) was found between the two lymphocyte cell lines (GM12875 and GM12873), and the lowest overlap (25.99%) was found between GM12801 and HepG2 cells (Figure S2).

**Figure 1:**
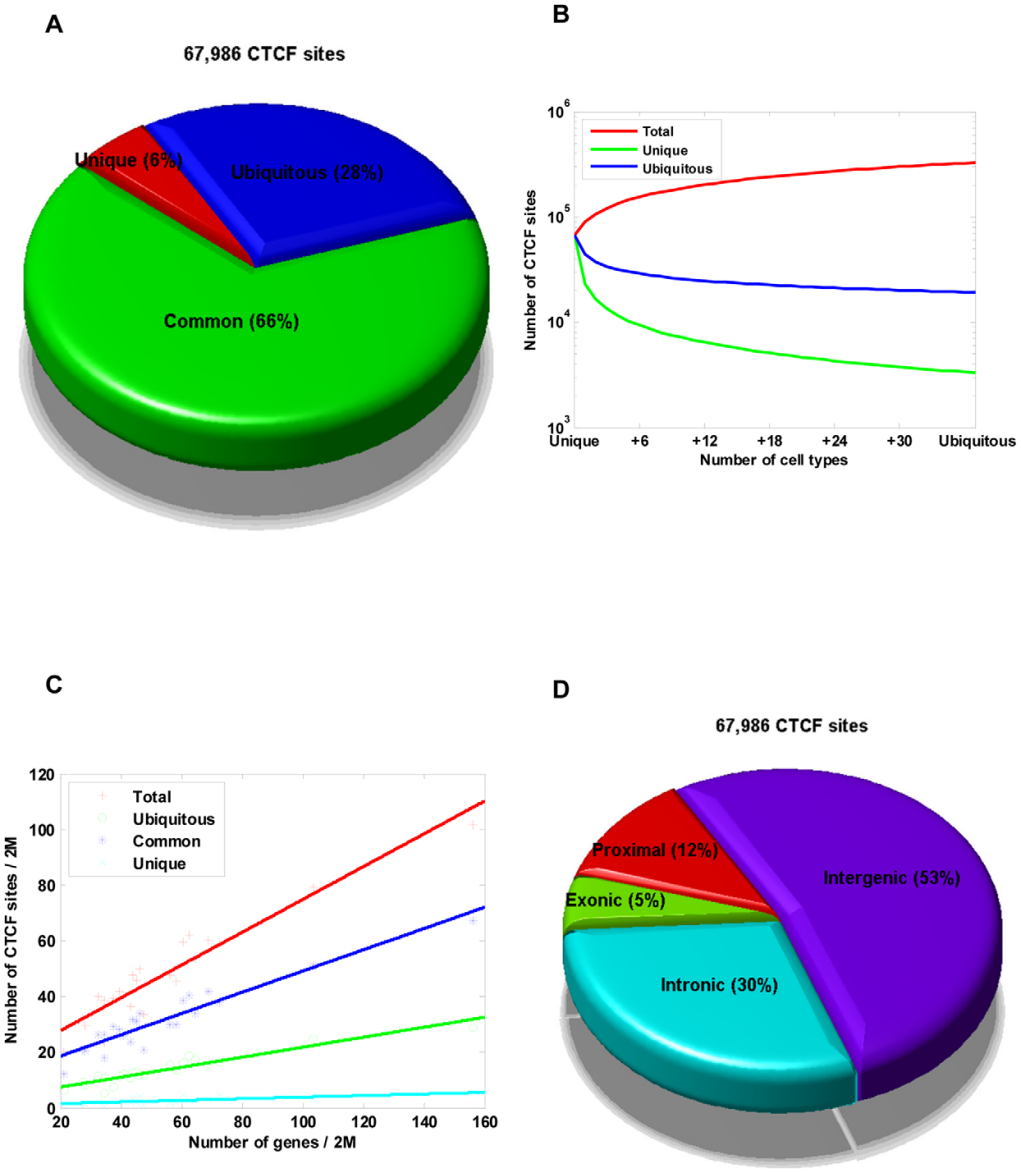
Identification and Characterization of CTCF-binding sites across 38 cell types. (A) Genome-wide distribution of CTCF-binding sites relative to cell type. Total number of CTCF-binding sites in the K562 cell is shown. The proportions of cell type-specific, common, and ubiquitous CTCF sites are indicated. (B) Genome-wide saturation analysis of CTCF-binding sites across 38 cell types. Cumulative number of cell types covered by CTCF-binding sites from increasing numbers of cell lines (*x*-axis). Cumulative number covered by all (red), cell type-specific (green), and ubiquitous (blue) CTCF-binding sites from any cell line. Each point represents an averaged value of all possible cell line combinations. (C) Line graph depicting the number of each type of CTCF-binding site and the genes on each chromosome. The points plotted on the *x*-axis represent the number of genes per 2 Mbp, and points on the *y*-axes represent the number of CTCF-binding sites per 2 Mbp. (D) Chart presenting the genome-wide distribution of CTCF-binding sites in proximal promoters (defined as 1 kb upstream and downstream of TSSs) (red), exonic regions (green), intrinsic regions (cyan), and intergenic regions (purple) of K562 cells. The total number of CTCF-binding sites in K562 cell was 67,986.

Considering the lineage-specificity observed with CTCF-binding sites (Figure S1 and S2), we classified CTCF-binding sites as cell type-specific (only found in 1 out of 38 cell lines), common (found in 2–37 cell lines), or ubiquitous (found in all 38 cell lines) (Table S1). In the erythroleukemia cell type K562, 6% of the CTCF-binding sites were cell type-specific, 66% were common, and 28% were ubiquitous (Figure 1A). In addition, the strongest scoring CTCF-binding sites (top 20%) were more likely to be ubiquitous, while the weakest scoring CTCF-binding sites (bottom 20%) were more likely to be cell type-specific (Figure S3). Results from each cell line were similar (Table S2).

#### Saturation of CTCF-binding sites

To determine whether the majority of CTCF-binding sites in the human genome were represented in the datasets under examination, we computed the cumulative number of CTCF-binding sites with respect to the number of cell lines tested, as described in a previous study [45]. Therefore, as additional cell lines were included in the analysis, the total number of CTCF-binding sites being investigated increased. In total, we identified ∼326,840 CTCF-binding sites from the 38 cell lines (Figure 1B), of which ∼19,200 were ubiquitous and ∼126,200 were cell type-specific. However, even after addition of the 38th cell line, we were unable to reach a significant saturation level of total CTCF-binding sites, which would have been represented by equal levels of cell type-specific, common, and ubiquitous CTCF-binding sites.

#### CTCF binding-site and gene densities

To explore the relationship between CTCF-binding sites and genes, we first counted the numbers of CTCF-binding sites and genes within each chromosome (Figure 1C). In general, the CTCF-binding sites strongly correlated with genes in all cell lines examined (in K562, *R* ^2^ = 0.8967, *p* = 8.00E-12; Figure 1C and Table S4A). Additionally, the correlation between ubiquitous CTCF-binding sites and genes was stronger than the correlation between cell type-specific CTCF-binding sites and genes (in K562, 0.8830 vs. 0.4560; Figure 1C). An independent analysis of CTCF localization along each chromosome also confirmed the above findings (Table S4B). Interestingly, CTCF-binding sites correlated highly with strong enhancers and active promoters in all cell lines examined (in K562, *R* ^2^ = 0.9525 and 0.8229, respectively; Table S4C). This is consistent with the role of CTCF as enhancer blocking insulators, which can function by directly sequestering an enhancer, or by directly interacting with a promoter [46].

#### Location of CTCF-binding sites

We next explored the overall CTCF-binding site distribution pattern relative to genes based on the GENCODE annotation published in the University of California, Santa Cruz (UCSC) Genome Browser. In the K562 cell line, 53% of the CTCF-binding sites mapped within intergenic regions (Figure 1D), while only ∼12% of the CTCF-binding sites were located in the proximal promoters. Unexpectedly, a significant number of CTCF-binding sites fell within genes, with 30% in the introns and 5% in the exons (Figure 1D). Similar distribution patterns were observed in other cell lines (Table S1). Interestingly, ubiquitous CTCF-binding sites were located predominately within intergenic regions, whereas cell type-specific CTCF-binding sites were located predominately in the introns (Table S1). No differences in the location of ubiquitous and cell type-specific CTCF-binding sites within proximal promoters and exons were observed (Table S1).

#### Clustering of CTCF-binding sites

To explore how CTCF-binding sites cluster with each other, we examined the distance between adjacent CTCF-binding sites in multiple human cell types. The distances between adjacent CTCF-binding sites were significantly smaller than the distances between adjacent shuffled CTCF-binding sites (16 kb vs. 29 kb, *p* = 0.0000, two-sided Wilcoxon rank sum test), indicating clustering of CTCF-binding sites. Based on the distance distribution, we defined and identified the clusters of CTCF-binding sites. In K562 cells, while as many as 39% (26,808 out of 67,986) of the distinct CTCF-binding sites were located in the genome discretely (classified as CTCF singletons), the majority (61%; 41,178 out of 67,986) of the distinct CTCF-binding sites clustered with others and were grouped into 14,500 CTCF-clusters (Table 1).

**Table 1:** CTCF-cluster mapping and statistic estimation in K562 cells.

CTCF-cluster Mapping and Statistic Estimation								
			CTCF clusters					
	Total peaks	CTCF Singletons	CTCF-2	CTCF-3	CTCF-4	CTCF-5	CTCF-6	CTCF-6+
CTCF-cluster	67986	26808	8368	3236	1458	696	354	388
Shuffled CTCF-cluster	67986	41949±149	8707±76	1943±40	466±20	121±10	33±5	15±4
Distinct CTCFs	67986	26808	16736	9708	5832	3480	2124	3298
Shuffled CTCFs	67986	41949±149	17414±152	5830±121	1867±82	606±53	202±34	114±32
Random probability (%)		1	1	<0.00001	<0.00001	<0.00001	<0.00001	<0.00001

Composition of CTCF-cluster								
			CTCF clusters					
	Total peaks	CTCF Singletons	CTCF-2	CTCF-3	CTCF-4	CTCF-5	CTCF-6	CTCF-6+
Unique	3765	1054	1027	648	400	250	164	222
Common	44984	18264	10883	6251	3741	2225	1363	2257
Ubiquitous	19237	7490	4826	2809	1691	1005	597	819

The random probability of CTCF overlapping was calculated based on the more than 10,000 simulated numbers versus the observed numbers in each category of CTCF-clusters. Genomic DNA segments of the same number and size as the CTCF-defined loci were randomly extracted from the human genome assembly (hg19) as background.

To determine whether the identified CTCF-clusters were due to random chance, we performed a Monte Carlo simulation. We estimated that of the CTCF-clusters (hereafter referred to as CTCF-2 for CTCF-clusters with two overlapping members, CTCF-3 for clusters with three overlapping members, and so forth) 100% of the CTCF-2 clusters and <0.0001% of the CTCF-3 clusters could result from random sampling (Table 1). This result was consistent across the human cell types examined (Table S2).

### Evolutionary and functional features of CTCF-binding sites

#### Conservation analysis of CTCF-binding sites

**Figure 2:**
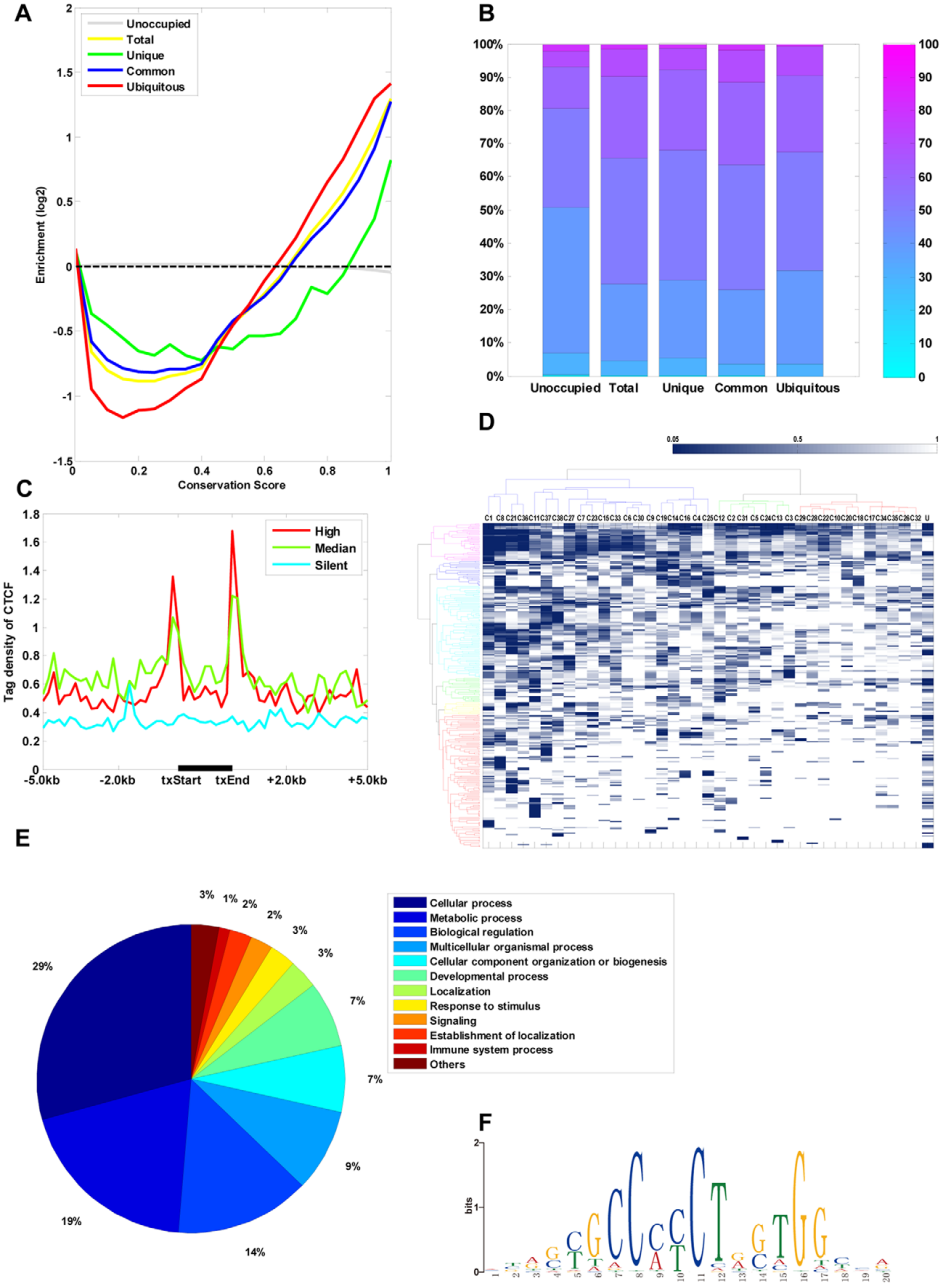
Evolutionary and functional features of CTCF-binding sites. (A) Conservation profiles for each type of CTCF-binding site in 38 cell types. The *x*-axis indicates the PhastCons score of bases covered by the binding sites ranging from 0 (no conservation) to 1.0 (perfect conservation). The *y*-axis represents the log ratio between the number of bases with the given score covered by different types of CTCF-binding sites relative to what would be expected by random site placement and the number of bases with the given score covered by the human genome relative to what would be expected by random site placement. The categories are: Unoccupied, unoccupied CTCF-binding sites that were used as control; Total, all CTCF-binding sites across the 38 cell types; Unique, cell type-specific CTCF-binding sites across the 38 cell types; Common, common CTCF-binding sites across the 38 cell types; and Ubiquitous, ubiquitous CTCF-binding sites across 38 cell types. (B) Distribution of GC content within each type of CTCF-binding site across the 38 cell types. The *y*-axis represents the percentage of CTCF–binding sites with GC content of different ranges (bar on right). The categories are the same as indicated in (A). (C) Normalized tag density of CTCF-binding sites of the most active, median, or most silent genes (*n* = 2,000 per group) across the gene bodies. The plots extend 5 kb 5′ and 3′ of the genic regions. RNA expression was determined in gene bodies for each cell type and exons displaying significantly high or low expression levels relative to the median expression for all cell types were identified. txStart, transcription start site; txEnd, transcription end. (D) GO analysis of cell type-specific and ubiquitous proximal CTCF-binding sites. Clustering of 38 cell types based on common GO nodes. Hierarchical clustering of both the cell types and the common GO nodes was performed based on the calculated EASE scores using the software Cluster 3.0 with average linkage. The relationship between the color intensity and EASE score is illustrated by the color bar. Gray indicates that an EASE score was not calculated for that GO node. The cell type is denoted by the letter and number combination at the top of every column. C1–C38  =  CTCF-binding sites of the 38 cell types (see Figure S1 for details), U  =  ubiquitous CTCF-binding site. (E) Summary of the biological processes regulated by genes related to the cell type-specific and ubiquitous proximal CTCF-binding sites. Annotations were obtained from the Gene Ontology database. (F) Significantly enriched CTCF consensus motifs within ubiquitous CTCF-binding sites graphically depicted using WebLogo.

To explore the evolutionary features of CTCF-binding sites, we first examined the sequence conservation scores for each type of CTCF-binding site. We extracted the phastCons and phyloP cross-species conservation scores based on 46 mammalian species, for each CTCF-binding site. For the various conservation analyses, we used unoccupied CTCF-binding sites (unoccupied sites) in the genome as a negative control. As shown in Figure 2A, CTCF-binding sites across the 38 cell types were substantially more conserved than unoccupied sites (*p* = 0.0000, two-sided Wilcoxon rank sum test). Ubiquitous CTCF-binding sites were more conserved than common CTCF-binding sites (*p* = 0.0002, two-sided Wilcoxon rank sum test), which were more conserved than cell type-specific CTCF-binding sites (*p* = 1.2E–12, two-sided Wilcoxon rank sum test). The results were consistent for both phastCons and phyloP scores for vertebrate, primate, and placental genomes (Table S3).

#### Analysis of CTCF-binding site GC content

We next investigated whether the percentage of guanine (G) and cytosine (C) differed among different types of CTCF binding-sites, since high GC content is typically associated with gene-rich areas and has some functional relevance (Table S3). Each type of CTCF-binding site contained a higher percentage of GC content than the unoccupied sites (Figure 2B, *p* = 0.0000, two-sided Wilcoxon rank sum test). In addition, ubiquitous CTCF-binding sites tended to have much higher GC content than the cell type-specific CTCF-binding sites (Figure 2B, *p* = 0.0000, two-sided Wilcoxon rank sum test). Analysis of the overlap between CpG islands and CTCF-binding sites also indicated that CpG islands are more likely to coincide with ubiquitous CTCF-binding sites than with cell type-specific CTCF-binding sites (9.75% vs. 4.68%, *p* = 3.8851E–004).

#### Analysis of the correlation of CTCF-binding sites and gene expression

We examined the general distribution pattern of CTCF-binding sites near transcription start sites (TSSs) of genes across cell types (Figure 2C) and generated composite tag density profiles of the most active, median, and least active genes (*n* = 2,000 each). It was notable that the CTCF signal peaked near both the 5′ and 3′ ends. In addition, the CTCF profile of active genes was consistently much higher than that of silent genes. Scatter correlation analysis of CTCF-binding sites and gene expression indicated that, although different levels of positive correlation existed across cell types, CTCF signals correlated positively with gene expression (on average, *R* = 0.6244, *p* = 1.20E–5; Figure S4 and Table S5).

#### GO (Gene Ontology) analysis of CTCF-binding sites

To determine whether consistent biological themes could be identified among cell type-specific CTCF-binding sites, we identified enriched GO categories using EASE [47] (EASE score <0.05) based on the genes related to proximal CTCF-binding sites (<1 kb from TSS) that are cell type-specific and ubiquitous across cell types. We identified 136 significant GO nodes that clustered into three main branches across cell type-specific and ubiquitous CTCF-binding site combinations in the 38 cell lines (Figure 2D and Table S6). We found many biological processes consistently regulated by CTCF across the 38 cell types (Figure 2E). Of the assigned regulatory functions, cellular processes (such as cell cycle and cell recognition), metabolic processes (such as cellular and molecular metabolism), and biological regulation (including regulation of zinc ion transmembrane transport) are the most highly represented (Figure 2E). Consistent result was obtained by conducting this analysis based on the genes related to proximal CTCF-binding sites (<1 kb from TSS) across 38 cell types (Figure S5). This result indicated that CTCF is a multifunctional protein involved in gene regulation [46].

#### Analysis of CTCF-binding site motifs

We used the *de novo* motif finder MEME [48] to identify the first five over-represented consensus motifs within ubiquitous CTCF-binding sites and within cell type-specific CTCF-binding sites across the 38 cell types (Table S7). The most over-represented motif in the ubiquitous CTCF-binding sites (Figure 2F) was identical to the canonical CTCF DNA-binding motif identified in previous studies [37], [38], [41], [45]. Although most motifs within cell type-specific CTCF-binding sites were different from the canonical CTCF motif, we still found the canonical motif in 7 out of 38 cell lines. This is consistent with the results of the MAST analysis, in which we found that, on average, the CTCF canonical motif represents 27% of the cell type-specific CTCF-binding sites and 92% of the ubiquitous CTCF-binding sites after accounting for motifs expected to occur by chance (Table S8).

We then compared the identified motifs with known motifs of transcription factor (TF)-binding sites using TOMTOM [49], and found significant similarities to motifs from JASPAR [50], TRANSFAC [51] and UniPROBE [52] database. Among the top-ranking factors, we found the consensus motifs of Pitx2 (TAATCCC), PU.1 (AGGAAG), KROX (CCCGCCCCC), MYOD (CACCTG), ADF-1 (CCGCCGCCGCCGC), ZFX (CAGGCCGCG), SP1 and SP4 (CCCCGCCCC), E2A (CACCTG), and EGR (GCCCCCAC). TOMTOM analyses showed that CTCF-binding sites were associated with a wide spectrum of *cis*-regulatory elements.

To detect associations between TF-binding motifs and GO terms, the MEME tool GOMO [53], [54] was used to assign functional roles to cell type-specific and ubiquitous CTCF-binding motifs. The first three over-represented motifs within cell type-specific or ubiquitous CTCF-binding sites across the 38 cell types were analyzed. GOMO analysis revealed 443 significant GO nodes that clustered into three main branches across over-represented motif combinations from cell type-specific and ubiquitous CTCF-binding sites of the 38 cell lines (Figure S6A and Table S9). The most significant biological processes regulated by these over-represented motifs are consistent with the results of the above GO analysis (Figure 2E and Figure S6B). The result is maintained if we included the first five over-represented motifs within cell type-specific and ubiquitous CTCF-binding sites (Figure S6C–D and Table S9).

### Chromatin features of CTCF-binding sites

#### Nucleosome positioning near CTCF-binding sites

**Figure 3:**
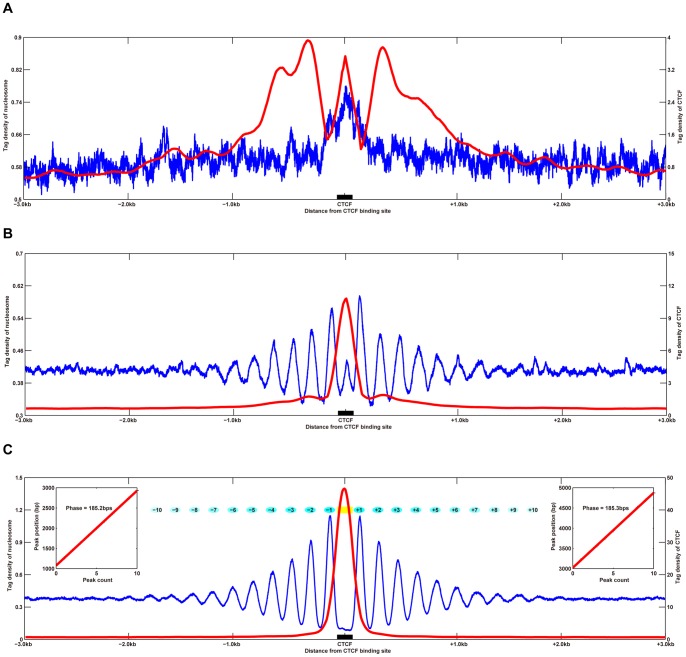
Nucleosome positioning near the CTCF-binding sites in K562 cells. Nucleosome (blue lines) and CTCF-binding sites (red lines) profiles around cell type-specific (A), common (B), and ubiquitous (C) CTCF-binding sites are illustrated. Distances from the CTCF-binding sites are plotted along the *x*-axis. Left and right *y*-axes represent the normalized tag densities of the nucleosome and CTCF-binding sites, respectively. In (C), cyan ovals depict hypothetical nucleosome positions across the site with color intensities reflecting their positioning strength. The CTCF-binding site is indicated by the yellow rectangle. Left inset, linear fit to the positions of the phase peaks within 3 kb downstream of the CTCF-binding sites (slope  = 185.2 bp; 95% confidence interval (CI)  =  [184.6 bp, 185.7 bp]). Right inset, linear fit to the positions of the phase peaks within 3 kb upstream of CTCF-binding sites (slope  = 185.3 bp; 95% CI  =  [184.2 bp, 186.5 bp]).

To examine the positioning patterns of nucleosomes surrounding CTCF-binding sites, we aligned all CTCF-binding sites identified in K562 and GM12878 cells and plotted the CTCF and nucleosome profiles. We observed a striking repeating phasing pattern of these signals in a 3 kb region surrounding the CTCF-binding sites (Figure 3B and Figure S7), and the CTCF-binding site was located in the center of a linker region. The nucleosome profile of the cell type-specific CTCF-binding sites indicated that a nucleosome was occluding the CTCF-binding site, but no other periodically positioned nucleosomes were present (Figure 3A). The noisier nucleosome peak in Figure 3A, which occludes the cell type-specific CTCF-binding site, suggests overlap of nucleosome positions. In contrast, the ubiquitous CTCF-binding site was located in the center of a linker region, flanked on each side by up to 10 pairs of peaks, indicating 20 well-positioned nucleosomes (Figure 3C). The average center-to-center distance of neighboring nucleosomes was 185 bp (insets in Figure 3C). This is consistent with the results of the scatter correlation analysis, in which modest positive correlations between CTCF-binding sites and nucleosome positioning were detected in cell type-specific CTCF-binding sites, but strong negative correlations were detected with ubiquitous CTCF-binding sites (Figure S8 and Table S10).

#### Open chromatin near CTCF-binding sites

**Figure 4:**
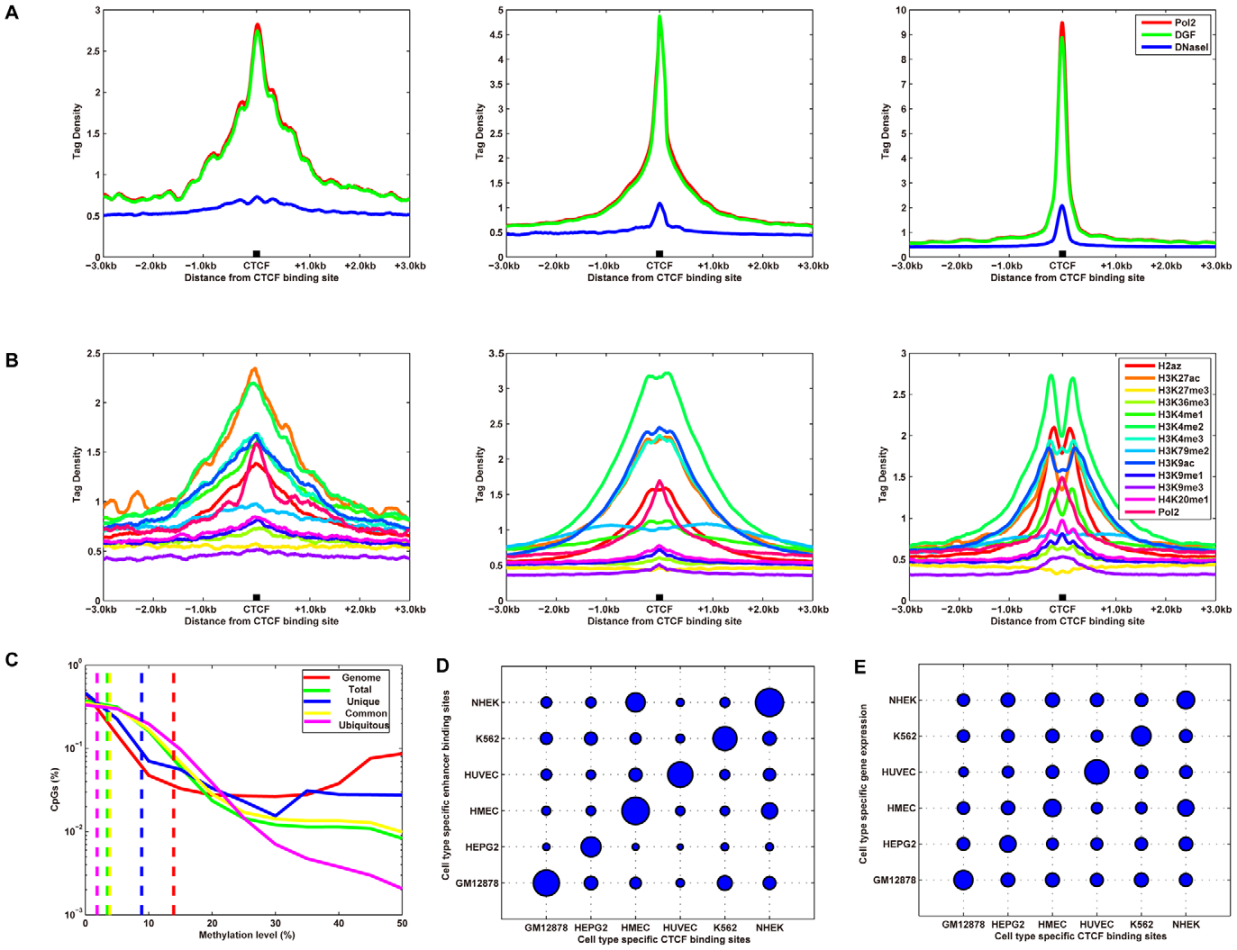
Chromatin features of CTCF-binding sites. (A) Open chromatin proximal to CTCF-binding sites in K562 cells. DNaseI HS, DNaseI DGF, and FAIRE profiles of cell type-specific (left), common (middle), and ubiquitous (right) CTCF-binding sites. The tag density for open chromatin is shown across the CTCF-binding sites and extending 3 kb upstream and downstream of the CTCF-binding sites. (B) Histone modifications proximal to the CTCF-binding sites in K562 cells. Histone modification profiles of cell type-specific (left), common (middle), and ubiquitous (right) CTCF-binding sites. The tag density for modifications is shown across the CTCF-binding sites and extending 3 kb upstream and downstream of the CTCF-binding sites. (C) The smoothed distributions of CpG methylation levels within different types of CTCF-binding sites in K562 cells (for CpGs with ≥10-fold coverage). The distributions of methylation levels (%) across all CpGs identified in all, unique, common, and ubiquitous CTCF-binding sites are illustrated as a smooth approximation of probability density, which was estimated based on a normal kernel function. The *x*-axis represents the density of the methylation levels. The median methylation levels of different types of CTCF sites are illustrated as vertical, dashed lines. (D, E) CTCF-binding sites colocalize with strong enhancers (D) and gene expression (E) in a cell type-specific manner. (D) Cell-type specific CTCF-binding sites (*x*-axis) are mapped relative to cell-specific enhancer binding regions (*y*-axis) in six different cell types. (E) Cell type-specific CTCF-binding sites (*x*-axis) are mapped relative to transcription start sites of genes with cell type-specific expression (*y*-axis). Bubble size represents the level of enrichment.

To characterize the open chromatin patterns surrounding CTCF-binding sites, we aligned the CTCF-binding sites of each group and compared each with the open chromatin of that region. DNaseI hypersensitive sites (HS), DNaseI Digital Genomic Footprinting (DGF), and Formaldehyde-Assisted Isolation of Regulatory Elements (FAIRE) were used to examine open chromatin. As shown in Figure 4A, open chromatin tag densities were sharply elevated, although to varying degrees, within the different types of CTCF-binding sites. The tag densities of open chromatin (DNaseI HS, DGF, and FAIRE) in ubiquitous CTCF-binding sites were much higher than those in cell type-specific CTCF-binding sites. Scatter correlation analysis also detected modest positive correlations between CTCF and DNaseI HS, DGF, and FAIRE in cell type-specific CTCF-binding sites, and strong positive correlations in ubiquitous CTCF-binding sites (Figure S9 and Table S10).

#### Histone modifications near CTCF-binding sites

To characterize the histone modification patterns at CTCF-binding sites, we aligned the CTCF-binding sites of each group and compared each with the histone modifications of that region. Methylation and acetylation were examined, as distinct forms of each have been associated with activation, repression, or both according to context. As shown in Figure 4B, all marks of histone modifications, with the exception of H3K27me3, were sharply elevated, although to varying degrees, within the different types of CTCF-binding sites. The tag densities of these histone modifications were much higher in ubiquitous CTCF-binding sites than in cell type-specific CTCF-binding sites. These data agree with the scatter correlation analysis. For the cell type-specific CTCF-binding sites, we observed modest positive correlations between CTCF and H4K20me1, H3K9me3, H3K9me1, H3K4me1, H3K36me3, H3K27me3, H2AZ, and weak positive correlations between CTCF and H3K9ac, H3K79me2, H3K4me3, H3K4me2, H3K27ac (Figure S9 and Table S10). In ubiquitous CTCF-binding sites, however, strong positive correlations between CTCF and H4K20me1, H3K9me1, H3K9ac, H3K4me3, H3K4me2, H3K4me1, H2AZ were detected, modest positive correlations with H3K9me3, H3K36me3, H3K27ac were detected, and weak correlations with H3K79me2, H3K27me3 were detected (Figure S9 and Table S10).

#### DNA methylation of CTCF-binding sites

To explore the DNA methylation patterns at CTCF-binding sites, we analyzed genome-wide DNA methylation at CpG sites with a modified version of Reduced Representation Bisulfite Sequencing (RRBS) [55]. The methylation levels of CpG dinucleotides in each cell line display a bimodal distribution (Figure 4C), with most being either ‘largely unmethylated’ (<20% of reads showing methylation) or ‘largely methylated’ (>80% of reads showing methylation). More CTCF-binding sites than shuffled CTCF-binding sites contained CpG dinucleotides (*p*<0.00001). Of these CpG dinucleotides located within CTCF-binding sites, on average, 93.6% were largely unmethylated and 6.4% were methylated (≥20% of reads showing methylation), of which only 1.6% were largely methylated.

Notably, over 19% of CpG dinucleotides within cell type-specific CTCF-binding sites were methylated, and 7.5% were largely methylated. However, of the CpG dinucleotides within ubiquitous CTCF-binding sites, less than 4% were methylated, and only 0.6% were largely methylated. Furthermore, the tag densities of CTCF correlated negatively with DNA methylation levels significantly (Figure S10 and Table S10). Ubiquitous CTCF-binding sites correlated negatively with DNA methylation levels significantly, as well (Figure S10 and Table S10). However, cell type-specific CTCF-binding sites did not show significantly negative correlation with DNA methylation levels (Figure S10 and Table S10). These results were consistent across all the human cell types examined (Table S10).

#### CTCF-binding sites colocalize with DNaseI HS sites, histone modifications, regulatory elements, and gene expression

To determine if CTCF-binding sites might cooperate with enhancers and promoters to regulate cell type-specific gene regulation, we performed colocalization analysis with CTCF-binding sites, DNaseI HS sites, histone-modified regions, enhancers, promoters, and gene expression in 6-by-6 cell line combinations, as previously described [56]. We illustrated the enrichment factor with a bubble chart in Figures 4D–E, Figure S11, and Figure S12. The diagonal-matched cell line enrichment values (>1.00 for all comparisons) were much larger than off-diagonal mismatched cell line values (<1.00 for all comparisons).

### CTCF functions in demarcation of chromatin domains

#### Identification of chromatin domains

**Figure 5:**
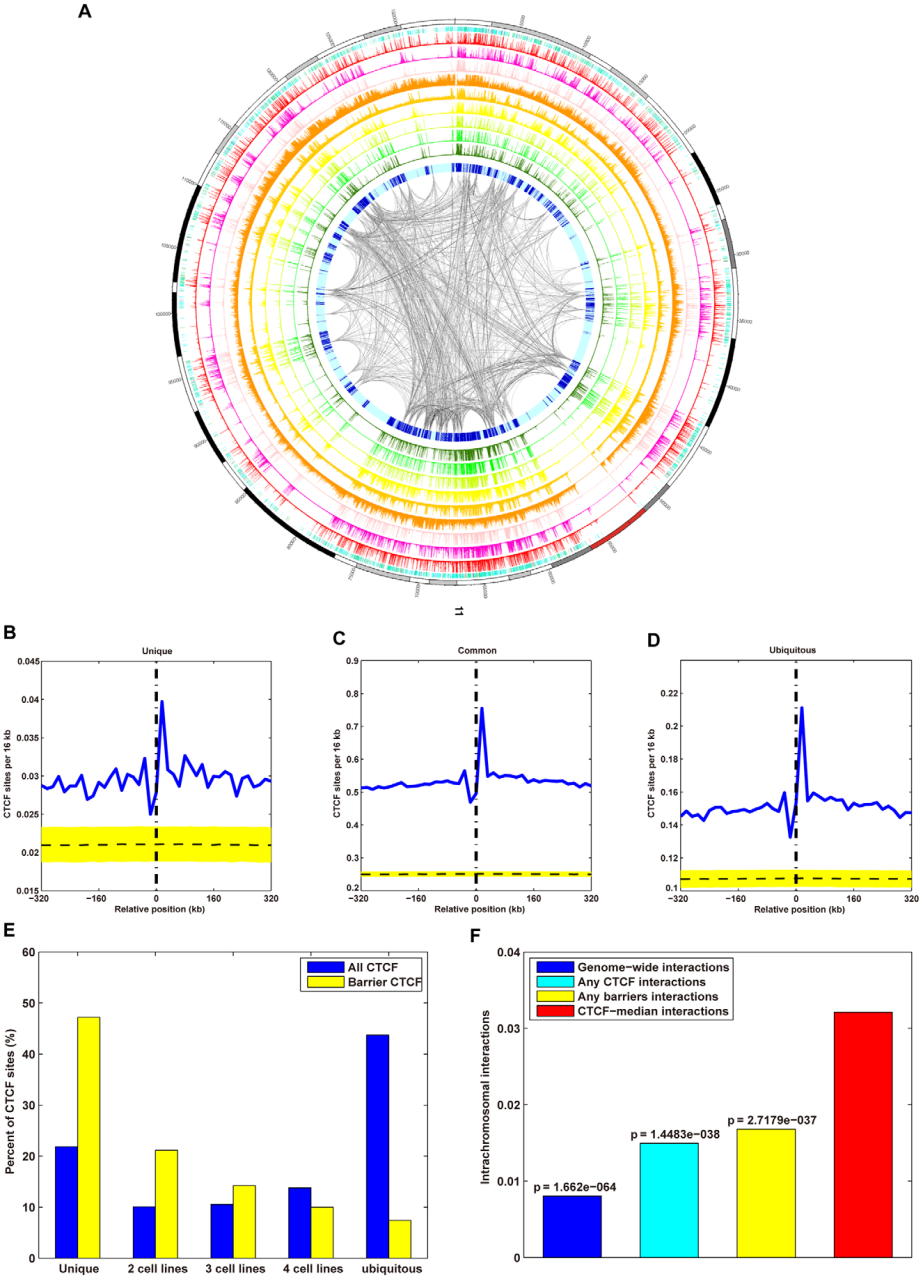
CTCF-binding sites demarcate euchromatin and heterochromatin. (A) Circos map of the whole-genome chromatin domains, associated CTCF-binding sites, DNaseI HS, and histone modifications of chromosome 11 generated using the Circos software package. Chromatin domains were identified in K562 cells using HMMSeg, with DNaseI HS and histone modifications as inputs. The outermost circle (circle 1) represents the chromosome band (scale in kb). Circles 2 and 3 represent the peaks and tag density profile of CTCF-binding sites, respectively. Circle 4 represents the DNaseI HS profile. Circles 5–11 represent the histone modifications H3K27ac, H3K27me3, H3K36me3, H3K4me1, H3K4me2, H3K4me3, and H3K9ac, respectively. Circle 12 represents the euchromatin (medium blue) and heterochromatin (light cyan) domains. Intrachromosomal interactions are drawn in the innermost ring with color intensities (from white to gray) reflecting interaction strength (low to high). (B–D) Number profiles of cell type-specific (B), common (C), and ubiquitous (D) CTCF-binding sites centered on boundaries of different chromatin domains and extended 320 kb upstream of and 320 kb downstream of the boundary at 1 kb resolution. The area to the left of the vertical dash-dot line and all negative coordinates represent heterochromatin domains; the area to the right of the vertical dash-dot line and all positive coordinates represent euchromatin domains. Plotted on the *y*-axis is the normalized number of CTCF-binding sites and on the *x*-axis is distance from the chromatin boundary. Blue lines show moving-window averages with window sizes of 16 kb. The yellow strip represents the region of 5^th^ and 95^th^ percentiles for the number profile of the corresponding 10,000 shuffled CTCF-binding sites. The horizontal dashed line represents the median number profile of the corresponding 10,000 shuffled CTCF-binding sites. (E) Percentage of the cell type-specific, common, and ubiquitous barrier CTCF-binding sites that overlapped with each other within all CTCF and barrier CTCF across five cell types. (F) Chromatin domains are mediated by CTCF loops. Bar chart representing the median intrachromosomal interactions across the human genome (blue bar), and the median intrachromosomal interactions between any CTCF-binding sites (cyan bar), any barrier CTCF-binding sites (yellow bar), and barriers of adjacent chromatin domains (red bar) in K562 cells.

To explore the insulator function of CTCF-binding sites as domain barriers, we identified the heterochromatin and euchromatin domains across the genome using diverse histone modifications and open chromatin as inputs to HMMSeg, a hidden Markov model-based segmentation for parameter learning and region calling [57] (Figure 5A and Figure S13). This analysis revealed, in K562 cells, a total of 14,166 euchromatin domains typically ranged from 9 to 199 kb (5^th^–95^th^ percentiles) with a median length of 31 kb. Of these euchromatin domains, 2,042 were larger than 100 kb and 88 were larger than 500 kb, with the largest domain being 2,979 kb. A total of 14,181 heterochromatin domains that ranged from 5 to 693 kb (5^th^–95^th^ percentiles) with a median length of 27 kb (Table S11) were detected, as well. Of these heterochromatin domains, 3,127 were larger than 100 kb and 959 were larger than 500 kb, with the largest domain being ∼21 Mb (Table S11).

#### CTCF is enriched at the chromatin domain boundaries

The uniquely sharp transitions of transcription status and chromatin composition observed across euchromatin borders indicated that these borders might contain particular elements that separate the euchromatin and heterochromatin types (Figure S14; Materials and Methods). To characterize the CTCF-binding site pattern near the euchromatin domain boundaries and determine if CTCF preferentially marks euchromatin border regions, we aligned all euchromatin domains by their left or right border and calculated average tag density profiles of CTCF across the combined borders (left and mirrored right border regions combined). The average profile of CTCF reflects the abrupt change in signal at these chromatin boundaries (Figure S15A).

#### CTCF-binding at barriers is cell type-specific

To identify the CTCF-bound genomic regions that may act as domain barriers, we searched for CTCF-binding sites that occur near the euchromatin domain boundaries. Based on the enrichment of CTCF-binding sites near domain boundaries (Figure 5B–D), we chose 8 kb as the maximal distance that could exist between the domain boundary and CTCF-binding site for the binding site to be classified as a barrier. We identified, on average, 14,245 (22%) of the CTCF-binding sites across all cell lines as barriers (Table S12). On average, nearly one-half (8,961; 49%) of domain boundaries across cell types, were associated with these barrier CTCF-binding sites. Through comparison with results obtained with shuffled CTCF-binding sites, we determined that the probability of this many CTCF-binding sites colocalizing with the domain boundaries by chance is very low (*p*<1.00E–5 for all cells; Materials and Methods). The total number of CTCF-binding sites that occurred at the euchromatin domain boundaries in all the cell types was higher than the number of the randomly generated sites (Figure 5B–E).

In spite of the significant overlap of CTCF-binding sites between the cell types (Figure S2), there was almost no overlap in the barrier CTCF-binding sites across the five cell types examined (Figure 5F). In addition, most of the barriers (57%) occurred in the intergenic regions in all cells (Table S12). Motif analysis of these barrier CTCF-binding sites revealed consensus DNA-binding motifs in all five cell lines, and these were identical to the motif found for all the ubiquitous CTCF-binding sites (Figure 2F and Figure S16). No secondary motifs associated with the barrier CTCF-binding sites were identified. The results were independent of the distance between CTCF-binding sites and the domain boundaries in the definition of barrier CTCF-binding sites.

#### Chromatin domains are mediated by CTCF loops

To determine whether the barrier CTCF-binding sites function through a looping mechanism, we analyzed the CTCF-mediated chromatin interactome in K562 cells using the data from a previous study that mapped the long-range interactions across the human genome in K562 and GM06990 cells with the Hi-C method [58]. The intrachromosomal interactions between barrier CTCF-binding sites of adjacent domain boundaries were much stronger than all the interactions across the human genome of K562 cells (0.0321 vs. 0.0081, *p* = 1.6620E–64; Figure 5F), and even stronger than the interactions between any CTCF-binding sites across the human genome of K562 cells (0.0321 vs. 0.0150, *p* = 1.4483E–38; Figure 5F). Furthermore, the interactions between barrier CTCF-binding sites of adjacent domain boundaries were stronger than any interactions between any barriers across the human genome of K562 cells (0.0321 vs. 0.0168, *p* = 2.7179E–37; Figure 5F).

### CTCF functions in DNA Replication

#### Identification of replication time zones

We next explored the relationship between CTCF-binding sites and replication timing. To this end, we determined genome-wide DNA replication timing for the BJ fibroblast, GM06990, and K562 cell lines by Repli-Seq [59]. We simplified the data for each cell type by combining the six fractions (G1, S1, S2, S3, S4, and G2) that span all of the DNA synthesis phase of cell division into early- (G1+S1), middle- (S1+S2+S3+S4), and late- (S4+G2) replicating DNA. To characterize and compare CTCF-binding sites within different replication patterns genome-wide across all cell types, we identified early-, middle-, and late-replicating zones using HMMSeg (Materials and Methods). In BJ cells, a total of 7,296 replication zones were detected that typically ranged from 29 kb to 2,434 kb (5^th^–95^th^ percentiles) with a median length of 192 kb, although the size distribution depended on the replicating domain type (Table S13). Our results are consistent with previous studies, which estimated that the typical size of a replication zone is in the range of 0.5–2 Mb [60], [61]. However, very large replicons were also detected. In total, 1158 domains were larger than 1 Mb and 74 were larger than 5 Mb, with the largest domain being 62.8 Mb.

#### CTCF is enriched within replication zones

**Figure 6:**
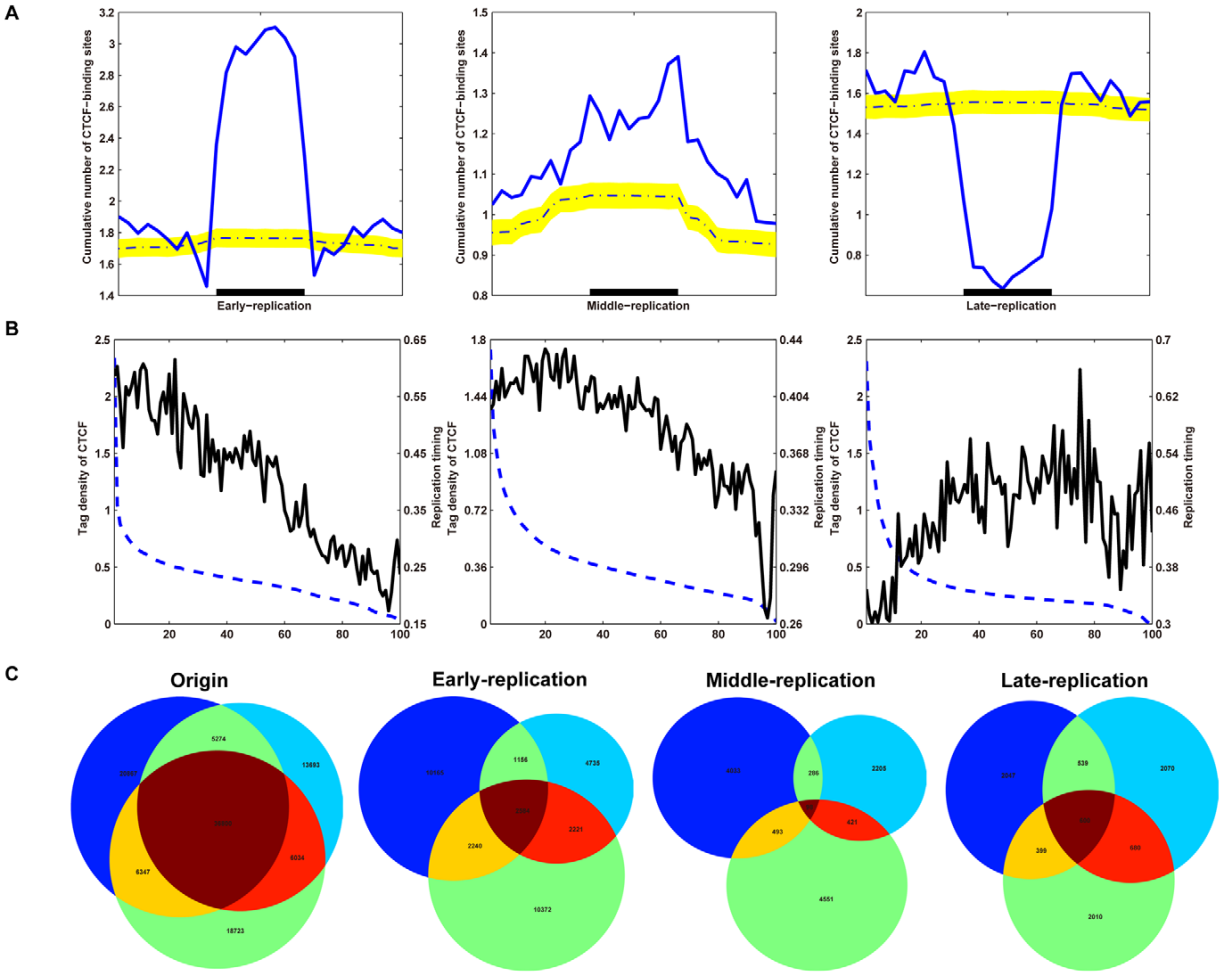
Characteristics of CTCF-binding sites within DNA replication zones. (A) Cumulative number of CTCF-binding sites within replicating zones. The cumulative normalized number of CTCF-binding sites within early-replicating zones (left), middle-replicating zones (middle) and late-replicating zones (right) was plotted to allow comparison of the densities of CTCF-binding sites and shuffled CTCF-binding sites within replicating zones. The intensity plots show the significantly different patterns of the CTCF-binding sites and shuffled CTCF-binding sites. The yellow strip represents the region of 5^th^ and 95^th^ percentiles for the intensity profile of the 10,000 shuffled CTCF-binding sites. The dash-dot line represents the median intensity profile corresponding to the 10,000 shuffled CTCF-binding sites. (B) Correlation between CTCF and replication time. Early-replicating zones (left), middle-replicating zones (middle) and late-replicating zones (right) were grouped into 100 sets (dotted line) based on their levels (from high to low, left to right on the *x*-axis). The average tag density of CTCF was calculated for each group and plotted according to the average tag density of CTCF (right *y*-axis) and the replicating time (left *y*-axis). (C) CTCF-binding sites within replicating zones are cell type-specific. The origin Venn-diagram represents the overlap of all the CTCF-binding sites between the BJ, GM06990, and K562 cells. The Early-, Middle-, and Late-replication Venn-diagram respectively represents the overlap of the CTCF-binding sites that located within Early-, Middle-, and Late-replicating zones between the BJ, GM06990, and K562 cells.

To delineate the nature of CTCF within replicating zones further, we analyzed CTCF-binding sites in each type of replicating zone, and compared the cumulative number of CTCF-binding sites with the number of sites immediately outside the replicating zones (to the left and to the right). When we compared the cumulative number from all replicating zones with the cumulative number of profiles determined from shuffled CTCF-binding sites (Figure 6A), we found unique enrichment patterns of CTCF-binding sites within the early- and middle-DNA replicating zones and at the corresponding boundaries, as well as a pattern of CTCF-binding site depletion within late-replicating zones and the boundaries (Figure 6A). These observations were consistent across cell types (Figure S17).

#### CTCF-binding sites correlate with replication timing

To examine the correlation between CTCF-binding sites and replication timing, we separated each combined fraction of replication time into 100 groups based on CTCF RPKM values (Figure 6B). These groups were then plotted against their average RPKM values determined by Repli-Seq. CTCF-binding sites correlated positively with early- and middle-replicating times genome-wide across cell types, but correlated negatively with late-replicating time zones across cell types.

#### CTCF-binding sites within replicating zones are cell type-specific

We next performed overlap analysis of CTCF-binding sites with each type of replicating zone in BJ, GM06990, and K562 cells (Figure 6C). Interestingly, there was almost no overlap in the CTCF-binding sites localized within different categories of replication zones between the cell types (Figure 6C), in spite of the significant overlap of CTCF-binding sites between the BJ, GM06990, and K562 cells (Figure 6C). This was especially significant for middle-replicating zones, in which only 18%, 27%, and 18% of the CTCF-binding sites in the BJ, GM06990, and K562 cells, respectively, overlap, whereas over 70%, 78%, and 72% of all the CTCF-binding sites in BJ, GM06990, and K562 cells, respectively, overlap (Figure 6C).

## Discussion

In our efforts to better understand the functions of CTCF-binding sites in the human genome, we have identified cell type-specific and ubiquitous CTCF-binding sites in the human genome of 38 cell types and characterized the unique distribution and sequence features of each type of binding site. These cell type-specific and ubiquitous CTCF-binding sites show uniquely versatile transcriptional functions and characteristic chromatin features. Our results not only confirm the well-documented insulator barrier function of CTCF-binding sites, but also explore a novel role in DNA replication.

### CTCF-binding sites are uniquely distributed in the human genome

A total of ∼326,840 CTCF-binding sites were identified in the 38 cell lines examined. Although the majority of these CTCF-binding sites were largely invariant between cell types (ubiquitous CTCF-binding sites), ∼126,200 CTCF-binding sites were cell type-specific. Both ubiquitous and cell type-specific CTCF-binding sites were universally present throughout the genome, and the chromosomal distribution of CTCF-binding sites correlated strongly with genes. The strong correlation with genes is a feature generally associated with transcription factors and suggests widespread function of CTCF in the genome.

The vast majority (>50%) of CTCF binding appeared to occur remotely from the TSSs, while 30% of CTCF-binding sites were located in the introns. Interestingly, ubiquitous CTCF-binding sites were located predominately within intergenic regions, consistent with their potential role as insulators. However, cell type-specific CTCF were located predominately in the introns, suggesting that coexistence with transcription might be a common property of insulators. The significance of CTCF-binding sites located within the introns and exons is not clear, but may relate to the function of insulators in blocking enhancers and silencers that are present near these sequences.

These results demonstrate that CTCF-binding sites display unique distribution patterns and are found throughout the genome. This genome-wide location agrees with previously published mapping of *Drosophila* CTCF- and human CTCF-binding sites performed with ChIP [38], [39], [62]. However, as we were unable to reach saturation levels of CTCF-binding sites with 38 cell lines, future studies must include additional cell lines to identify the majority of the CTCF-binding sites that exist in the entire human genome.

Based on the distance between adjacent CTCF-binding sites, we defined and identified CTCF-clusters in the human genome across cell types and determined that CTCF-clusters with three or more overlapping members most likely represent real CTCF-cluster events. This clustering is reminiscent of the insulator bodies described in the nuclear periphery of *Drosophila* cells, and therefore, might facilitate recruitment of the locus to a nuclear territory inhospitable to transcription [3], [63]–[66]. The formation of insulator bodies might aid in insulator function by sequestering the insulator to a nuclear compartment rich in remodellers and modifiers [46]. This finding is also consistent with the suggestion that insulators interact with one another to organize chromatin loops, although these interactions have not been shown to be necessary for enhancer blocking at this time [67].

### CTCF is a versatile regulator of transcription

As expected, conservation scores and GC content were significantly higher in ubiquitous CTCF-binding sites than in cell type-specific CTCF-binding sites. Correlation analysis of the relationship between CTCF-binding sites and gene expression indicates that CTCF is involved in active gene regulation. It is also notable that the CTCF signal peaked near the 5′ and 3′ ends of genes, and this may represent a useful method by which to confirm annotated TSSs, to identify novel TSSs, or to determine alternative TSS functioning in particular cell types [68].

A surprising, yet consistent, result obtained from both GO analysis and GOMO analysis illustrates that many biological processes, including cellular processes, metabolic processes, and biological regulation, may be regulated by CTCF. Of course, these annotations are often general, and one must return to the original publications in order gain detailed understanding of the functions of CTCF in these processes.

One of the surprising findings of our study is that over 90% of the ubiquitous CTCF-binding sites were characterized by a specific 20-mer consensus motif. While only 27% of cell type-specific CTCF-binding sites shared this consensus motif, many other transcription factors were found within these unique CTCF-binding sites. This indicates that additional motifs along the genome may be recognized by the CTCF-binding protein. Indeed, it is important to note that nearly 30% of all CTCF-binding sites identified did not contain the characterized 20-mer consensus motif. Therefore, it is possible that CTCF binds to different classes of DNA sequences, either directly or in association with a partner. These findings suggest that CTCF is an evolutionarily conserved, yet versatile transcriptional regulator.

### Chromatin signatures determine cell type-specific gene expression

At least 20 well-positioned nucleosomes flanked the ubiquitous CTCF-binding sites examined; however, no well-positioned nucleosomes flanked cell type-specific CTCF-binding sites. These results suggest that the chromatin architecture at cell type-specific CTCF-binding sites is also cell type-specific. Furthermore, ubiquitous CTCF-binding sites bound to a linker region between two well-positioned nucleosomes, and the positioned nucleosomes extended on either side of the CTCF-binding site. The center-to-center distance of neighboring nucleosomes was 185 bp, on average, and largely invariant. Given that 147 bp of DNA is observed in the crystal structure of nucleosomes [69], we can deduce that the length of human linker DNA is 38 bp. Although this linker length is somewhat shorter than the previous estimate of 70 bp in higher eukaryotes [70], it is completely consistent with the most recent robust estimate in the human genome [71]–[73].

Characteristic chromatin features, including a sharp elevation of the number of open chromatin and histone modifications associated with active transcription, were observed within cell type-specific and ubiquitous CTCF-binding sites. In addition, CTCF-binding sites were largely unmethylated. However, compared with ubiquitous CTCF-binding sites, cell type-specific CTCF-binding sites had much higher methylation levels.

A previous study demonstrated that DNaseI HS sites colocalize with histone modified regions, p300 binding regions, and gene expression in a cell type-specific manner [56], thus we hypothesized that these characteristic features of chromatin structure cooperate with transcriptional regulatory elements to determine cell type-specific gene regulation. Analysis of the colocalization of chromatin structure (DNaseI HS sites, histone modifications), regulatory elements (CTCF-binding sites, enhancers, and promoters), and gene regulation verify our hypothesis.

### CTCF organizes higher-order chromatin structure

CTCF is known to demarcate boundaries between euchromatin and heterochromatin [9]. The barrier function of CTCF was originally identified based on the presence of DNaseI HS and specific histone modification-binding sites located at transitions between open and condensed chromatin domains [74]. We identified the heterochromatin and euchromatin domains across the genome, and these domain borders exhibited unique chromatin features. Our analysis of CTCF boundaries indicates enrichment of CTCF-binding sites at the chromatin domain boundaries. CTCF is known to have insulator activity [13] and is therefore a prime candidate to have a function in the demarcation of chromatin domains. Indeed, the sharp changes in CTCF chromatin pattern, including CTCF signal, number of CTCF-binding sites, and the occurrence of the CTCF consensus motif across chromatin boundaries indicate that CTCF functions as a domain barrier that separates the heterochromatin and euchromatin domains. This further confirms the results of previous studies, which demonstrated that insulators delimit distinct organizational domains of a genome [2], [41]. In addition, we found that, on average, over one-fifth of CTCF-binding sites act as domain barriers across cell lines, and barrier CTCF-binding sites associate with the domain boundaries in a cell type-specific manner.

Another striking finding of our study is that the intrachromosomal interactions between barrier CTCF-binding sites of chromatin domains are significantly stronger than all the interactions across the human genome, the interactions between all CTCF-binding sites across the human genome, and the interactions between all barriers across the human genome. This finding suggests that CTCF-mediated chromatin interactions may produce loops that act as structural and functional barriers. This information agrees with the findings of another recent study, which uncovered five distinct chromatin domains characterized by looping interactions connected via CTCF [75]. Taken together, these findings extend our understanding of higher-order chromatin organization and plasticity, and lay the foundation for a better understanding of mammalian gene regulation.

### CTCF is involved in DNA replication

This study reveals that, in addition to its known barrier function, CTCF is closely associated with DNA replication, especially early- and middle-replication. Furthermore, CTCF-binding sites enriched within replication zones are highly cell type-specific. Previous studies suggested that the function of *Drosophila* insulators during DNA replication might be similar to their function during transcriptional regulation [76], [77]. Therefore, it is plausible that insulators could be involved in delineating separate replication domains with distinct replication timing and regulation, a role that would presumably entail maintenance of insulator function throughout S phase [78]. However, precisely what happens when the insulator site itself is replicated remains unclear.

In conclusion, we have provided a comprehensive and systematic study revealing new functions of cell type-specific and ubiquitous CTCF-binding in the human genome. Our results provide a much-needed resource for further investigation into the role of CTCF in chromatin insulation, gene regulation, higher-order chromatin organization, and DNA replication.

## Materials and Methods

### Data source for computational analysis

All sequence and peak data of different cell lines used in this study are freely available for download from different tracks in the UCSC NCBI37/hg19 Genome Browser (http://genome.ucsc.edu/encode/), including UW Affy Exon, Duke DNaseI HS, UNC FAIRE, UW DNaseI DGF, UW DNaseI HS, HAIB Methyl RRBS, Broad Histone, UW Histone, UW CTCF Binding, and Nucleosome positioning. The gene annotations presented herein were obtained from the GENCODE data in the GENCODE Genes track (manual version 4, May 2010). The phastCons and phyloP scores were extracted from the hg19 Conservation track of the UCSC Genome Browser. The strong enhancers and active promoters were obtained from recent studies [79], [80], and are freely available through the “Broad HMM” track in the UCSC hg19 Genome Browser. All chromosome Y data were omitted from this study. Complete data of the hg19 human genome in K562 cells were illustrated as circles using Circos [81] in Figure S18. All the raw data used in this study are released currently. In addition, the use of these data are strict adherence to the ENCODE Consortium Data Release Policy.

### Lineage specificity and classification of CTCF-binding sites

To test whether we can determine cell type-specificity from CTCF-binding sites, we clustered the CTCF-binding sites in 38 cell types and classified CTCF-binding sites according to their occurrence rates in the 38 cell lines. A given CTCF-binding site was classified as “cell type-specific” if it did not overlap (where overlap between two binding sites is recognized when two regions have at least one common base pair) with any CTCF-binding site within the other 37 cell lines. A given CTCF-binding site was classified as “ubiquitous” if it overlapped with any CTCF-binding site from any of the 38 cell lines. The remaining CTCF-binding sites, present in 2–37 of the cell lines, were classified as “common.”

### CTCF-binding site saturation

The number of CTCF-binding sites was computed for each of the different types (cell type-specific, common, and ubiquitous) as a function of the number of cell lines tested. A single cell line (designated as #1) was selected randomly and the total number of CTCF-binding sites was calculated. Then, single cell lines were added sequentially, and the number of CTCF-binding sites in each newly-added cell line that did not overlap with previous CTCF-binding sites was added to the total set. In order to determine the total number of cell type-specific CTCF-binding sites, the CTCF-binding sites from each of the sequentially added cell lines that overlapped with CTCF-binding sites from the previous cell lines were discarded and not added to the total set. In contrast, to determine the total number of ubiquitous CTCF-binding sites, the CTCF-binding sites from each of the sequentially added cell lines that did not overlap with the previously identified CTCF-binding sites were discarded and not added to the total set. The computation was complete when all of the 38 cell lines had been included in the analysis. The above procedure was repeated at random 10,000 times and the resulting values were normalized at each point of new cell line introduction.

### Control sets of shuffled CTCF-binding sites

We used the shuffleBed command in BEDTools [82] with the “- chrom” option to permute the locations of different types of CTCF-binding sites within the human genome (hg19), allowing performance of statistical analysis. For each type of CTCF-binding site BED file for a cell line, we generated over 10,000 corresponding shuffled CTCF peak BED files.

### Densities of gene and CTCF-binding sites

The entire genome was scanned with 2 Mbp windows and, within each window, the number of genes and types of CTCF-binding sites were quantitated. Linear regression was used to determine the correlation between gene density and binding site density. The CTCF-binding sites correlate strongly with genes for each chromosome. For example, with a correlation coefficient of 0.7437 (*p* = 2.9494E–23) in chromosome 1 of K562 cells (Tables S4B and S4C). In contrast, the average correlation coefficient between randomly generated genomic sites and genes is only 0.0140 (*p* = 0.8770) (Table S4D).

### Identification and characterization of CTCF-clusters

For each cell type, we sorted all CTCF-binding sites by their genomic location. The distance between adjacent CTCF-binding sites was determined. The CTCF-binding sites were classified as CTCF singletons if the distance from adjacent CTCF-binding sites was larger than the threshold of 10 kb. Otherwise, the CTCF-binding sites were classified as CTCF-clusters. CTCF-2 denotes CTCF-clusters with two CTCF-binding site members, CTCF-3 denotes clusters with three CTCF-binding site members, and so forth.

A Monte Carlo simulation was performed to assess the background level of CTCF-binding site clustering when mapped to the genome. In the simulation, we first randomly permuted the locations of CTCF-binding sites (the same number and size; see Control sets of shuffled CTCF-binding sites subsection, above) within the human genome assembly hg19, and then determined how many CTCF-binding sites clustered with others as described above. This process was repeated over 10,000 times to compute the percentage of randomly shuffled CTCF-binding sites that clustered. The result is summarized in Table 1. Based on this simulation in K562 cells, we estimated that 5,830 CTCF-binding sites (8.58% of total) would result in three overlapping CTCF-binding sites (CTCF-3), 1,867 in CTCF-4, 606 in CTCF-5, 202 in CTCF-6, and so forth due to random chance. In contrast, the number of experimentally generated overlapping CTCF-binding sites was significantly higher than the estimated background. Therefore, it is highly likely that clustering CTCF-binding sites resulted from the effect of immunoprecipitation rather than from random events. This result is independent of threshold, illustrating robustness of the intrinsic nature of CTCF-clusters.

### Quantification of tag densities

To avoid potential variability in signal and background related to tag mapability variation, sequence bias, or binding length, we quantified tag density in reads per kilobase per million mapped reads (RPKM) [83]. For each sequencing data, we computed RPKM value (*R_k_*) as *R_k_* =  (10^9^•*N_k_*)/(*N*•*L_k_*), where *N_k_* is the number of nonredundant unique reads in region *E_k_*, and *L_k_* is the length of *E_k_*, and *N* is the total number of nonredundant unique sequenced reads. The RPKM continuous profiles were quantile normalized [84] and mean values for replicates were calculated.

We calculated the profiles of tag density of chromatin features, including DNaseI HS, and histone modifications, near each type of CTCF-binding site with 150 bp windows at a resolution of 10 bp, except for nucleosome positioning, which was calculated at single nucleotide resolution. The area scanned spanned the 3 kb immediately upstream of the CTCF-binding start site, the CTCF-binding site, and the 3 kb downstream from the end of the CTCF-binding site. Each window was evaluated for the tag densities of chromatin features. All window tag counts were normalized to the total number of bases present in the window and to the total read number of the given library.

To plot the profiles of those CTCF-binding sites associated with TSSs, the ∼29,580 GENCODE genes with expression information were categorized into broad groups according to their reported expression levels: high, median, or mainly silent. Two thousand genes were selected per group and corresponding CTCF ChIP-Seq data was analyzed after each was aligned by their TSS. The genomic region that was analyzed encompassed the entire defined gene body (exons and introns), and extended 5 kb upstream and 5 kb downstream of the 5′ and 3′ boundaries. RPKM values in the gene body were quantitated in windows representing ten equal parts, and in 0.2 kb windows in the 5′ and 3′ proximal regions. RPKM values for each window were then evaluated for each gene and normalized by the total number of genes in each group.

### Evolutionary conservation of CTCF-binding sites

Both phastCons and phyloP scores were collected across the complete CTCF peak site and unoccupied CTCF-binding site (controls), which did not overlap any CTCF sites across 38 cell types. The average conservation scores were calculated for multiple alignments of 45 vertebrate genomes to the human genome (vertebrate), plus an alternate set of scores for the primate subset of species in the alignments (primate), and an alternate set of scores for the placental mammal subset of species in the alignments (placental).

The average GC content was calculated for each CTCF-binding site and unoccupied CTCF–binding site (controls). The GC sequence data were extracted from the hg19 GC Percent track of the UCSC Genome Browser. A two-sided Wilcoxon rank sum test was performed to analyze conservation scores and GC content at CTCF-binding sites and the corresponding controls.

### Expression level within CTCF-binding sites and genes

RNA expression data (Affymetrix Exon Array from University of Washington) were obtained for each cell type by calculating the log2 ratio of the exon expression level relative to the median expression for all cell types. The positions of probes from the Affymetrix Human Exon 1.0 ST array were mapped onto the CTCF-binding sites. If a CTCF-binding site contained at least one probe and had valid expression data for all cell types examined, it was included in the analysis. For each cell line, a single expression score was calculated for each included CTCF-binding site that represented the average expression of all probes mapped to that binding site. For each cell line the distribution of expression of each CTCF-binding site, and the corresponding RPKM value of CTCF-binding site was examined. Cell type-specific, common, and ubiquitous CTCF-binding sites were divided among into 100 groups according to expression level. The average expression level and RPKM value were calculated for each group.

The positions of probes from the Affymetrix Human Exon 1.0 ST array were mapped onto the transcript regions in GENCODE. If a transcript region contained at least one probe and had valid expression data for all cell types examined, it was included in analysis. For each cell line, a single expression score was calculated for each included transcript region that represents the average expression of all probes mapped to that transcript region. For each cell line, the distribution of expression in transcript regions was examined, and 2,000 sites displaying significantly high, median, low and silent expression relative to the median expression for all cell types (based on a normal distribution) were retained for each cell type, respectively.

### Scatter correlations of CTCF binding-sites with genome annotations

Cell type-specific, common, and ubiquitous CTCF-binding sites were divided into 100 groups according to the CTCF-binding signal. The average RPKM values of CTCF-binding and of genome annotations, including nucleosome positioning, histone modifications, and open chromatin were calculated for each group. Correlations between CTCF-binding sites and genome annotations were evaluated by Pearson's correlation.

### GO analysis of proximal CTCF-binding sites

To determine whether consistent biological themes could be identified among different cell types, we identified the genes related to proximal CTCF-binding sites (<1 kb from TSS) for each cell type, and used the gene lists from each cell type to identify enriched GO categories (EASE score <0.05) using EASE [47]. For all enriched GO categories from the 38 cell types, we generated the GO matrix, and the value of each entry is the EASE score. During the generation of the lists of genes, we noted a marked improvement in concordance (percent overlap in genes based on pair-wise comparisons of gene lists across cell types) of gene lists from ubiquitous CTCF-binding sites, however, we noted a marked depletion in concordance of gene lists from cell type-specific CTCF-binding sites. This is expected, since ubiquitous CTCF-binding sites were consistent across cell types, whereas cell type-specific CTCF-binding sites were unique to each cell type. Hierarchical clustering of both the cell types and the common GO nodes was performed based on the calculated EASE scores using the software Cluster 3.0 with average linkage [85]. Similarly, we performed GO analysis for proximal CTCF-binding sites that are cell type-specific and ubiquitous across the 38 cell types, and generated the corresponding GO matrix.

### Analysis of CTCF-binding site motifs

For motif identification, we examined data of CTCF-binding sites that encompassed defined ChIP-enriched regions in each cell line. MEME (Version 4.4.0) [48] was used to discover consensus motifs with default parameters. MEME was instructed to report the top five motifs with lengths of 20 bases. To determine the number of peaks that could be explained by statistically significant motifs, the MEME tool MAST [86] was used to estimate the maximal difference between the total number of peaks containing a motif and the number that could be explained by chance within a range of stringencies (*E*-values). MAST curves were generated using the method described in previous studies [45]. To identify known TF motifs with similarity to the newly discovered motifs, we used TOMTOM [49] to scan the collections of previously discovered motifs in JASPAR [50], TRANSFAC [51] and UniPROBE [52] databases with *q*-value <0.05. To detect associations between TF-binding motifs and GO terms, the MEME tool GOMO [53], [54] was used to assign functional roles to TF-binding motifs.

### Colocalization analysis among binding sites and genes

We performed colocalization analysis on two types of binding sites or regions in *N*-by-*N* (*N* is the number of cell lines) cell line combinations as similarly described in previous study [56]. Ubiquitous and common binding sites often overlap with each other; thus, we excluded these ubiquitous and common binding sites in the colocalization analysis, since they merely increase the counts of overlap. The counts were divided by the corresponding row sum and column sum and multiplied by the matrix sum to obtain enrichment values, which is done in the same way as the 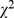 test. We plotted the enrichment factor for each histone modification in a *N*-by-*N* grid with a bubble chart.

### Identification of chromatin domains

To explore the boundaries of chromatin domains in a uniform way across multiple cell types, we collected genome-wide chromatin data sets generated by ChIP-Seq and DNaseI-Seq. Five human cell types, designated by the ENCODE consortium [42]–[44], were used in this analysis. These included erythrocytic leukaemia cells (K562), B-lymphoblastoid cells (GM12878), umbilical vein endothelial cells (HUVEC), normal epidermal keratinocytes (NHEK), and mammary epithelial cells (HMEC). We used antibodies specific for histone H3 lysine 4 trimethylation (H3K4me3), a modification associated with promoters [56], [87]–[89]; H3K4me2 (dimethylation), associated with promoters and enhancers [39], [44], [89], [90]; H3K4me1 (methylation), preferentially associated with enhancers [44], [90]; lysine 9 acetylation (H3K9ac) and H3K27ac, associated with active regulatory regions [89], [91]; H3K36me3, associated with transcribed regions [39], [87], [88]; H3K27me3, associated with Polycomb repressed regions [39], [87]; and DNaseI HS, associated with open chromatin [45], [56], [92].

To identify heterochromatin and euchromatin domains, the RPKM continuous profiles of diverse histone modifications and open chromatin were quantile normalized and used as inputs to HMMSeg [57]. HMMSeg was run with the following parameter settings: smooth  = 16,000, num-states  = 2, num-starts  = 10, and maxiter  = 100. Default values were used for the rest of the parameters. This resulted in, on average, 18,217 euchromatin domains covering 870,646,600 (28.67%) bases and 18,234 euchromatin domains covering 2,165,668,400 (71.33%) bases across five cell types (Table S11).

### Analysis of chromatin properties near identified chromatin boundaries

To characterize the general properties of euchromatin domains and their borders, we aligned all 14,166 euchromatin domains identified in K562 cells by their left or right border and calculated the average log2 ratio RPKM profiles of various genomic and chromatin parameters across the combined 28,332 borders (left and mirrored right border regions combined). The average profile of DNaseI HS reflected the abrupt change in signal at these chromatin boundaries (Figure S14). This analysis was used to investigate the three states of H3K4 methylation, H3K9 and H3K27 acetylation, H3K36 trimethylation, and H4K20 monomethylation surrounding the chromatin boundaries. The mean levels of these histone modifications inside euchromatin domains are significantly larger than outside (Figure S14). Strikingly, abrupt transitions in these histone modification profiles occur exactly at the chromatin borders. We also investigated H3K27me3, a histone modification found in different types of heterochromatin [93]. H3K27me3 is substantially enriched in heterochromatin (Figure S14), which is consistent with the frequent association of this mark with repressed genes [94].

### Identification of barrier CTCF-binding site

A CTCF-binding site was defined as a barrier site relative to a euchromatin domain only if the distance between the CTCF-binding site and the domain boundary was at most 8 kb. In order to assess the possibility that the identified barrier CTCF-binding sites colocalize with domain boundaries just by chance, we counted the number of shuffled CTCF-binding sites classified as barrier sites. The *p*-value was then the fraction of times (over 10,000 trials) that the number of CTCF-binding sites classified as barrier sites in the random trial experiment was at least as much as the observed number of barrier CTCF-binding sites. The smaller the fraction (*p*-value), the higher the significance. The *p*-values were <1E–05 for barrier CTCF-binding sites across cell types.

### Intrachromosomal interactions between barrier CTCF-binding sites

For the intrachromosomal interactions analysis of barriers, the barrier CTCF-binding sites in the NCBI37/hg19 Genome assembly were first converted to NCBI36/hg18 Genome assembly using the liftOver tool at the UCSC Genome Browser [95]. From the 22,280,372 intrachromosomal interactions across the human genome in K562 cells obtained with the Hi-C method from an earlier study [58], we extracted a total of 9,865,455, 540,839, and 1,686 interactions between any CTCF-binding sites, any barrier CTCF-binding sites, and barriers of adjacent chromatin domain boundaries.

### Analysis of replication timing data

Raw sequencing reads for replication timing datasets were downloaded from the Sequence Read Archive (SRA) (http://www.ncbi.nlm.nih.gov/sra) (study accession number SRP001393.1), and were aligned to the human reference genome (hg19) using version 0.12.7 of Bowtie [96]. Unique reads containing up to two mismatches were mapped to the genome. The post-processing of the aligned data was performed following the methods detailed in a previous study [59]. Mapped sequence tags containing simple repeats and other low-complexity sequences appeared to be nonspecific background regions (“bad spots”) and were filtered by calculating sequence tag densities in 150 bp windows and removing tags within windows containing five or more tags. After filtering bad spots, the tag density of BrdU-DNA-derived sequence tags along the genome was calculated for each cell-cycle fraction using 50 kb sliding windows at 1 kb intervals. To simplify the computational analysis of replication timing in different cell lines, we averaged the tag density RPKM value for G1 and S1 for each 1000 bp window of the genome to yield a cumulative “early” replication signal for each cell type. Similarly, a “middle” signal was calculated by averaging the tag density RPKM value from S1 to S4 for each position and for each cell type; a “late” signal was calculated by averaging the tag density RPKM value for S4 and G2 for each position and for each cell type. To avoid spurious signals, regions including gaps, segmental duplications, and the entire Y chromosome were removed from the analysis.

To identify replication timing domains, the RPKM continuous profiles of different cell cycle fractions of replication timing were used as input to HMMSeg, with the following parameter settings: num-states  = 2, num-starts  = 10, and maxiter  = 100. Default values were used for the rest of the parameters. Since the profiles of replication timing were smooth enough (50 kb sliding windows at 1 kb intervals), no wavelet smoothing was required of replication timing.

## Supporting Information

Figure S1 **Lineage specificity of CTCF-binding sites across 38 cell types.** ChIP-Seq density heatmap representing all CTCF-binding sites across 38 cell types. Site order was first determined by highest occurrence rates in 38 cell lines and arranged from highest to lowest (38/38 to 1/38). Cell type-specific and ubiquitous CTCF-binding sites are grouped at the bottom and top, respectively. Within each occurrence rate, site order was determined by highest average ChIP-Seq density in cell lines and arranged from highest to lowest density. The binding site and the genomic region from –3 kb to +3 kb, relative to the CTCF-binding sites, are shown. Cell lines were clustered based on their CTCF-binding site using Ward's hierarchical clustering with Cluster 3.0. C1  =  AG04449; C2  =  AG04450; C3  =  AG09309; C4  =  AG09319; C5  =  AG10803; C6  =  AoAF; C7  =  BJ; C8  =  CACO2; C9  =  GM06990; C10  =  GM12801; C11  =  GM12864; C12  =  GM12865; C13  =  GM12872; C14  =  GM12873; C15  =  GM12874; C16  =  GM12875; C17  =  GM12878; C18  =  Hasp; C19  =  HBMEC; C20  =  HCFaa; C21  =  HCPE; C22  =  HEE; C23  =  HEK293; C24  =  Helas3; C25  =  HepG2; C26  =  HL60; C27  =  HMEC; C28  =  HMF; C29  =  HPAF; C30  =  HPF; C31  =  HRE; C32  =  HRPE; C33  =  HUVEC; C34  =  K562; C35  =  NHEK; C36  =  SAEC; C37  =  SKNSHRA; C38  =  WERIRB1.
(PDF)

Figure S2 **Overlap of CTCF-binding sites across 38 cell types.** Heatmap representing the overlap between CTCF-binding sites across the 38 cell types examined. Cell lines were clustered based on their CTCF-binding site using Ward's hierarchical clustering with Cluster 3.0.
(PDF)

Figure S3 **Genome-wide distribution of strongest and weakest scoring CTCF-binding sites relative to cell type.** The proportion of CTCF-binding site types in the K562 cell line among the strongest scoring (top 20%), and weakest scoring (bottom 20%) CTCF-binding sites are shown. In the strongest scoring CTCF-binding sites, 81% were found to be ubiquitous and almost none (0.7%) were cell type-specific. In contrast, in the weakest scoring CTCF-binding sites, 19% were cell type-specific and almost none (0.6%) were ubiquitous.
(PDF)

Figure S4 **Scatter correlation between CTCF-binding sites and gene expression across cell types.** CTCF-binding sites were grouped into 100 sets (dot) based on the gene expression levels (from high to low, left to right on the *x*-axis). The average tag densities of CTCF and the average gene expression levels were calculated for each group and plotted according to the average gene expression level (left *y*-axis) and the average tag densities of CTCF (right *y*-axis).
(PDF)

Figure S5 **GO analysis of proximal CTCF-binding sites across 38 cell types.** (A) Clustering of 38 cell types based on common GO nodes. This resulted in a list of 243 common GO nodes. Hierarchical clustering of both the cell types and the common GO nodes was performed using the calculated EASE scores. The relationship between the color intensity and EASE score is illustrated by the color bar. Gray indicates that an EASE score was not calculated for that GO node. The cell type is denoted by the letter and number combination at the top of every column. C1–C38  =  CTCF-binding sites of 38 cell types, U  =  ubiquitous CTCF-binding site. (B) Summary of biological processes regulated by genes related to the proximal CTCF-binding sites across 38 cell types. Annotations were obtained from the Gene Ontology database.
(PDF)

Figure S6 **GOMO analysis of over-represented motifs within cell type-specific and ubiquitous CTCF-binding sites across 38 cell types.** (A) GO analysis of the first three over-represented motifs within cell type-specific and ubiquitous CTCF-binding sites. Clustering of 38 cell types based on common GO nodes. This resulted in a list of 443 common GO nodes. Hierarchical clustering of both the cell types and the common GO nodes was performed using the calculated EASE scores. The relationship between the color intensity and EASE score is illustrated by the color bar. Gray indicates that an EASE score was not calculated for that GO node. The cell type is denoted by the letter and number combination at the top of every column. C1–C38  =  CTCF-binding sites of 38 cell types, U  =  ubiquitous CTCF-binding site. (B) Summary of biological processes regulated by genes related to the first three overrepresented motifs within cell type-specific and ubiquitous CTCF-binding sites. Annotations were obtained from the Gene Ontology database. (C) GO analysis of the first five over-represented motifs within cell type-specific and ubiquitous CTCF-binding sites. Clustering of 38 cell types based on common GO nodes. This resulted in a list of 700 common GO nodes. Hierarchical clustering of both the cell types and the common GO nodes was performed using the calculated EASE scores. The relationship between the color intensity and EASE score is illustrated by the color bar. Gray indicates that an EASE score was not calculated for that GO node. The cell type is denoted by the letter and number combination at the top of every column. C1–C38  =  CTCF-binding sites of 38 cell types, U  =  ubiquitous CTCF-binding site. (D) Summary of biological processes regulated by genes related to the first five over-represented motifs within cell type-specific and ubiquitous CTCF-binding sites. Annotations were obtained from the Gene Ontology database.
(PDF)

Figure S7 **Chromatin structure near the CTCF-binding sites.** Nucleosome (blue lines) and CTCF (red lines) profiles around CTCF-binding sites in K562 cells (A) and GM12878 cells (B) are illustrated. Distances from the CTCF-binding sites are plotted along the *x*-axis. Left and right *y*-axis represents the normalized tag densities of nucleosome and CTCF, respectively.
(PDF)

Figure S8 **Scatter correlations between CTCF and nucleosome positioning in K562 and GM12878 cell types.** Correlation between CTCF and nucleosome positioning in K562 (A–D) and GM12878 (E–H) cell types. Total (A, E), cell type-specific (B, F), common (C, G), and ubiquitous (D, H) CTCF-binding sites were grouped into 100 sets (dot) based on their levels (from high to low, left to right on the *x*-axis). The average tag densities of CTCF and of histone modifications were calculated for each group and plotted according to the average tag density of CTCF (right *y*-axis) and the histone methylation (left *y*-axis).
(PDF)

Figure S9 **Scatter correlations between CTCF and open chromatins and histone modifications in K562 cells.** Cell type-specific (A), common (B), and ubiquitous (C) CTCF-binding sites were grouped into 100 sets (dot) based on their levels (from high to low, left to right on the *x*-axis). The average tag densities of CTCF and of open chromatins were calculated for each group and plotted according to the average tag densities of CTCF (right *y*-axis) and the open chromatin (left *y*-axis).
(PDF)

Figure S10 **Correlation between CTCF and DNA methylation in K562 cells.** Cell type specific (A), common (B), and ubiquitous (C) CTCF-binding sites were grouped into 100 sets (dot) based on their levels (from high to low, left to right on the *x*-axis). The average tag densities of CTCF and of DNA methylation levels were calculated for each group, and plotted according to the average tag density of CTCF (right *y*-axis) and the DNA methylation level (left *y*-axis).
(PDF)

Figure S11 **CTCF-binding sites colocalize with DNaseI HS sites, histone modified regions, enhancers, and promoters in a cell type-specific manner.** CTCF-binding sites colocalize with histone modifications, DNaseI HS sites, strong enhancers and gene expression in a cell type-specific manner. Bubble size represents the level of enrichment. When no bubble is present, the value is zero (complete depletion).
(PDF)

Figure S12 **CTCF-binding sites, DNaseI HS sites, histone modification hits, enhancers, and promoters colocalize with gene expression in a cell type-specific manner.** CTCF-binding sites (A), histone modifications (B–H), DNaseI HS sites (I), enhancers (J), and promoters (K) colocalize with gene expression in a cell type-specific manner. Bubble size represents the level of enrichment. When no bubble is present, the value is zero (complete depletion).
(PDF)

Figure S13 **Identification of chromatin domains in K562 cells.** Circos map of the whole-genome chromatin domains, associated CTCF-binding sites, DNaseI HS, and histone modifications from chromosome 1 to chromosome X, generated using the Circos software package. Chromatin domains were identified using HMMSeg. The outermost circle (circle 1) represents the chromosome band (scale in Kb). Circles 2 and 3 represent the CTCF peaks and tag density profile, respectively. Circle 4 represents the DNaseI HS profile. Circles 5–11 represent the histone modifications H3K27ac, H3K27me3, H3K36me3, H3K4me1, H3K4me2, H3K4me3, and H3K9ac, respectively. Circle 12 represents the euchromatin (medium blue) and heterochromatin (light cyan) domain. Intrachromosomal interactions are drawn in the innermost ring with color intensities (from white to gray) reflecting interaction strength (from low to high).
(PDF)

Figure S14 **Chromatin features around the chromatin borders across cell types.** Profiles of genomic and chromatin features around chromatin borders of GM12878 (A), HMEC (B), HUVEC (C), K562 (D), and NHEK (E) cell types. Log2 ratio profiles of aligned chromatin border regions (all 2,688 borders in K562 cells; left and mirrored right border regions combined) are shown for DNaseI HS, H3K4me1, H3K4me2, H3K4me3, H3K9ac, H3K27ac, H3K36me3, H4K20me1, and H3K27me3. To align chromatin borders, genome-wide positions of all analyzed features were converted to coordinates relative to the nearest border. The area to the left of the dash-dot line and all negative coordinates represent heterochromatin domains; the area to the right of the dash-dot line and all positive coordinates represent euchromatin domains. Blue lines show moving-window averages with window sizes of 16 kb.
(PDF)

Figure S15 **Profiles of CTCF features around chromatin borders across cell types.** (A) CTCF profiles at chromatin boundary. Tag density of CTCF centered on chromatin domain boundaries, and extended 320 kb upstream and downstream of the boundary at 16 kb resolution. Plotted on the *y*-axis is the normalized tag density and on the *x*-axis is distance from the chromatin boundary. (B) CTCF consensus motifs are enriched on the chromatin boundary. Number profiles of CTCF consensus motifs centered on boundaries of chromatin domains, and extended 320 kb upstream and downstream of the boundary at 16 kb resolution. Plotted on the *y*-axis is the normalized number of CTCF-binding sites and on the *x*-axis is distance from the chromatin boundary.
(PDF)

Figure S16 **CTCF consensus motifs identified within barrier CTCF-binding sites across five cell types.** Significantly enriched CTCF-consensus motifs identified within barrier CTCF-binding sites of (A) GM12878, (B) HMEC, (C) HUVEC, (D) K562, and (E) NHEK cells are graphically depicted using WebLogo.
(PDF)

Figure S17 **Cumulative number of CTCF-binding sites within replicating zones across cell types.** Cumulative number of CTCF sites within replicating zones of K562 (A) and GM06990 (B) cell types. The cumulative normalized number of CTCF-binding sites within early-replicating zones (left), middle-replicating zones (middle) and late-replicating zones (right) were plotted for comparison of the densities of CTCF-binding sites and shuffled CTCF-binding sites within replicating zones. The intensity plots show the significantly different patterns of CTCF-binding sites and shuffled CTCF-binding sites.
(PDF)

Figure S18 **Raw data from the K562 cell line.** Circos map of the whole-genome raw data from K562 cells used in this study, created with the Circos software package. The outermost circle (circle 1) represents the chromosome band (scale in Mb). Circles 2–7 represent the replicating time in G1, S1, S2, S3, S4, G2, respectively, with color intensities reflecting their interaction strength (from white to gray). Circles 8 and 9 represent the CTCF peaks and tag density profile, respectively. Circles 10–12 represent the tag density of the DNaseI DGF, DNaseI HS, and FAIRE profiles, respectively. Circle 13 represents the tag density of DNA methylation. Circles 14–26 represent the histone modifications H2A.Z, H3K27ac, H3K27me3, H3K36me3, H3K4me1, H3K4me2, H3K4me3, H3K79me2, H3K9ac, H3K9me1, H3K9me3, H4K20me1, and Pol2, respectively. Circle 27 represents the phastCons 46-way conservation. Circle 28 and 29 represent enhancers and promoters, respectively. Circle 30 represents gene density.
(PDF)

Table S1 **Identification and characterization of CTCF binding-sites across 38 cell types.** (A) The proportion of each CTCF-binding site type across 38 cell types. (B) The proportion of each CTCF-binding site among the strongest scoring (top 20%) CTCF-binding sites across 38 cell types. (C) The proportion of each CTCF-binding site type among the weakest scoring (bottom 20%) CTCF-binding sites across 38 cell types. (D) The proportion of each CTCF-binding site type that located within annotated genes across 38 cell types.
(XLSX)

Table S2 **CTCF-cluster mapping across 38 cell types.**
(XLS)

Table S3 **Statistical analysis of conservation scores and GC content of each CTCF-binding site type across 38 cell types.** (A) The mean values of conservation scores and GC content for each CTCF-binding site type across 38 cell types. (B) Analysis of the statistical significance between each CTCF-binding site across 38 cell types and Unoccupied sites, and between cell type-specific and ubiquitous CTCF-binding sites.
(XLSX)

Table S4 **Correlations between CTCF-binding sites and gene densities, enhancers, and promoters across cell types.** (A) Correlation between CTCF-binding sites and gene density across 38 cell types. (B) Correlation between CTCF-binding sites and gene density along each chromosome across 38 cell types. (C) Correlation between CTCF-binding sites and densities of enhancers and promoters across five cell types.
(XLSX)

Table S5 **Correlations between CTCF-binding sites and gene expression across cell types.**
(XLSX)

Table S6 **Significant GO nodes correlated with cell type-specific and ubiquitous CTCF-binding sites across 38 cell types.** (A) Significant GO nodes across cell type-specific and ubiquitous CTCF-binding site combinations of 38 cell lines. (B) Significant GO nodes across all proximal CTCF-binding site combinations of 38 cell lines.
(XLSX)

Table S7 **Over-represented motifs within ubiquitous and cell type-specific CTCF-binding sites across 38 cell types.**
(DOCX)

Table S8 **MAST analysis of each CTCF-binding site type across 38 cell types.**
(XLSX)

Table S9 **Significant GO nodes of the over-represented motifs correlated with cell type-specific and ubiquitous CTCF-binding sites across 38 cell types.** (A) Significant GO nodes of the first three over-represented motifs within cell type-specific and ubiquitous CTCF-binding site combinations of 38 cell lines. (B) Significant GO nodes of the first five over-represented motifs within cell type-specific and ubiquitous CTCF-binding site combinations of 38 cell lines.
(XLSX)

Table S10 **Correlations between CTCF-binding sites and chromatin features.**
(XLSX)

Table S11 **Identification and characterization of chromatin domains.** (A) Identification and characterization of chromatin domains identified by HMMSeg at a scale of 16 kb. (B) Identification and characterization of chromatin domains identified by HMMSeg at a scale of 8 kb.
(XLSX)

Table S12 **Identification and characterization of barrier CTCF-binding sites.** (A) At a scale of 16 kb, barrier CTCF-binding sites were defined as the CTCF-binding sites located within 16 kb immediately upstream and downstream of the domain boundaries. (B) At a scale of 8 kb, barrier CTCF-binding sites were defined as the CTCF-binding sites located within 8 kb immediately upstream and downstream of the domain boundaries.
(XLSX)

Table S13 **Identification and characterization of replication time zones.**
(XLSX)
