# Supplementary figures and images for: Comprehensive Identification and Annotation of Cell Type-Specific and Ubiquitous CTCF-Binding Sites in the Human Genome
Source: PLoS One. 2012 Jul 19;7(7):e41374. doi: 10.1371/journal.pone.0041374 (PMC3400636; doi:10.1371/journal.pone.0041374)

Figure S1

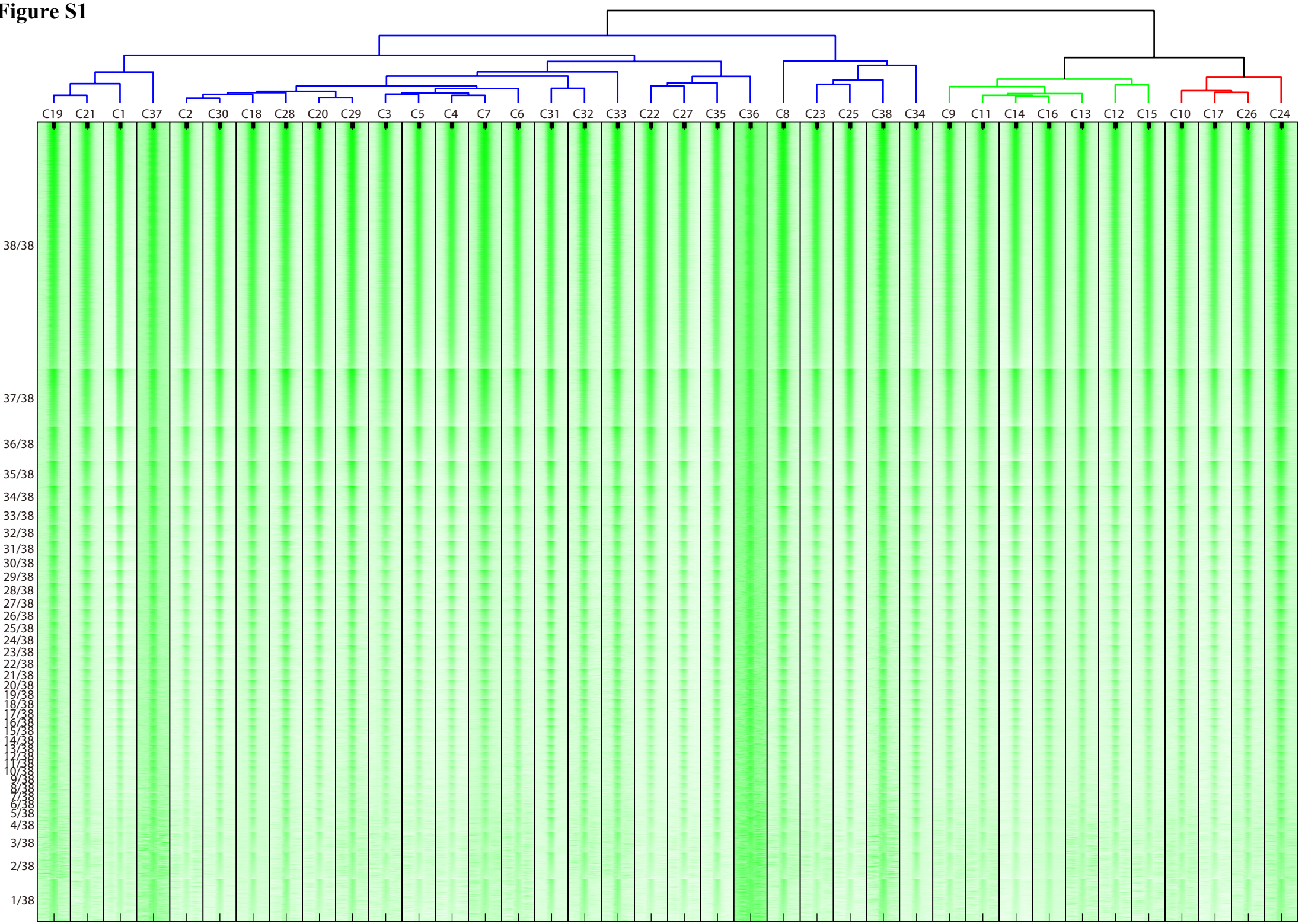

Supplement: Figure S1 — Lineage specificity of CTCF-binding sites across 38 cell types. ChIP-Seq density heatmap representing all CTCF-binding sites across 38 cell types. Site order was first determined by highest occurrence rates in 38 cell lines and arranged from highest to lowest (38/38 to 1/38). Cell type-specific and ubiquitous CTCF-binding sites are grouped at the bottom and top, respectively. Within each occurrence rate, site order was determined by highest average ChIP-Seq density in cell lines and arranged from highest to lowest density. The binding site and the genomic region from –3 kb to +3 kb, relative to the CTCF-binding sites, are shown. Cell lines were clustered based on their CTCF-binding site using Ward's hierarchical clustering with Cluster 3.0. C1 = AG04449; C2 = AG04450; C3 = AG09309; C4 = AG09319; C5 = AG10803; C6 = AoAF; C7 = BJ; C8 = CACO2; C9 = GM06990; C10 = GM12801; C11 = GM12864; C12 = GM12865; C13 = GM12872; C14 = GM12873; C15 = GM12874; C16 = GM12875; C17 = GM12878; C18 = Hasp; C19 = HBMEC; C20 = HCFaa; C21 = HCPE; C22 = HEE; C23 = HEK293; C24 = Helas3; C25 = HepG2; C26 = HL60; C27 = HMEC; C28 = HMF; C29 = HPAF; C30 = HPF; C31 = HRE; C32 = HRPE; C33 = HUVEC; C34 = K562; C35 = NHEK; C36 = SAEC; C37 = SKNSHRA; C38 = WERIRB1. (PDF) [file pone.0041374.s001.pdf]

Figure S2

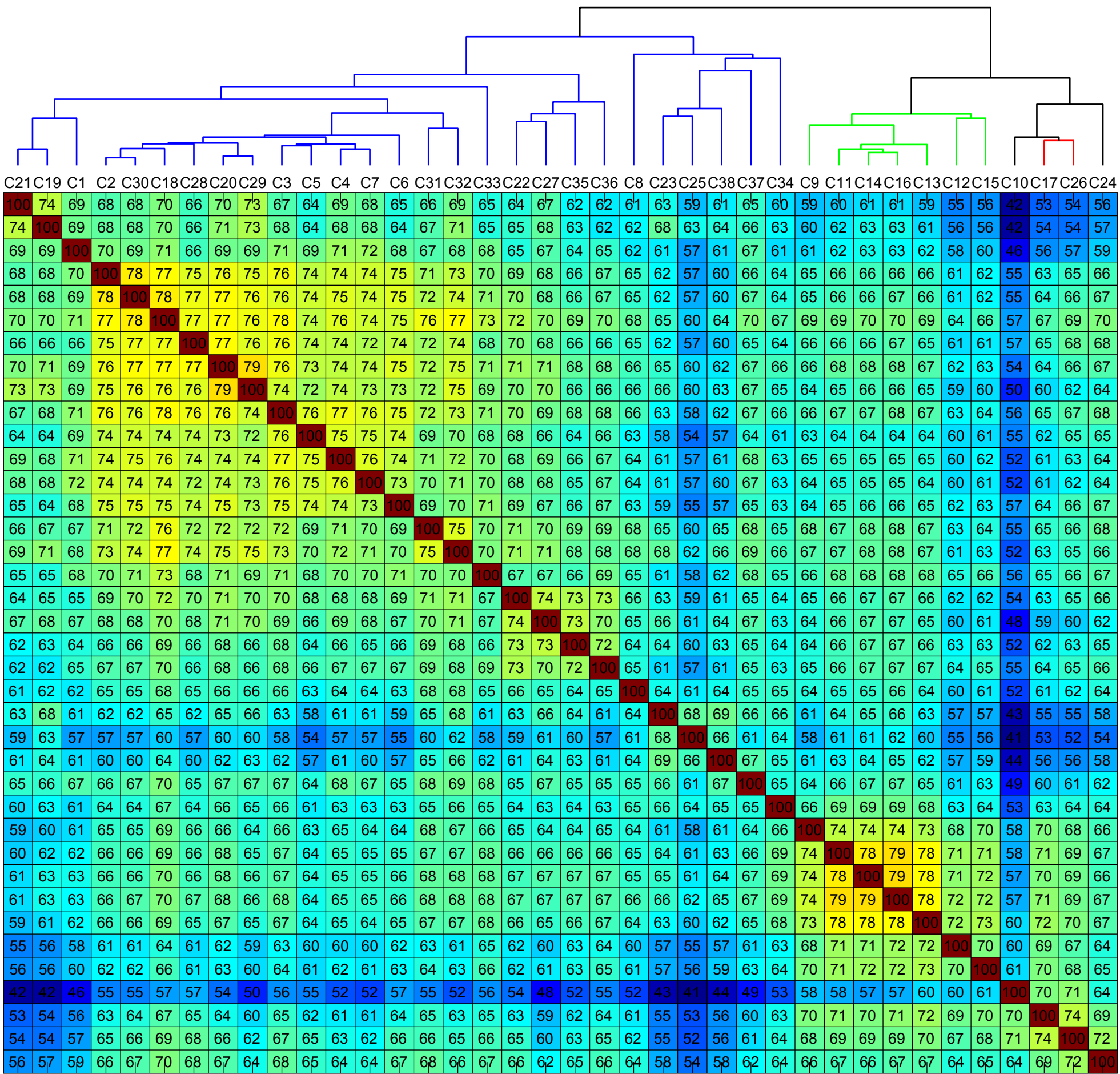

Supplement: Figure S2 — Overlap of CTCF-binding sites across 38 cell types. Heatmap representing the overlap between CTCF-binding sites across the 38 cell types examined. Cell lines were clustered based on their CTCF-binding site using Ward's hierarchical clustering with Cluster 3.0. (PDF) [file pone.0041374.s002.pdf]

13,598 CTCF sites

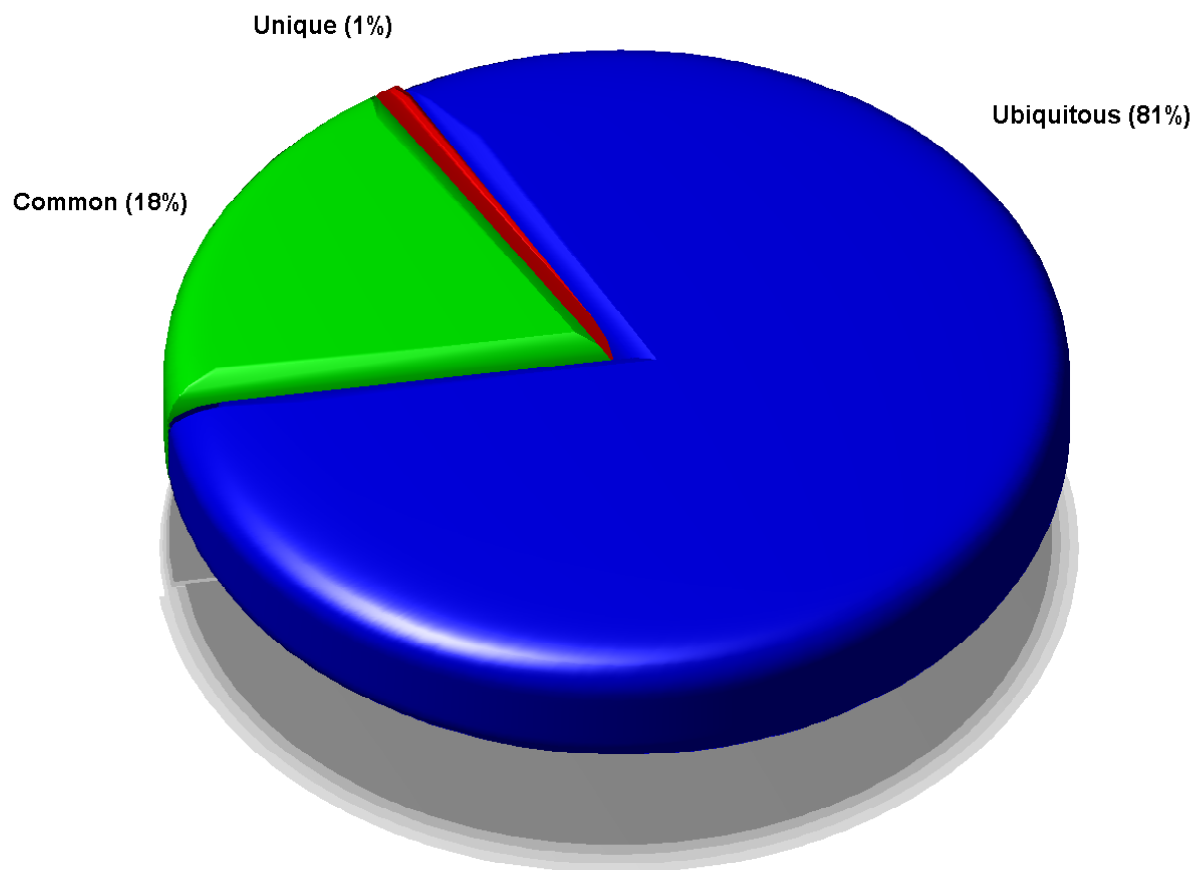

13,598 CTCF sites

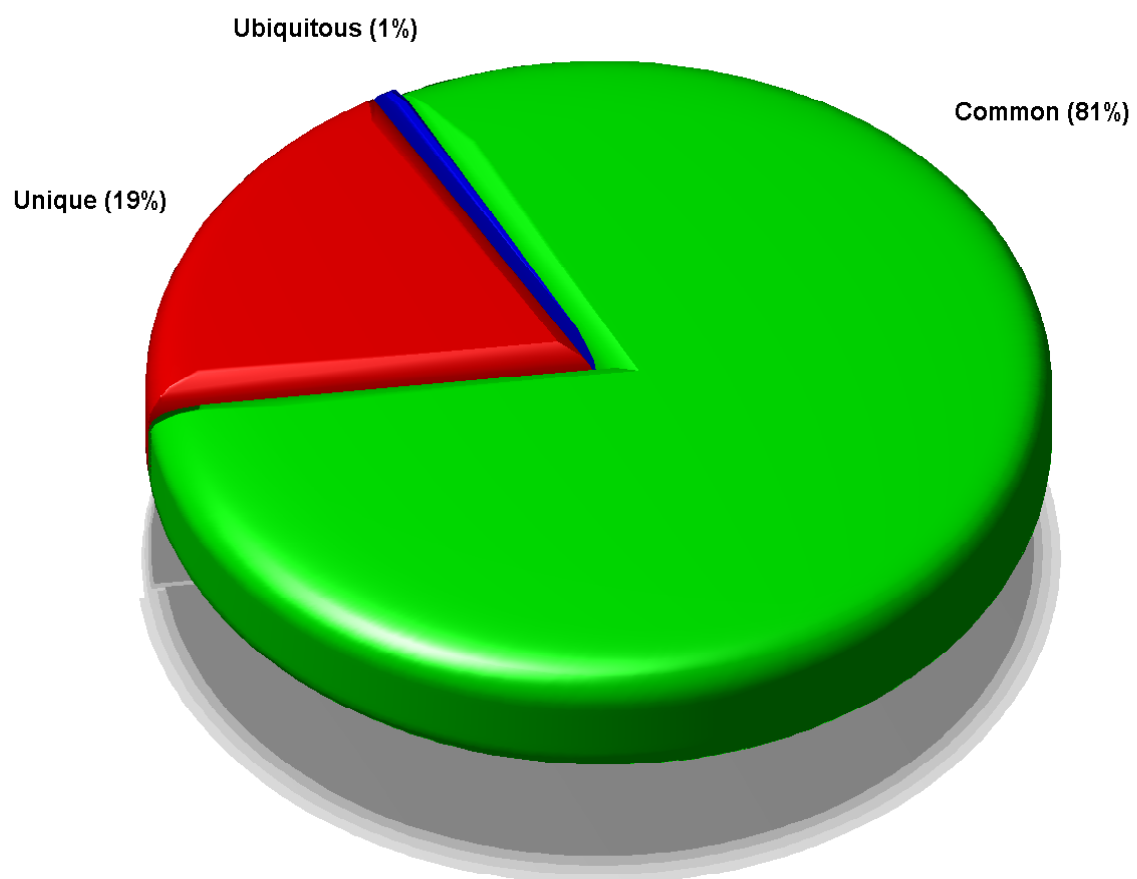

Supplement: Figure S3 — Genome-wide distribution of strongest and weakest scoring CTCF-binding sites relative to cell type. The proportion of CTCF-binding site types in the K562 cell line among the strongest scoring (top 20%), and weakest scoring (bottom 20%) CTCF-binding sites are shown. In the strongest scoring CTCF-binding sites, 81% were found to be ubiquitous and almost none (0.7%) were cell type-specific. In contrast, in the weakest scoring CTCF-binding sites, 19% were cell type-specific and almost none (0.6%) were ubiquitous. (PDF) [file pone.0041374.s003.pdf]

Figure S4

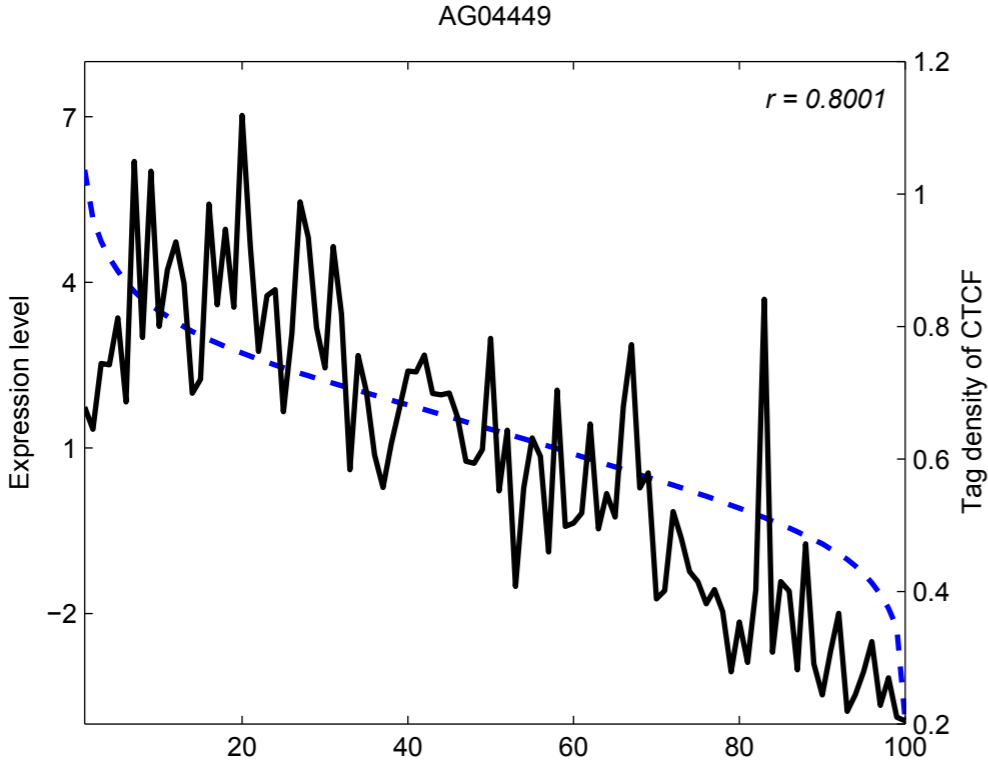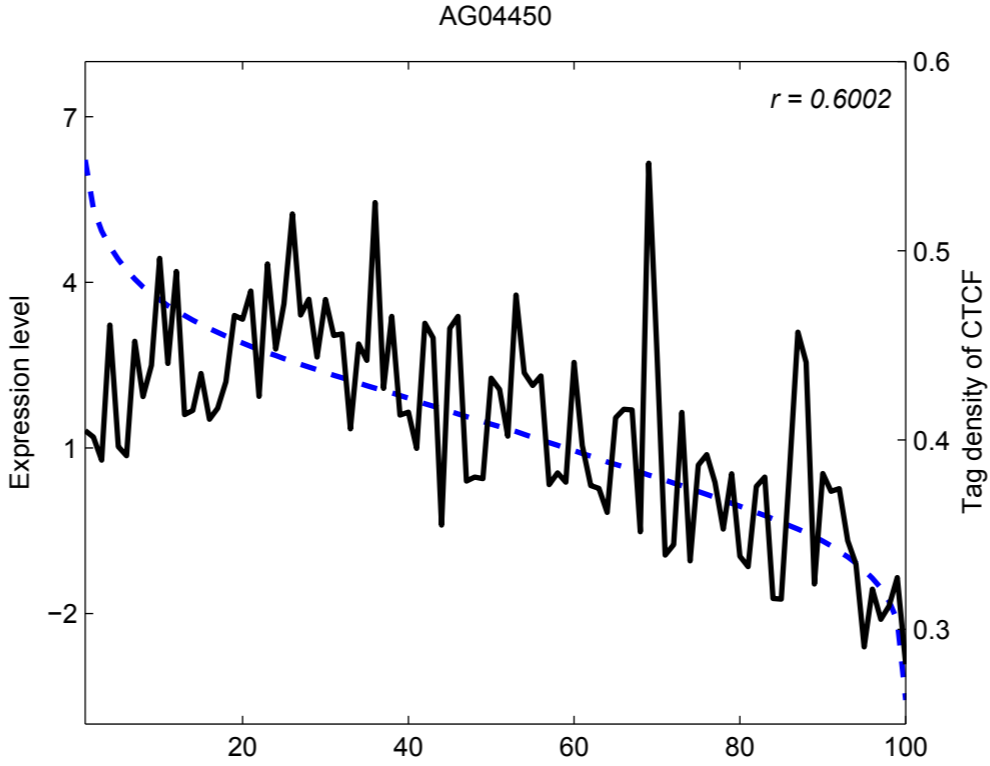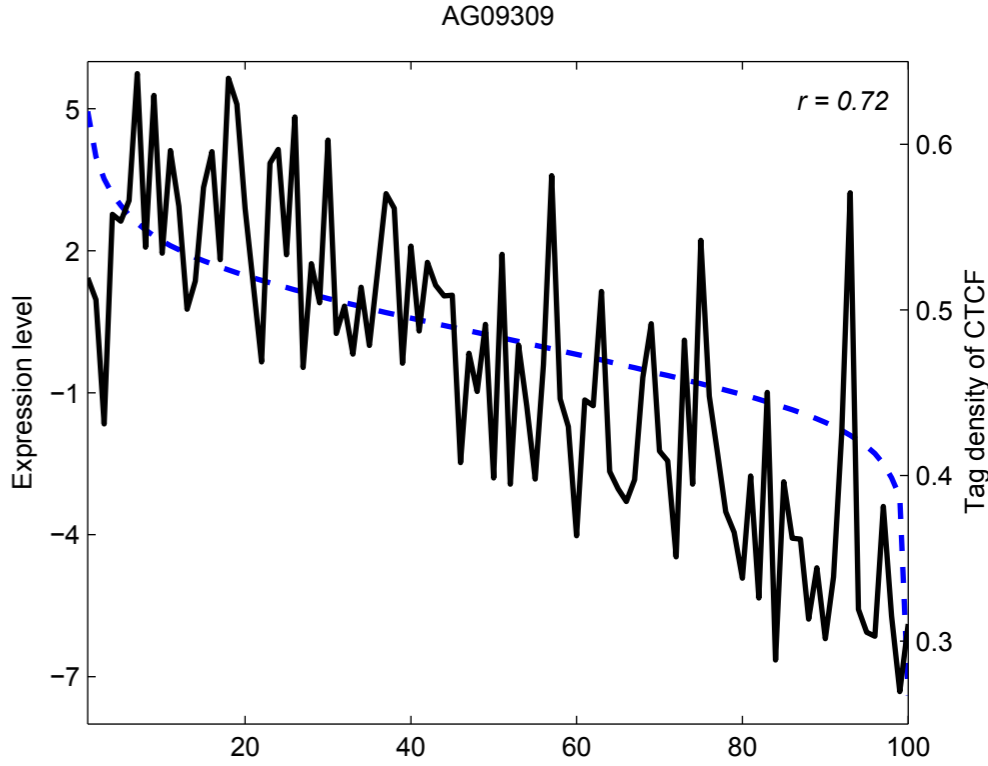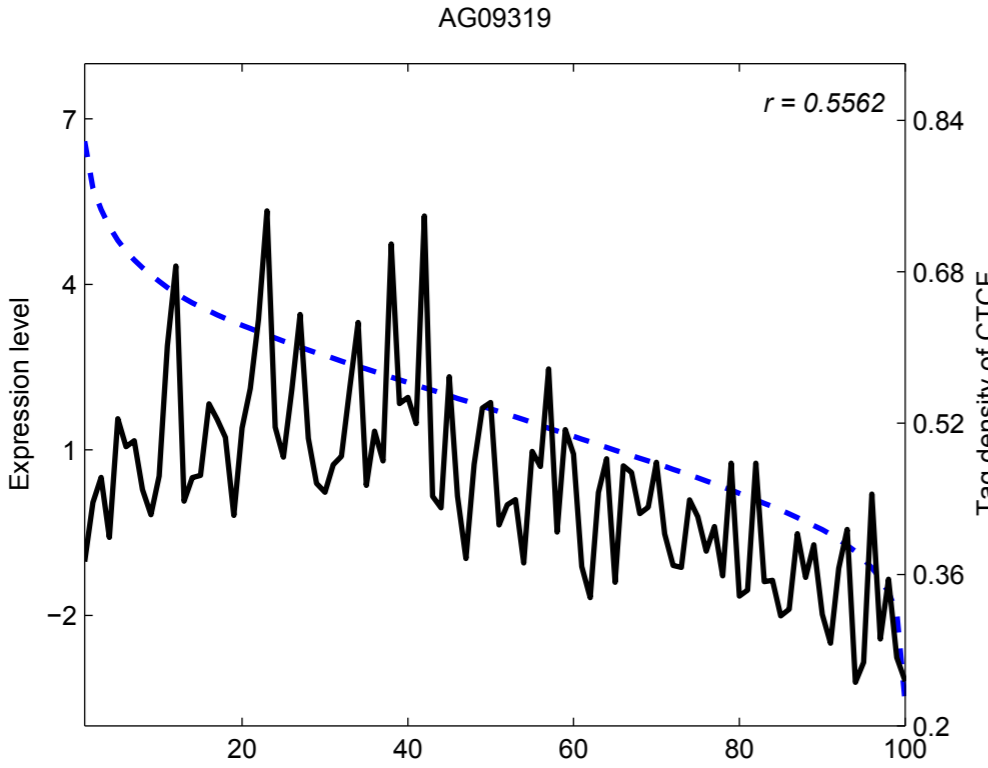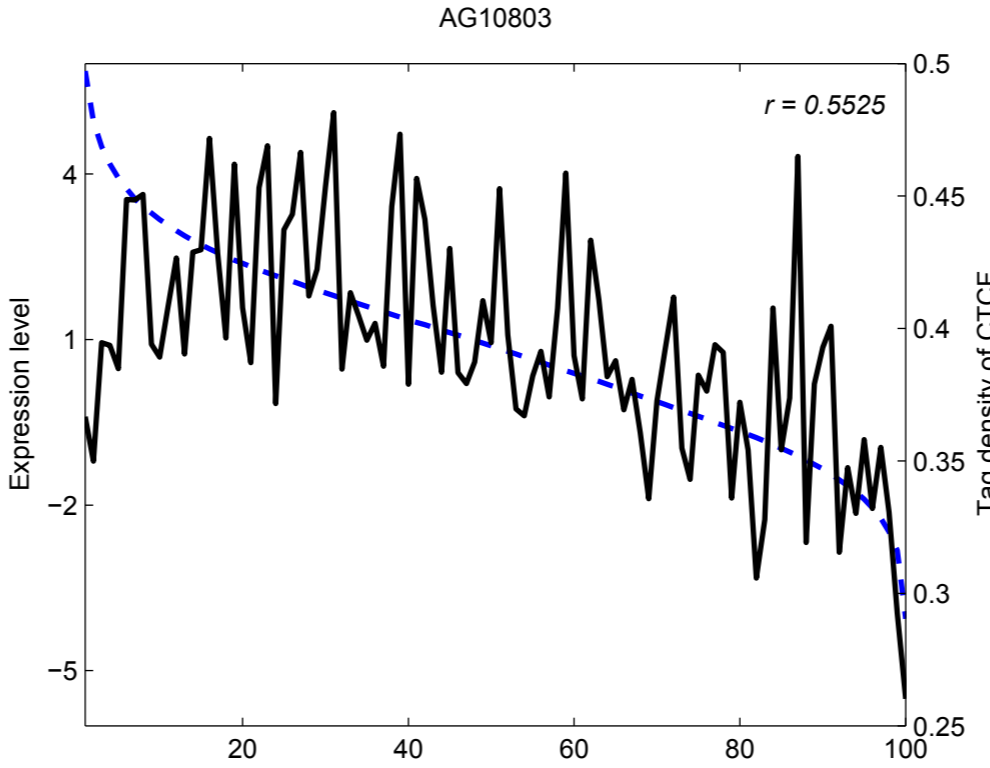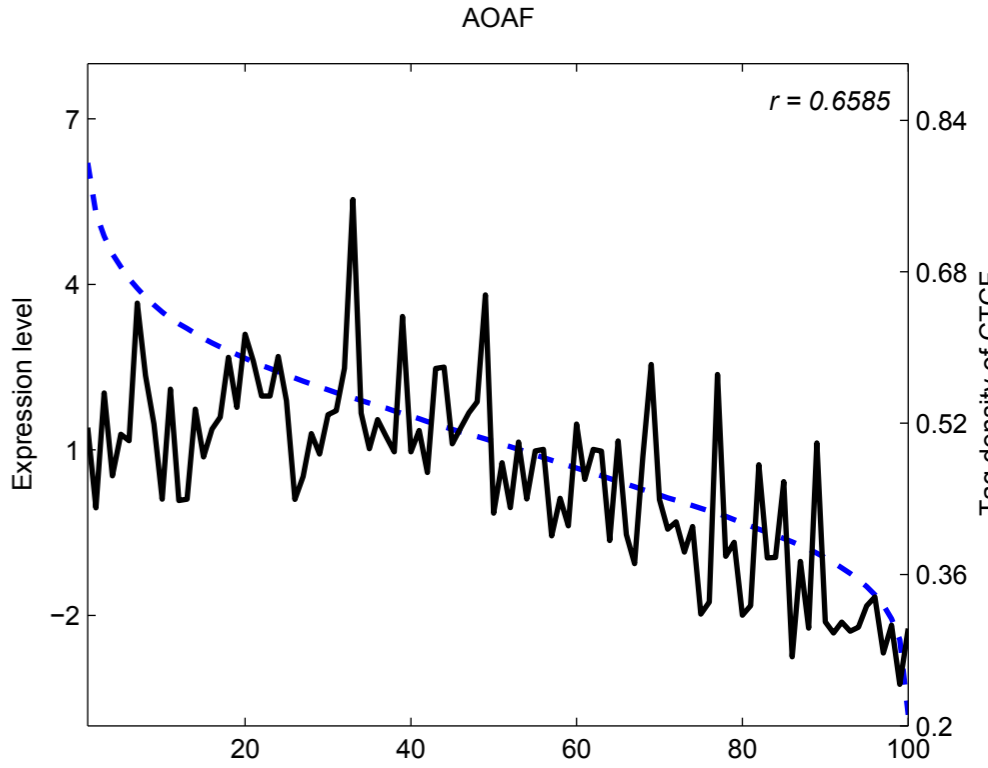

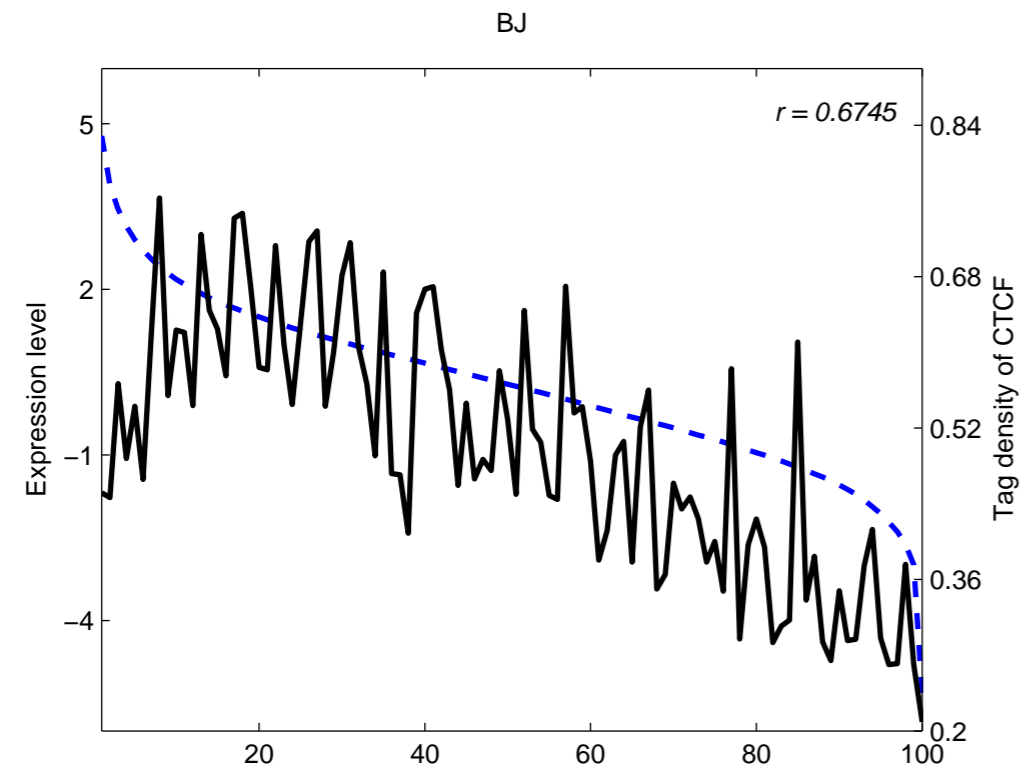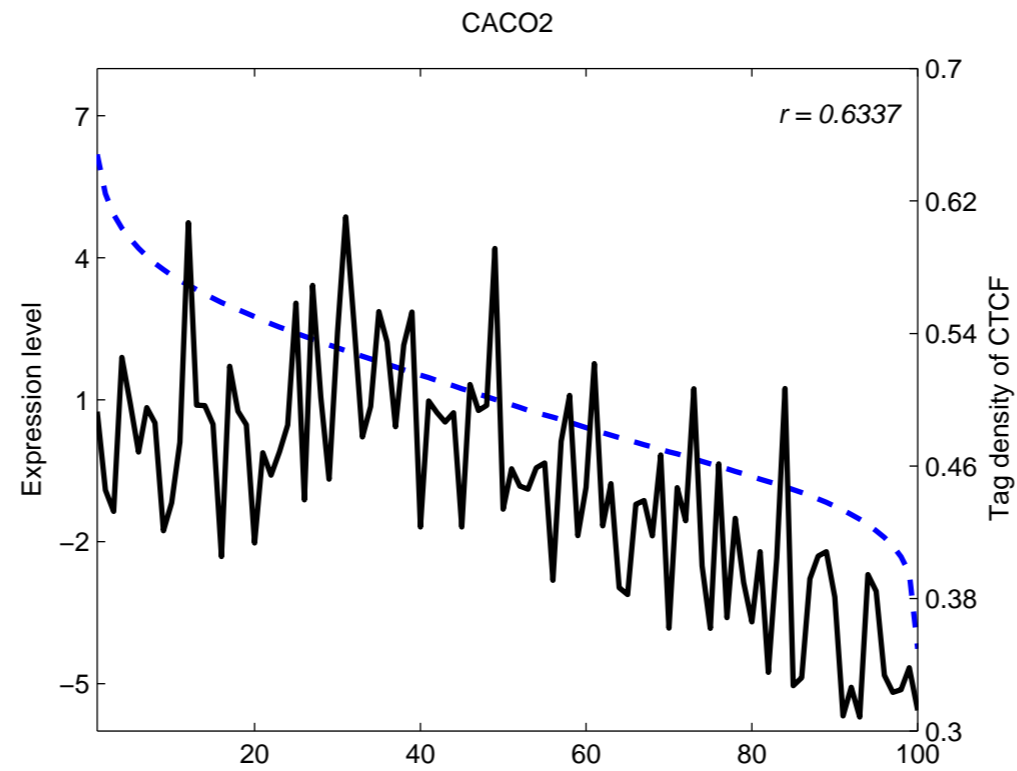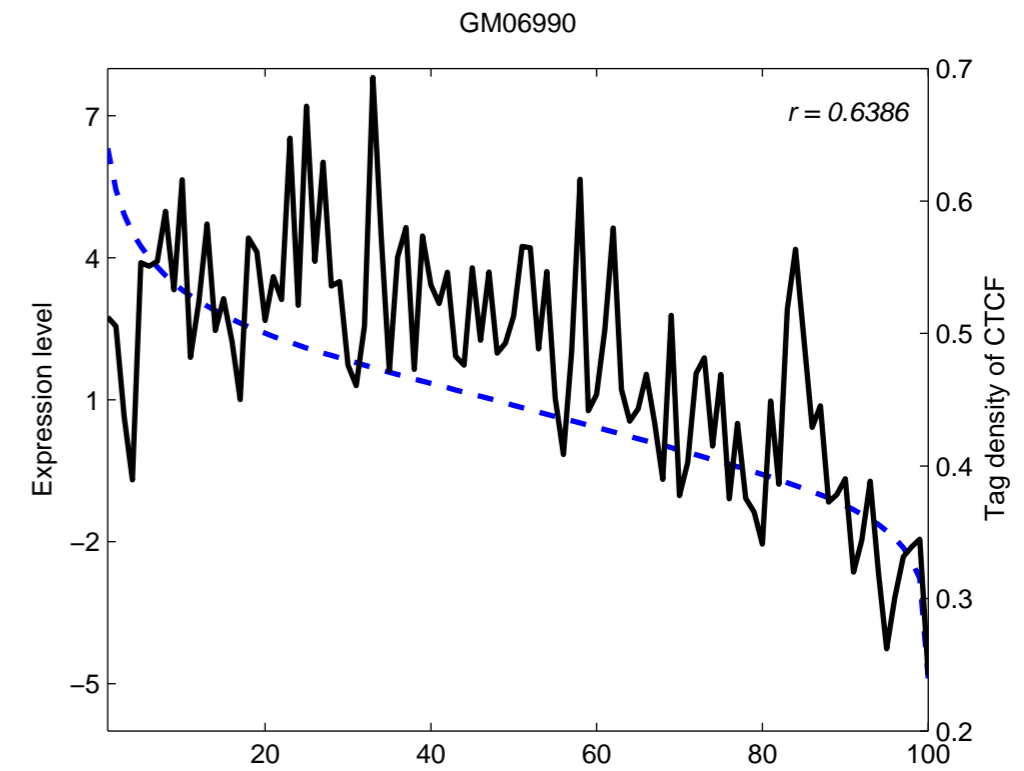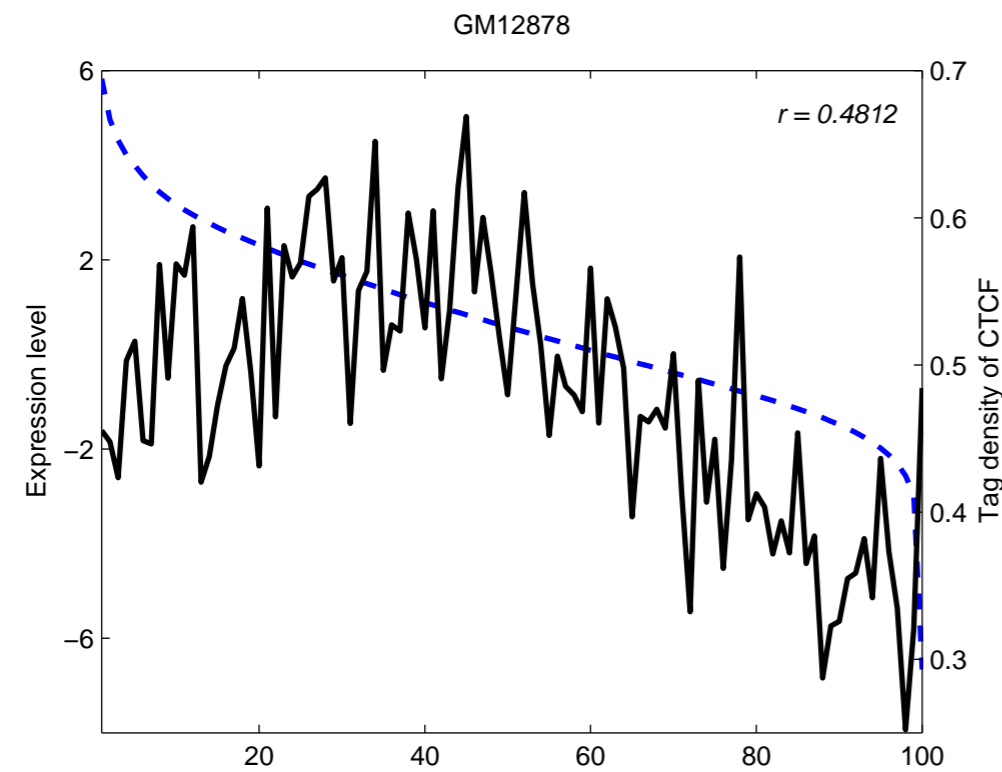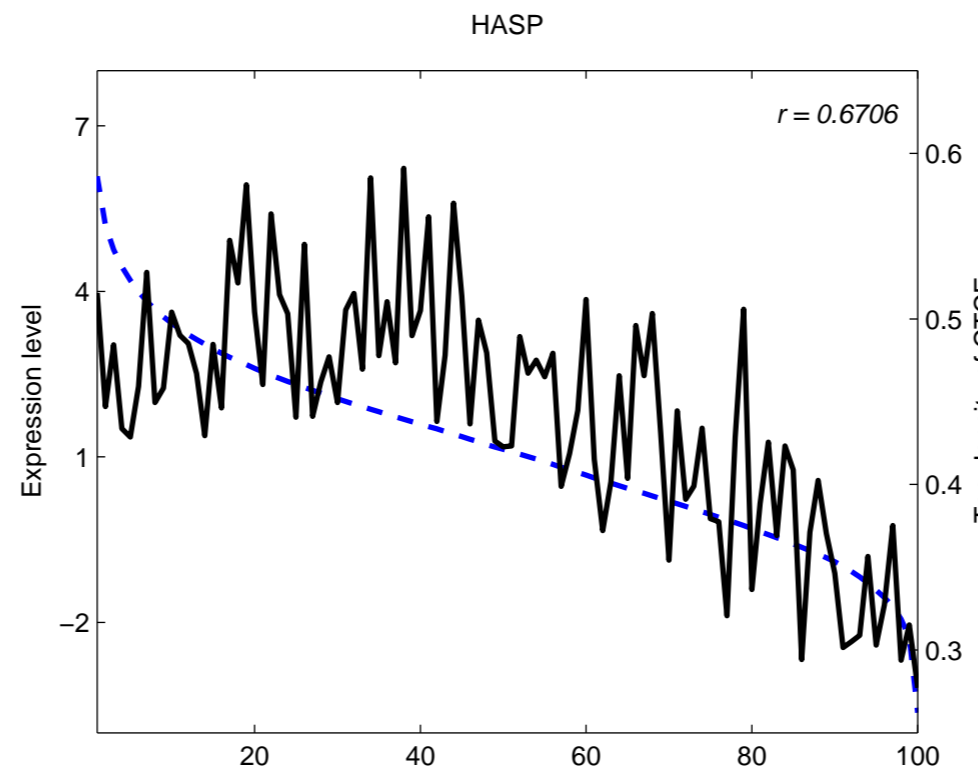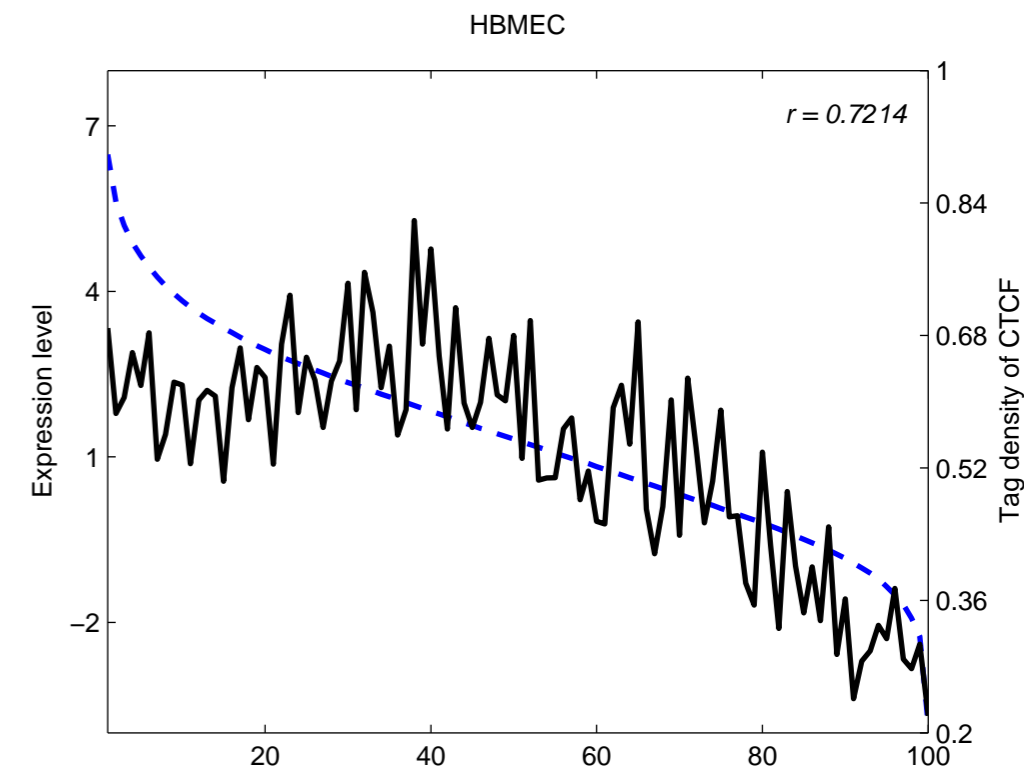

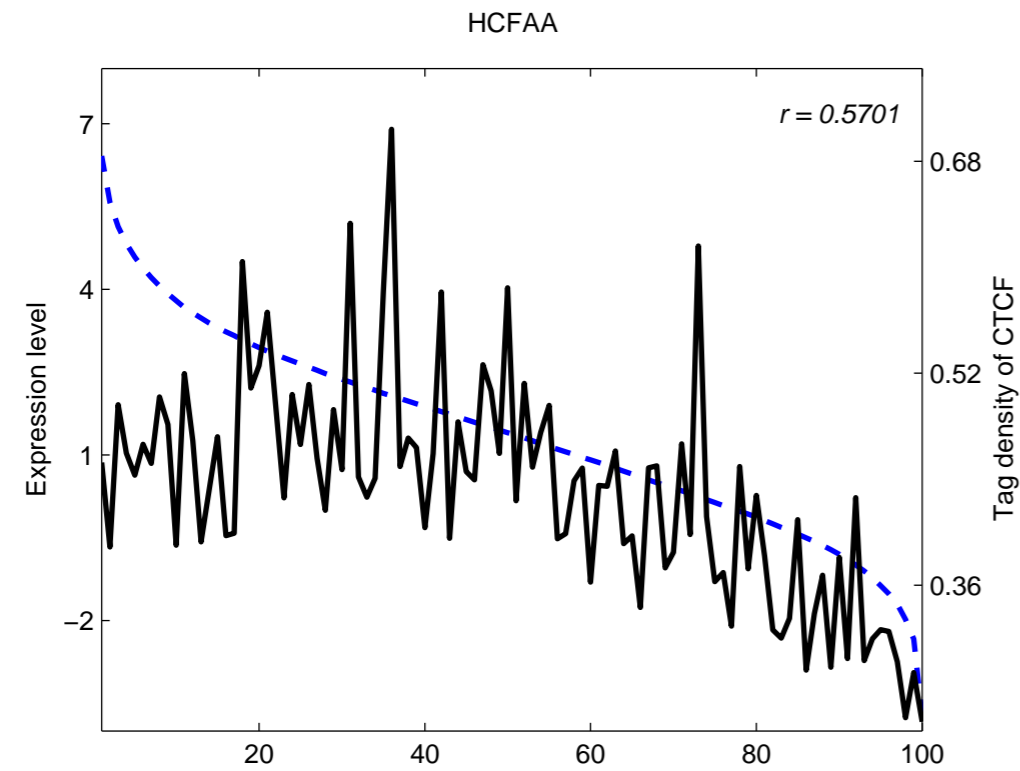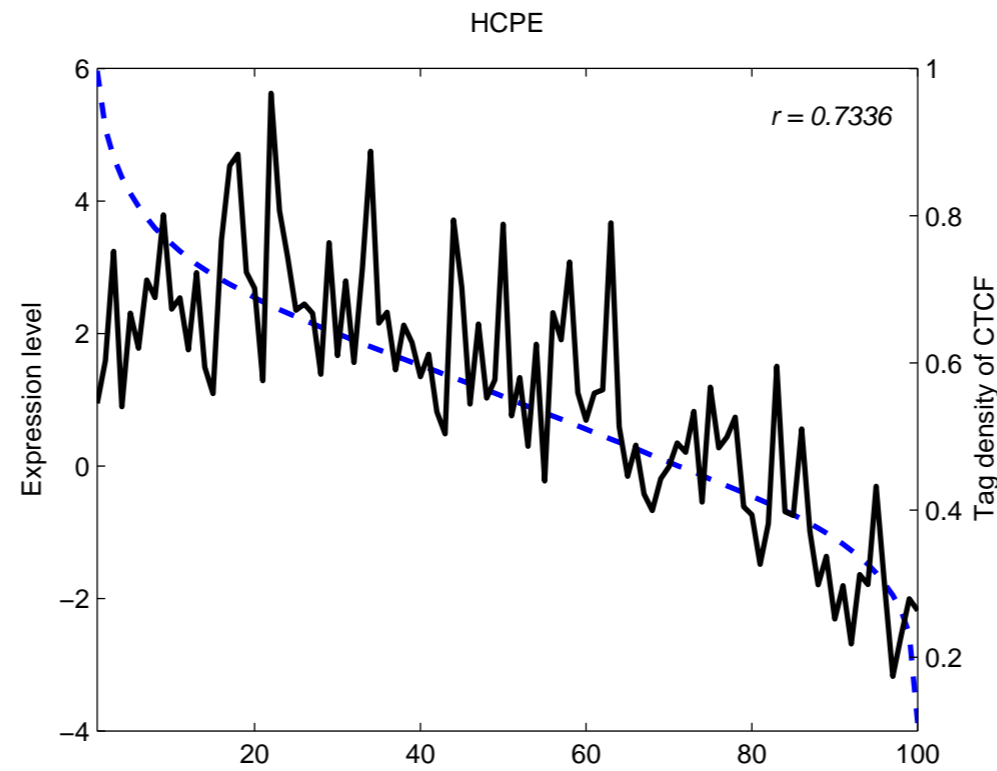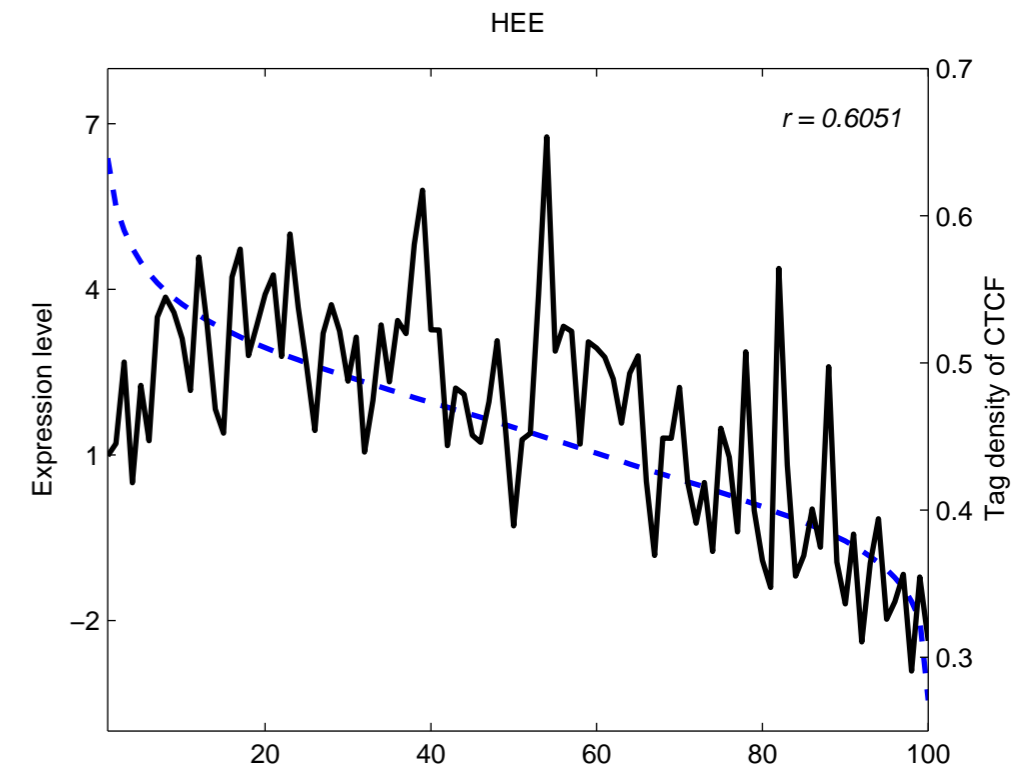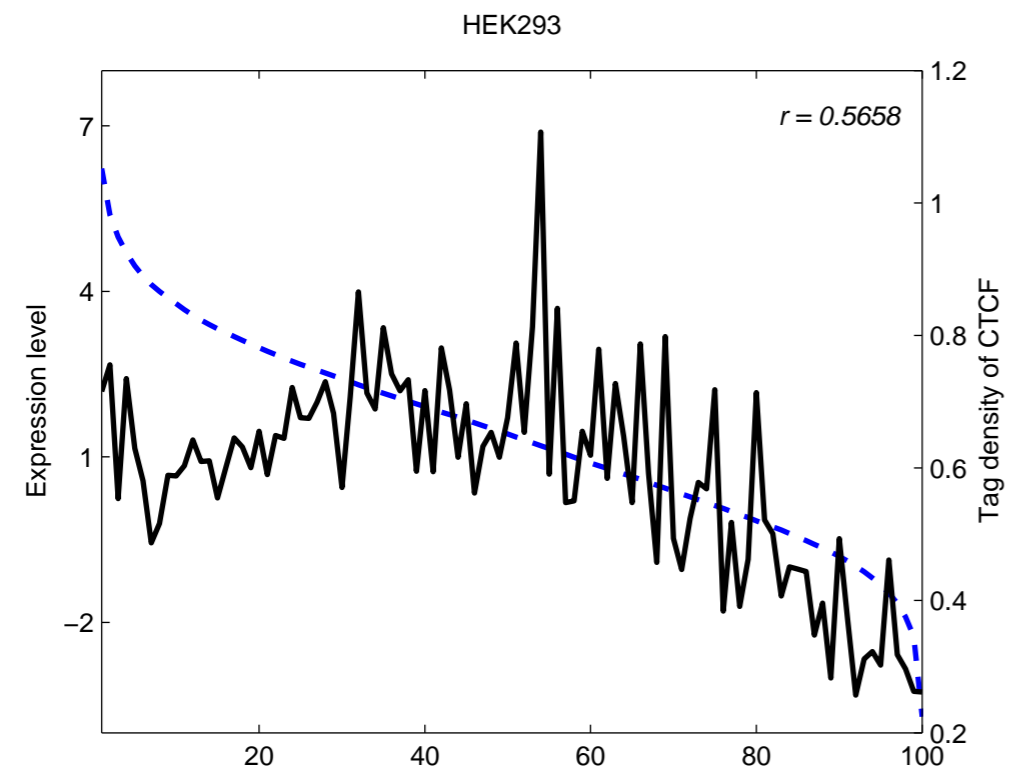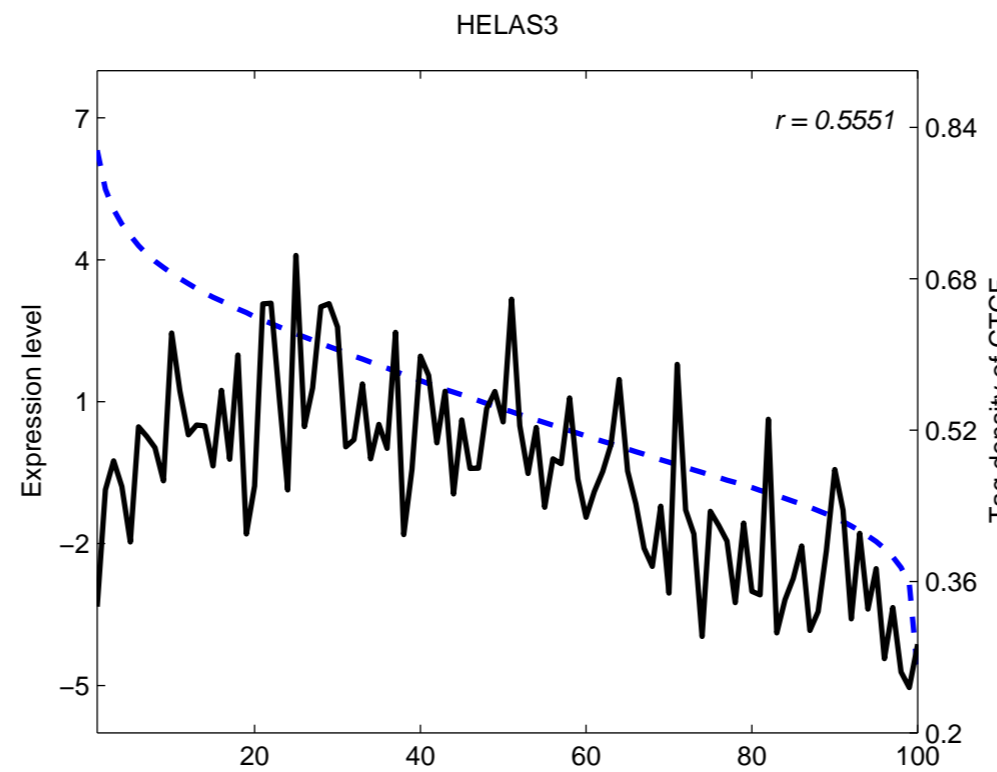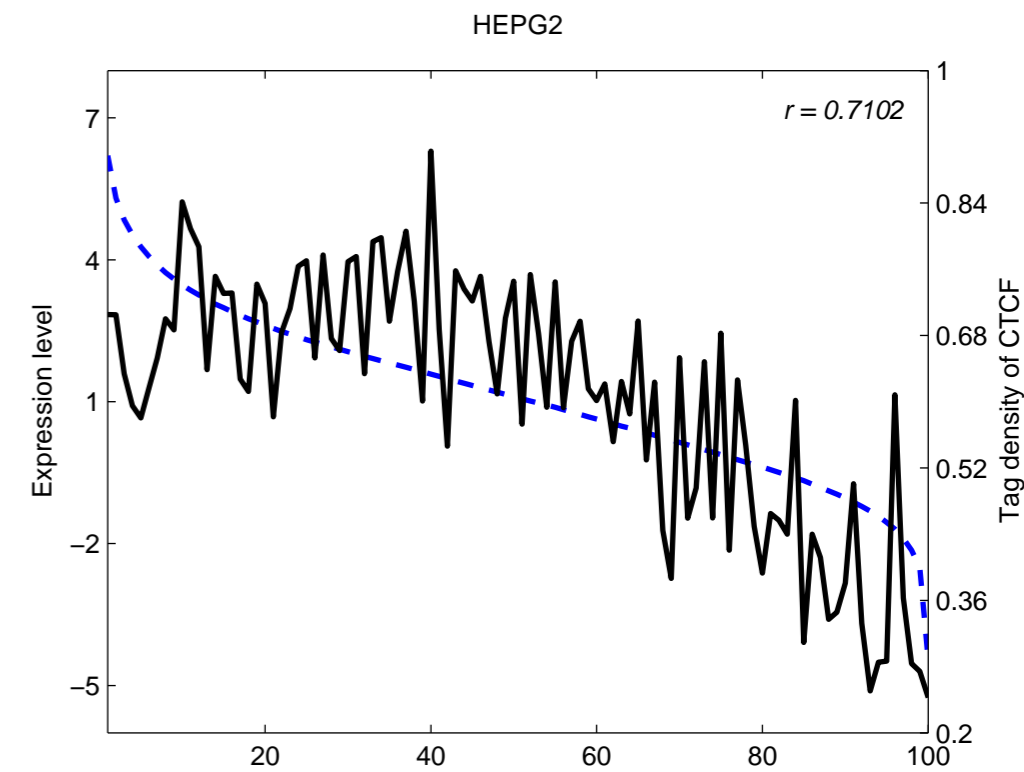

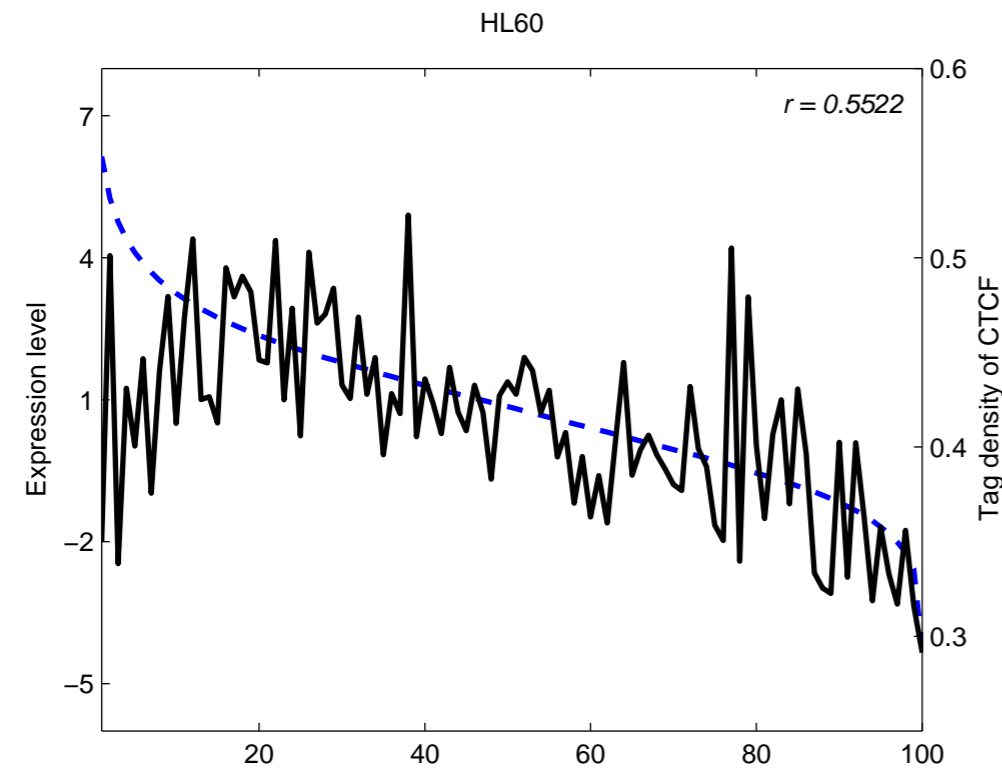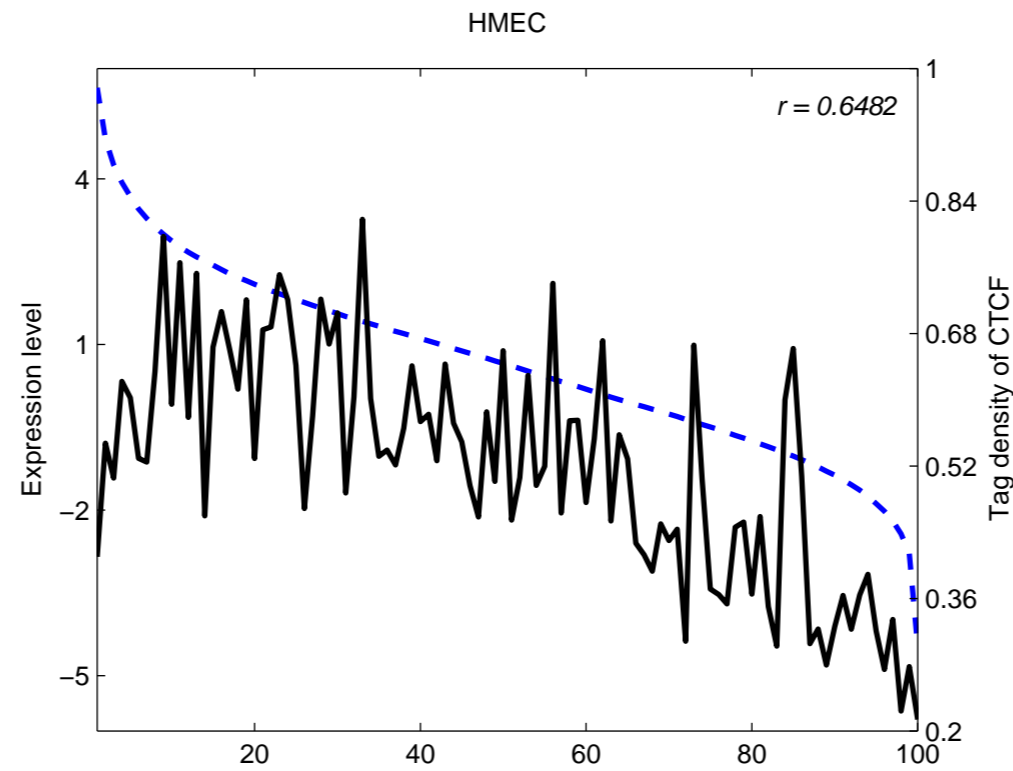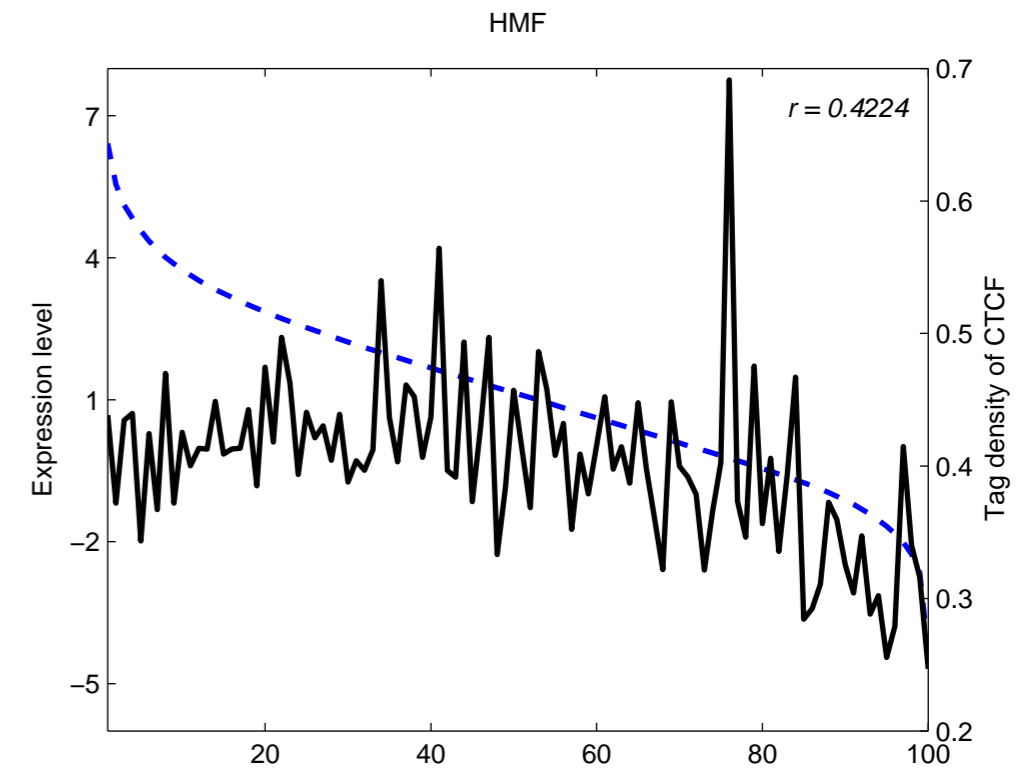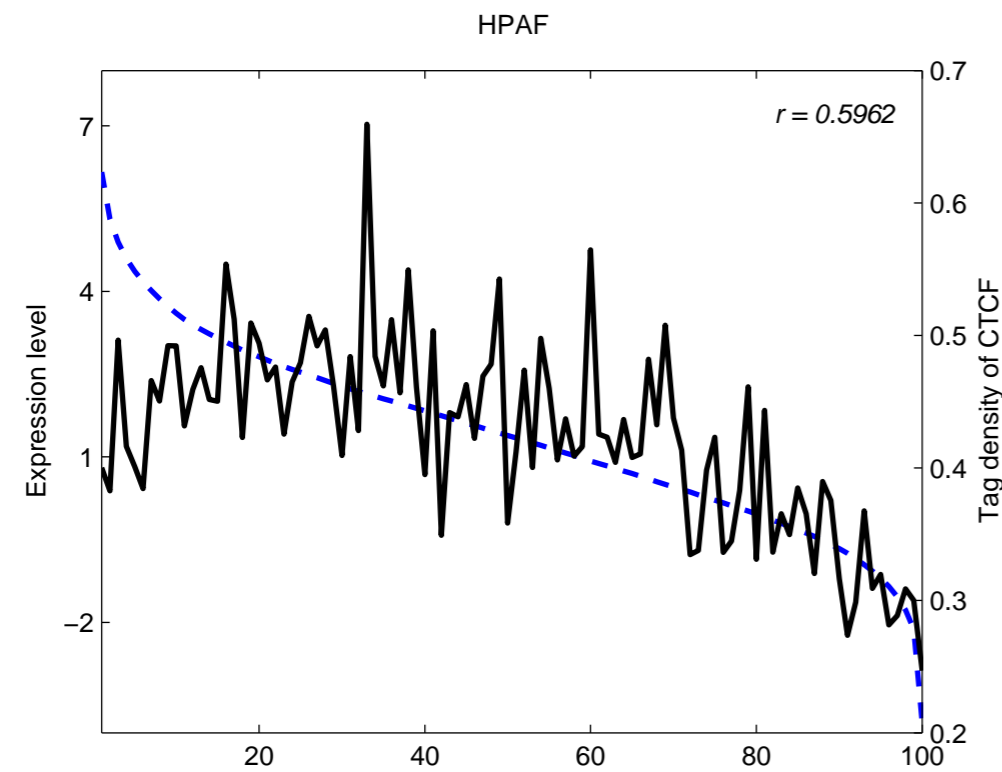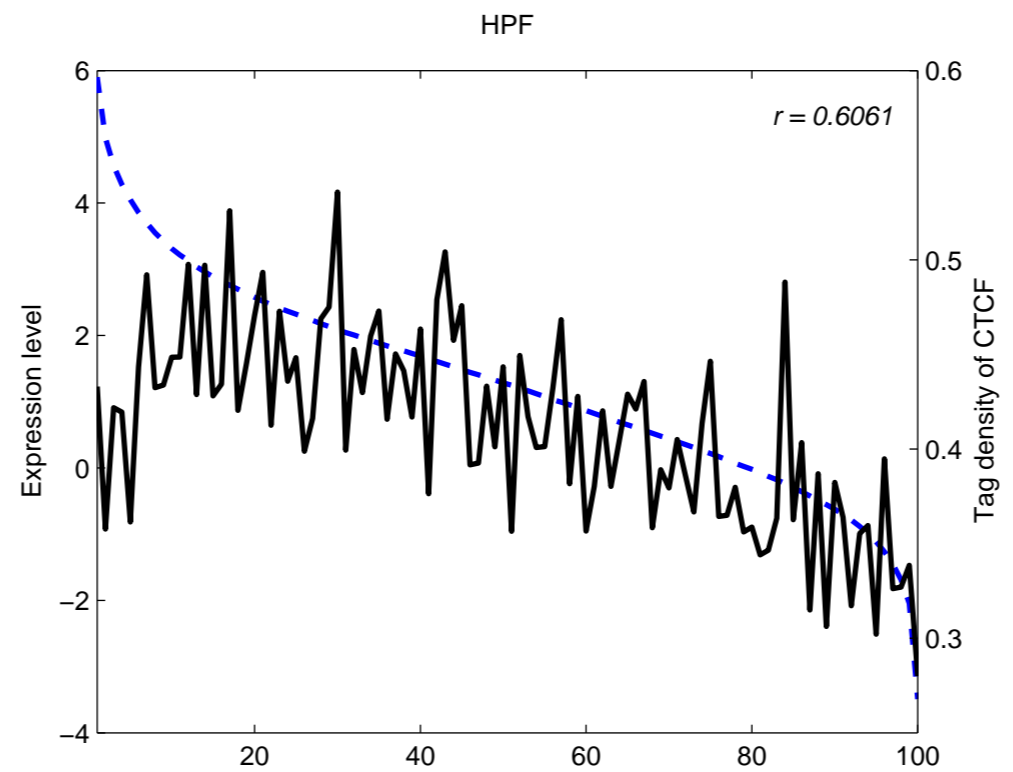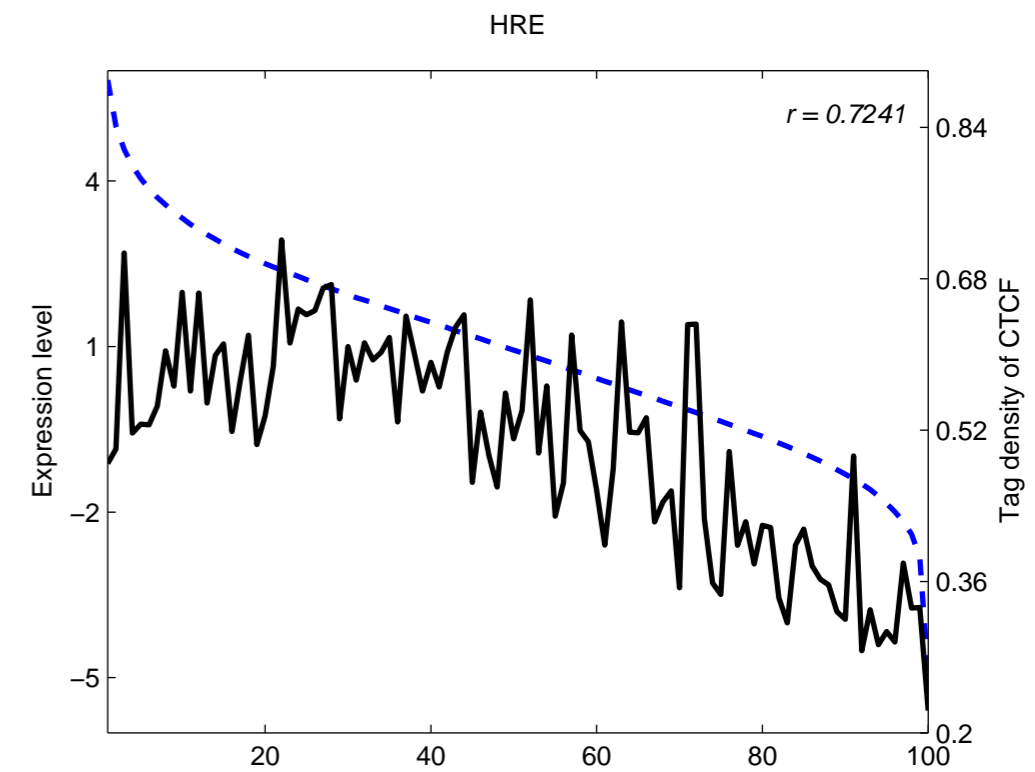

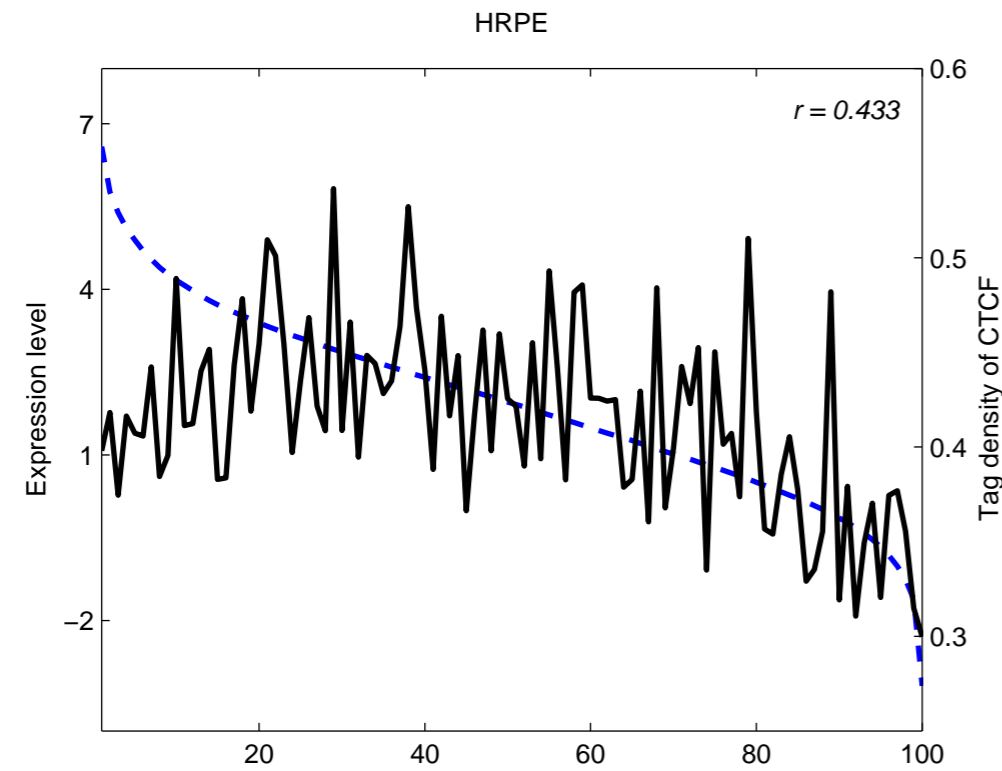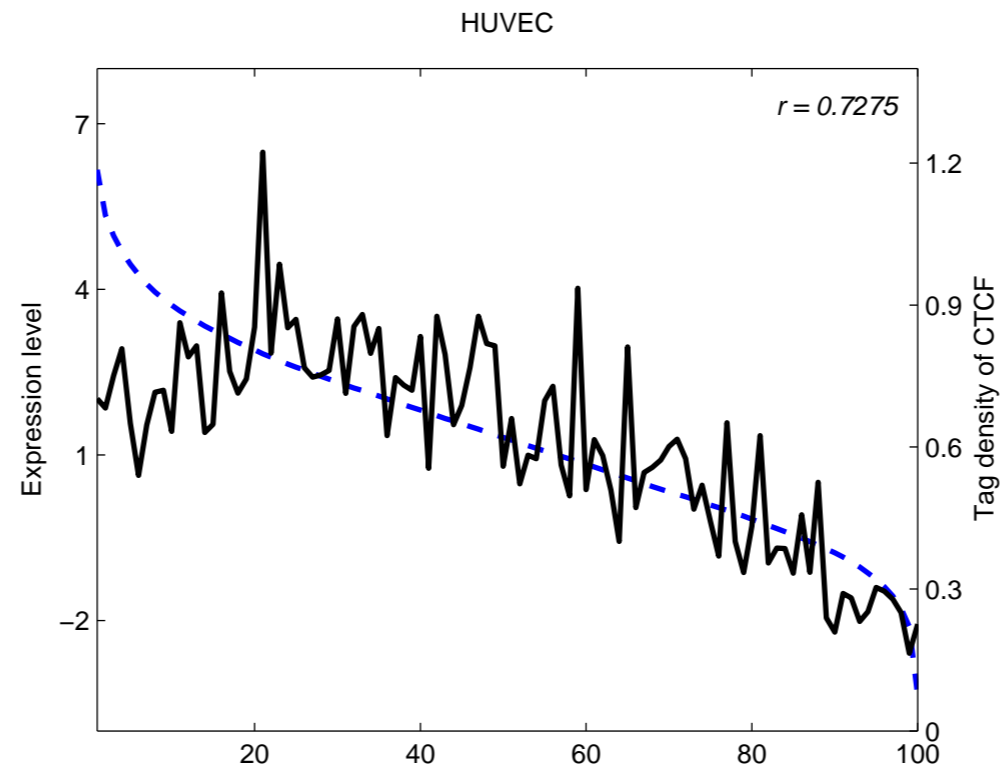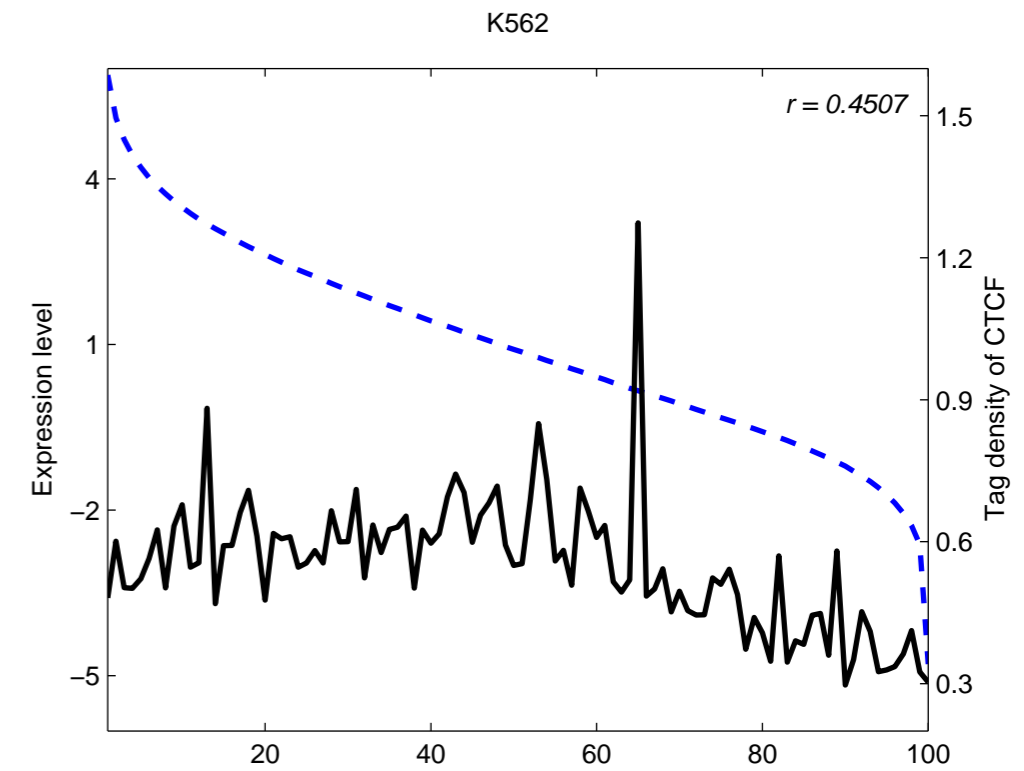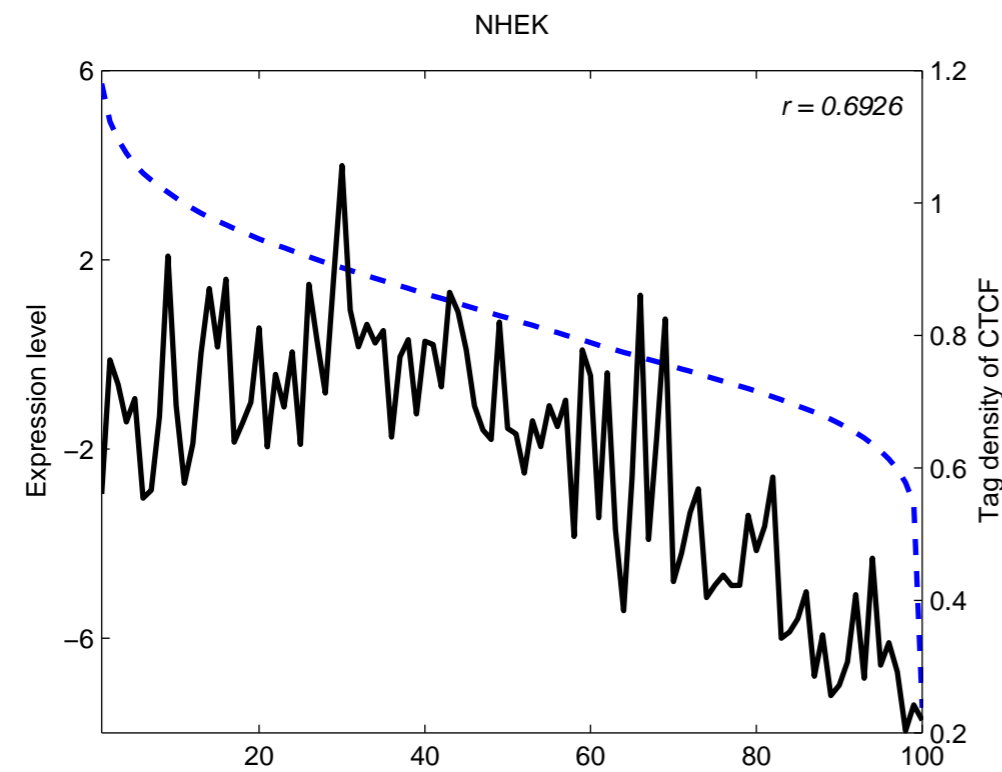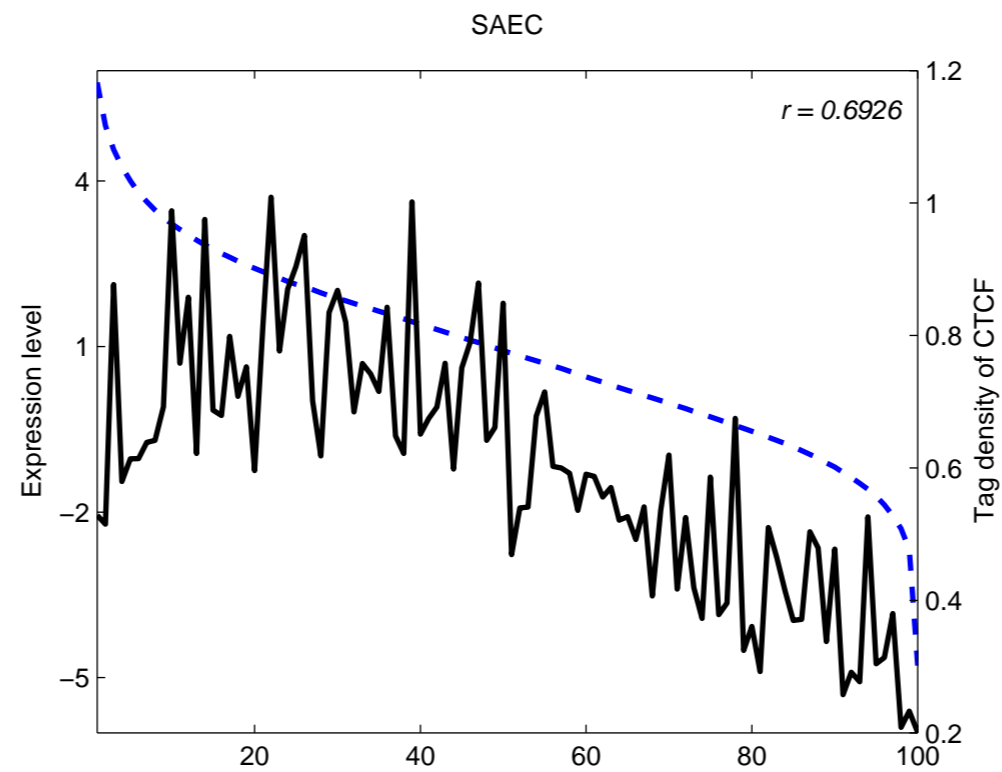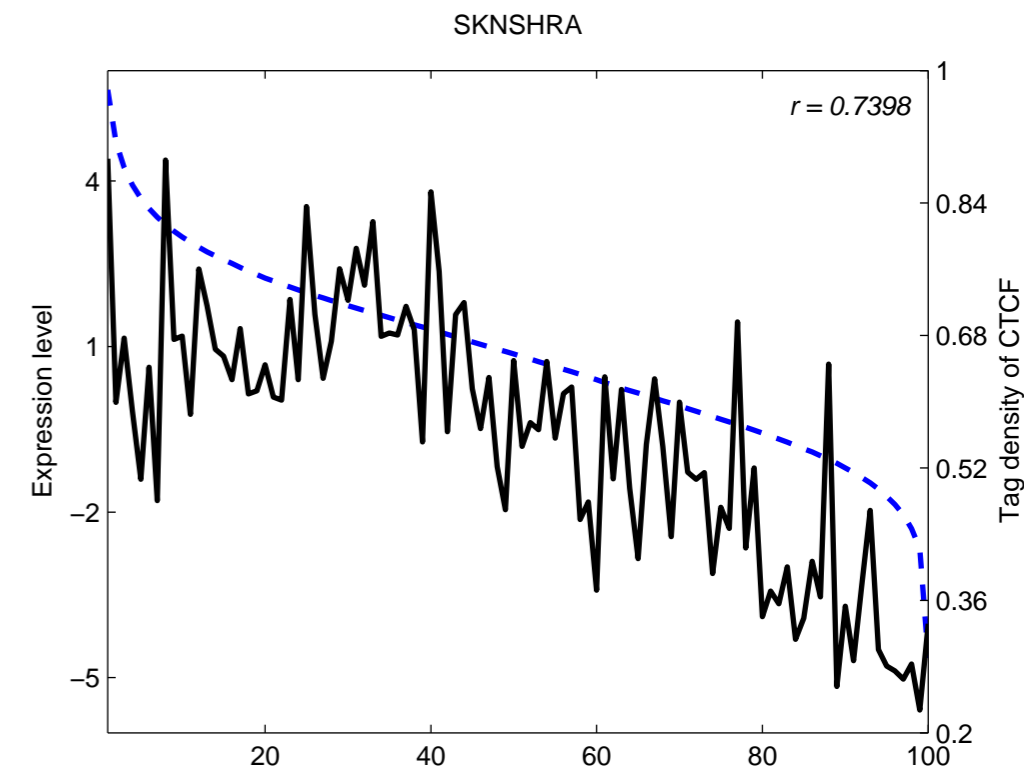

Supplement: Figure S4 — Scatter correlation between CTCF-binding sites and gene expression across cell types. CTCF-binding sites were grouped into 100 sets (dot) based on the gene expression levels (from high to low, left to right on the x-axis). The average tag densities of CTCF and the average gene expression levels were calculated for each group and plotted according to the average gene expression level (left y-axis) and the average tag densities of CTCF (right y-axis). (PDF) [file pone.0041374.s004.pdf]

(A)

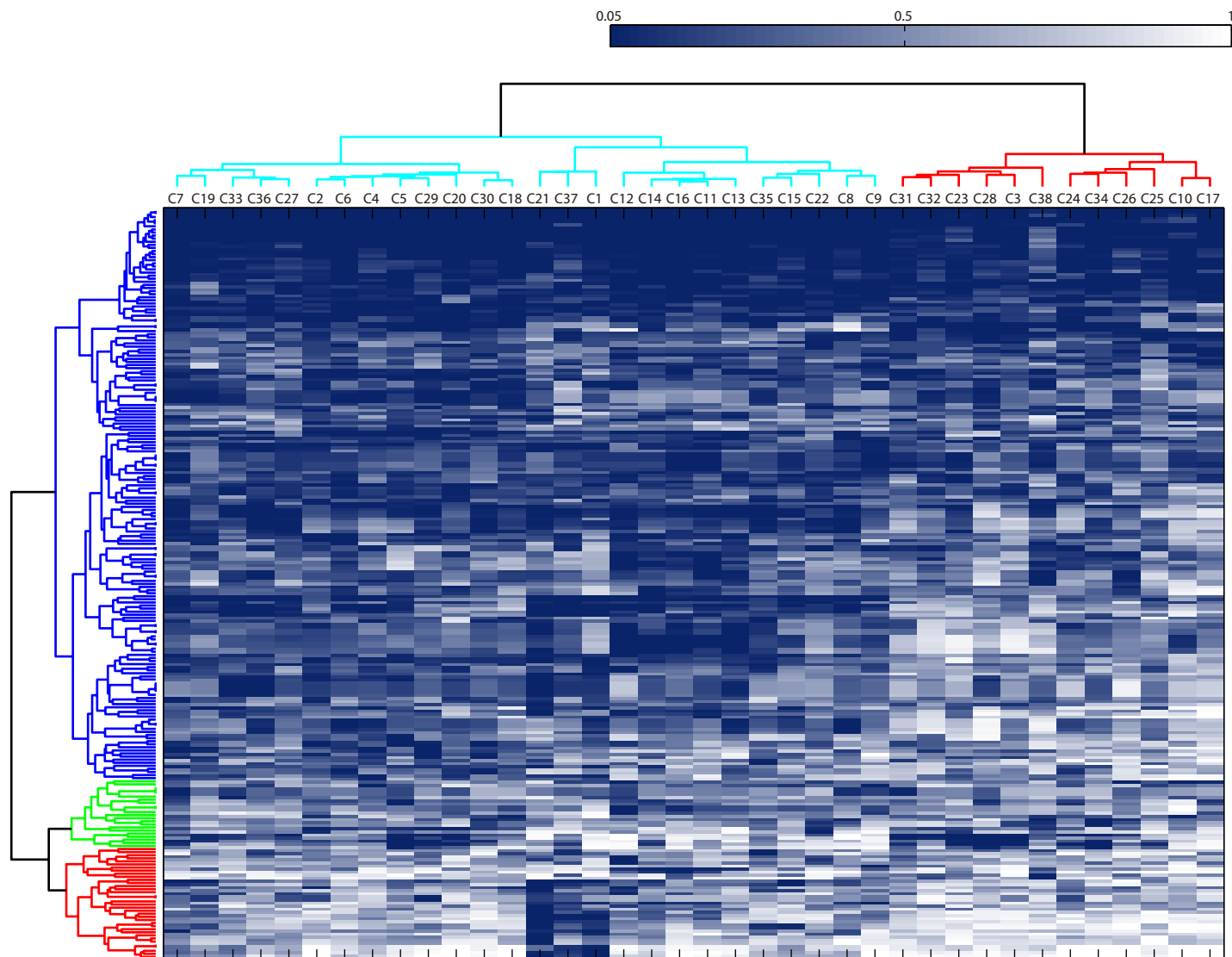

(B)

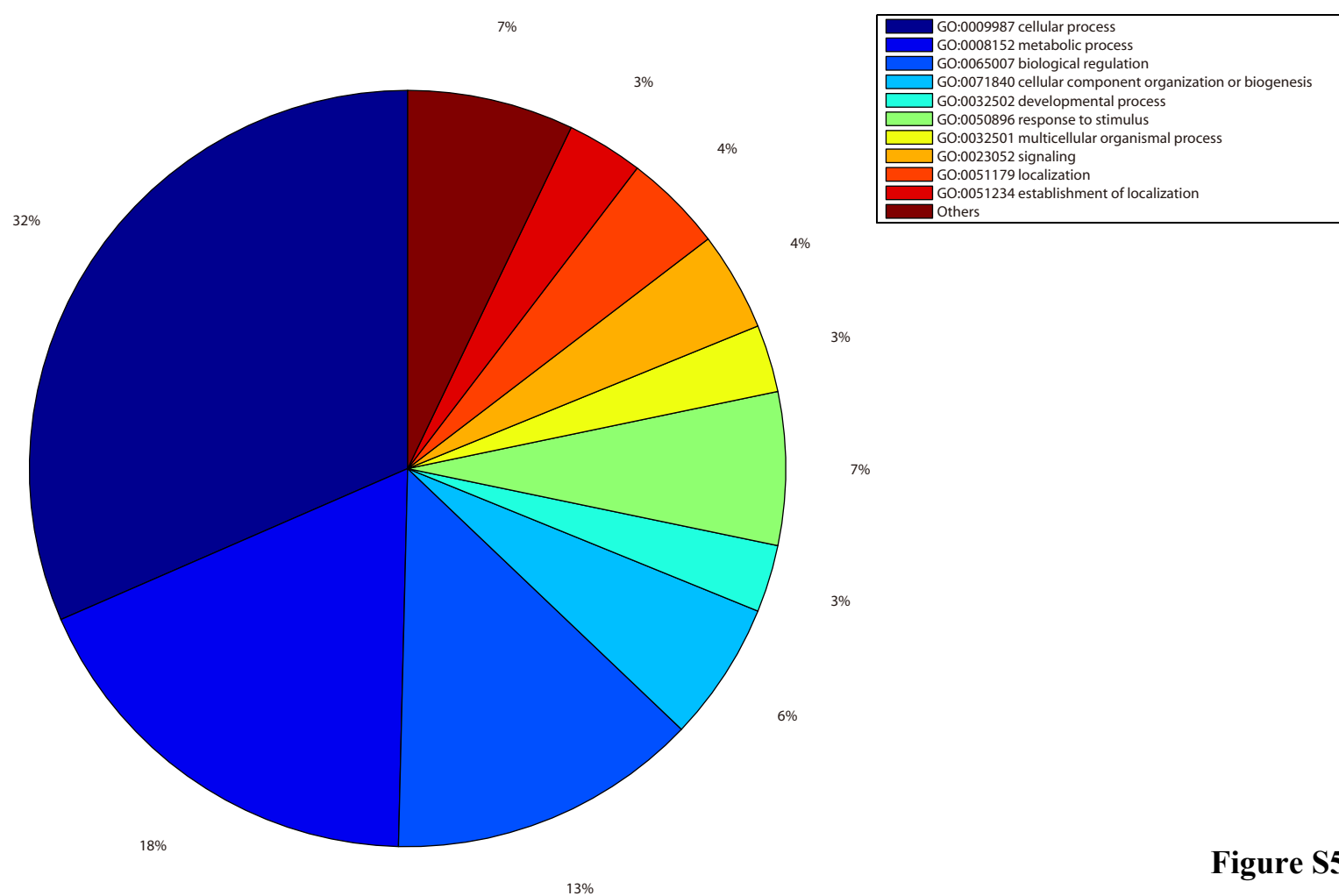

Figure S5

Supplement: Figure S5 — GO analysis of proximal CTCF-binding sites across 38 cell types. (A) Clustering of 38 cell types based on common GO nodes. This resulted in a list of 243 common GO nodes. Hierarchical clustering of both the cell types and the common GO nodes was performed using the calculated EASE scores. The relationship between the color intensity and EASE score is illustrated by the color bar. Gray indicates that an EASE score was not calculated for that GO node. The cell type is denoted by the letter and number combination at the top of every column. C1–C38 = CTCF-binding sites of 38 cell types, U = ubiquitous CTCF-binding site. (B) Summary of biological processes regulated by genes related to the proximal CTCF-binding sites across 38 cell types. Annotations were obtained from the Gene Ontology database. (PDF) [file pone.0041374.s005.pdf]

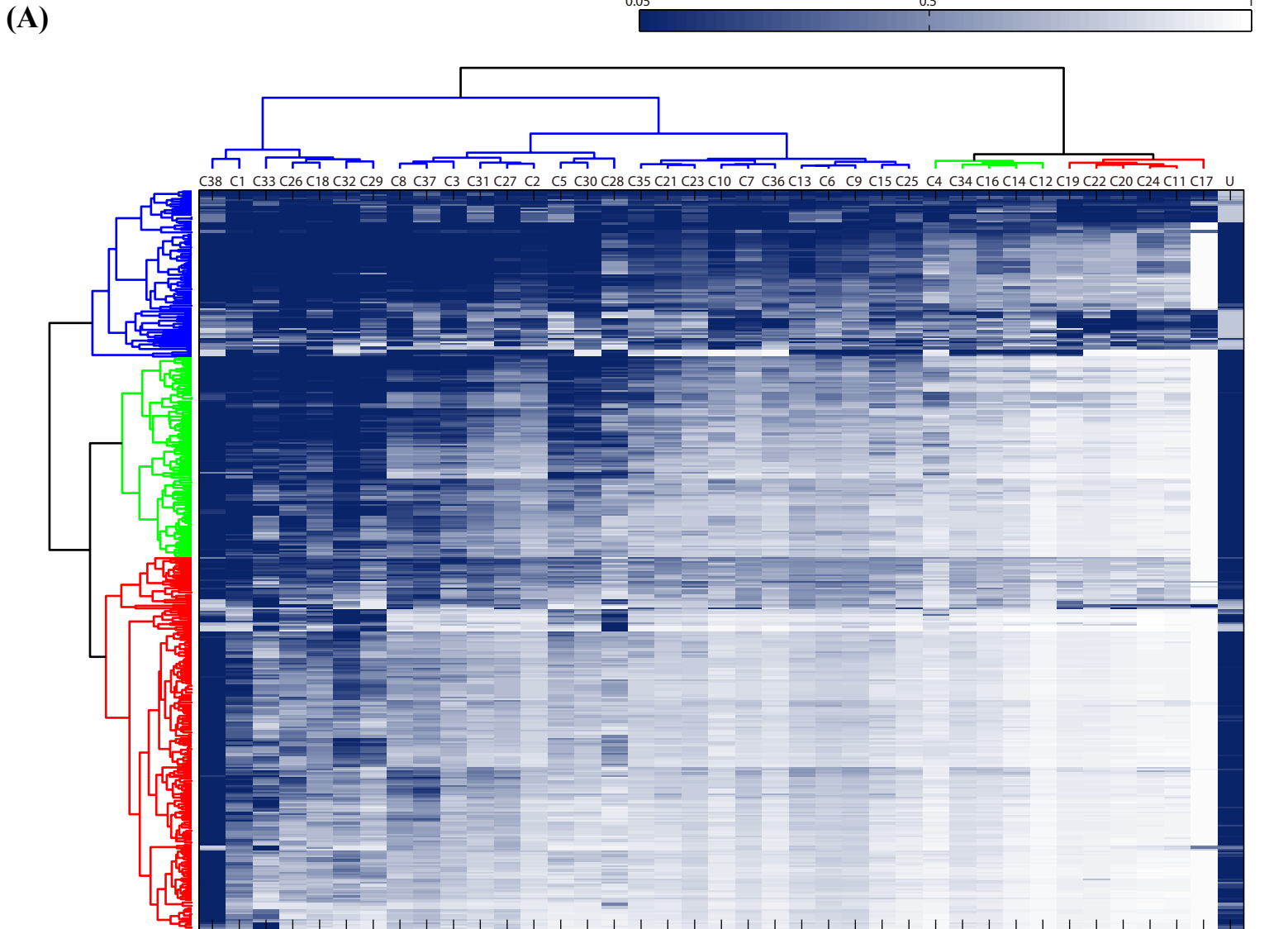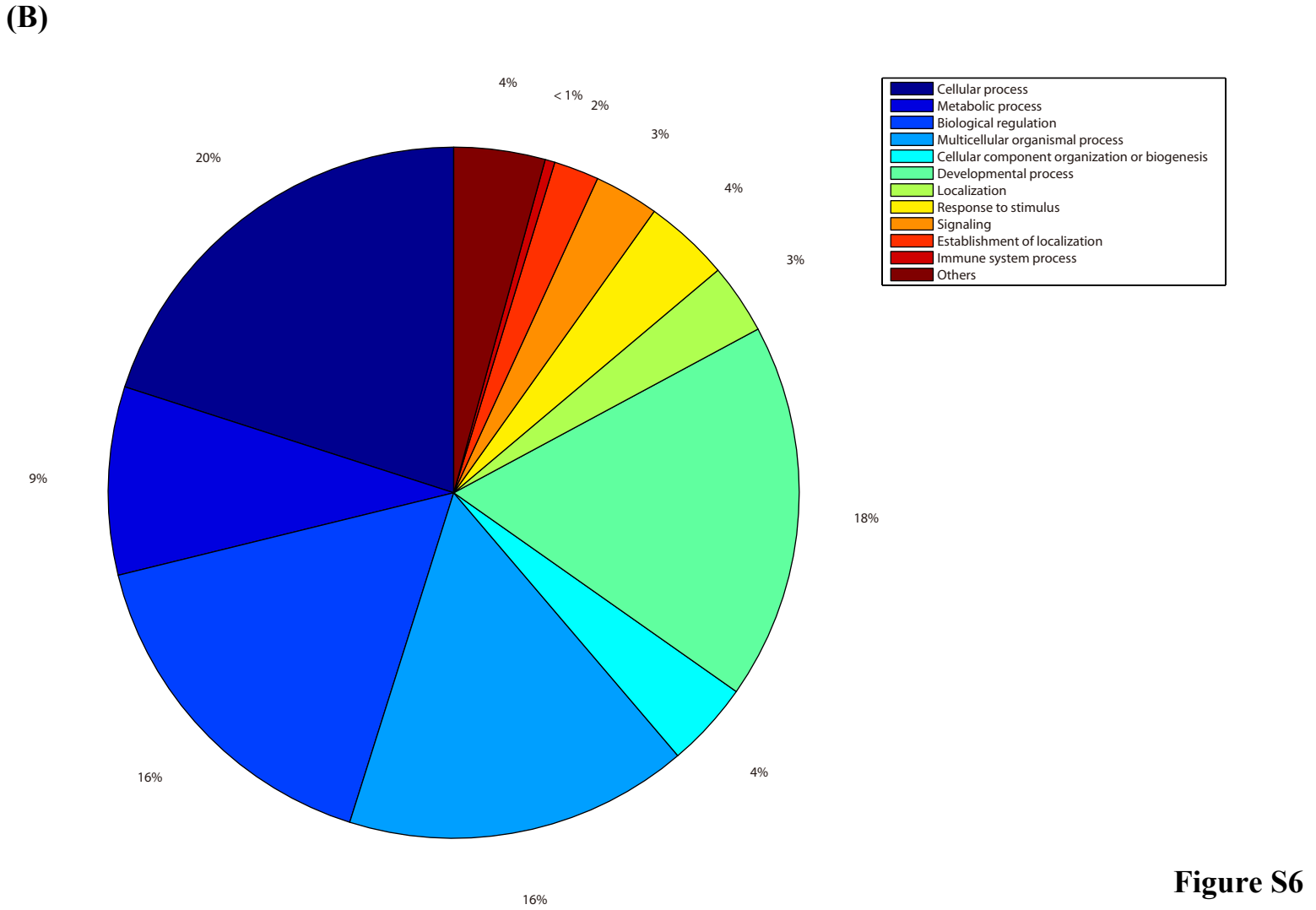

**Figure S6**

(C)

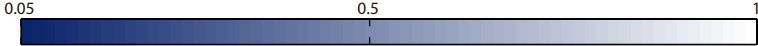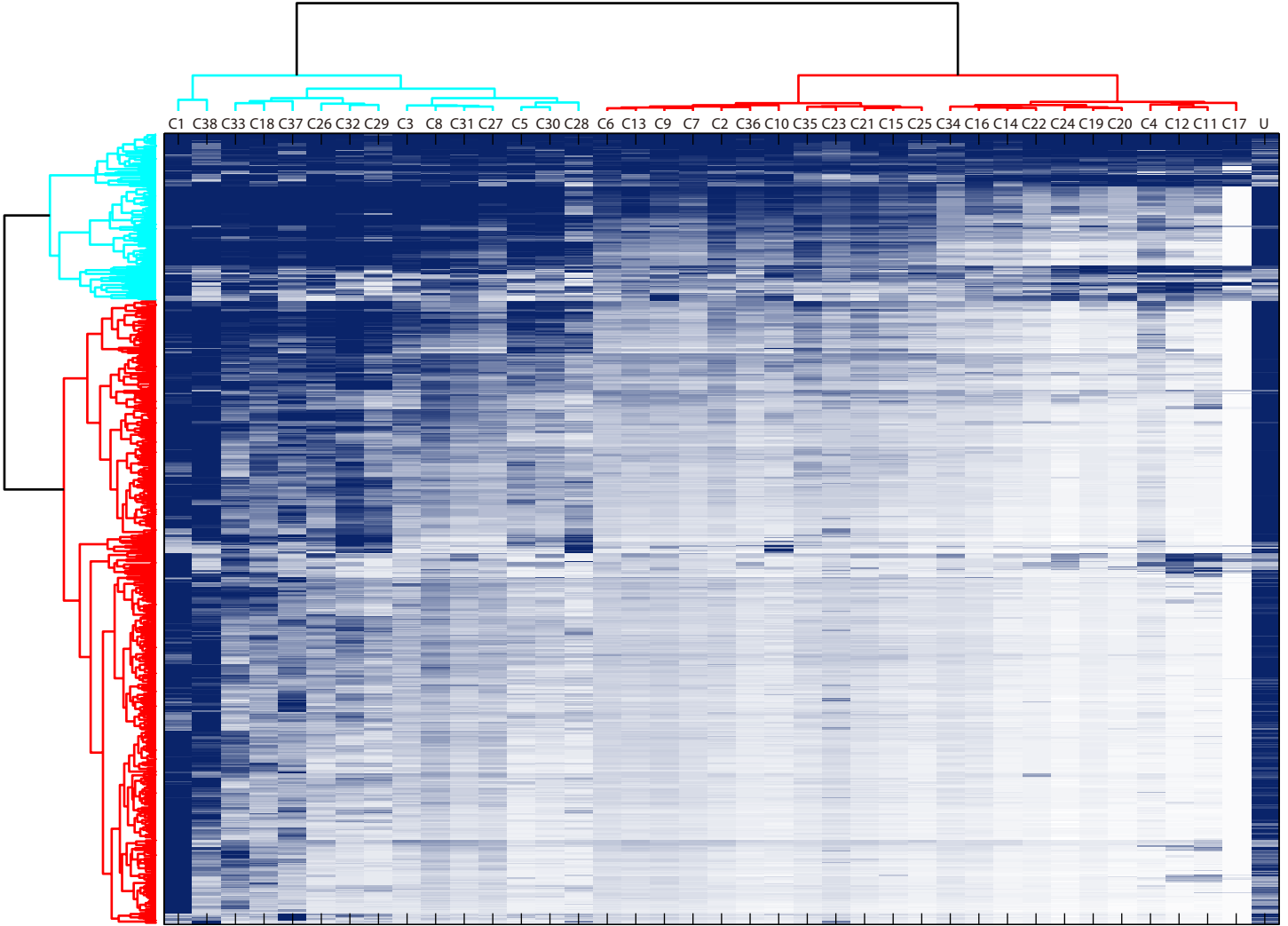

(D)

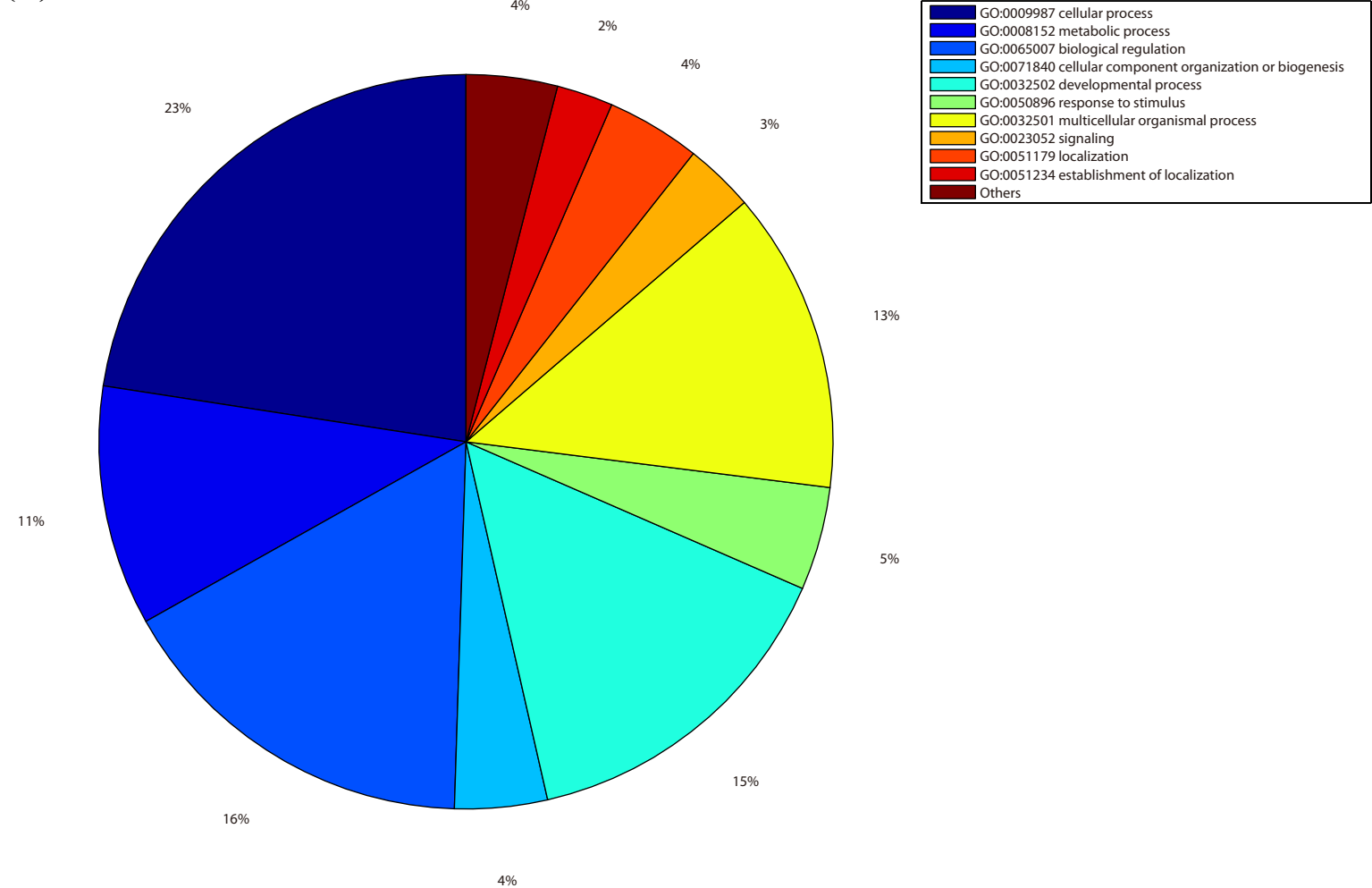

Supplement: Figure S6 — GOMO analysis of over-represented motifs within cell type-specific and ubiquitous CTCF-binding sites across 38 cell types. (A) GO analysis of the first three over-represented motifs within cell type-specific and ubiquitous CTCF-binding sites. Clustering of 38 cell types based on common GO nodes. This resulted in a list of 443 common GO nodes. Hierarchical clustering of both the cell types and the common GO nodes was performed using the calculated EASE scores. The relationship between the color intensity and EASE score is illustrated by the color bar. Gray indicates that an EASE score was not calculated for that GO node. The cell type is denoted by the letter and number combination at the top of every column. C1–C38 = CTCF-binding sites of 38 cell types, U = ubiquitous CTCF-binding site. (B) Summary of biological processes regulated by genes related to the first three overrepresented motifs within cell type-specific and ubiquitous CTCF-binding sites. Annotations were obtained from the Gene Ontology database. (C) GO analysis of the first five over-represented motifs within cell type-specific and ubiquitous CTCF-binding sites. Clustering of 38 cell types based on common GO nodes. This resulted in a list of 700 common GO nodes. Hierarchical clustering of both the cell types and the common GO nodes was performed using the calculated EASE scores. The relationship between the color intensity and EASE score is illustrated by the color bar. Gray indicates that an EASE score was not calculated for that GO node. The cell type is denoted by the letter and number combination at the top of every column. C1–C38 = CTCF-binding sites of 38 cell types, U = ubiquitous CTCF-binding site. (D) Summary of biological processes regulated by genes related to the first five over-represented motifs within cell type-specific and ubiquitous CTCF-binding sites. Annotations were obtained from the Gene Ontology database. (PDF) [file pone.0041374.s006.pdf]

Figure S7

(A)

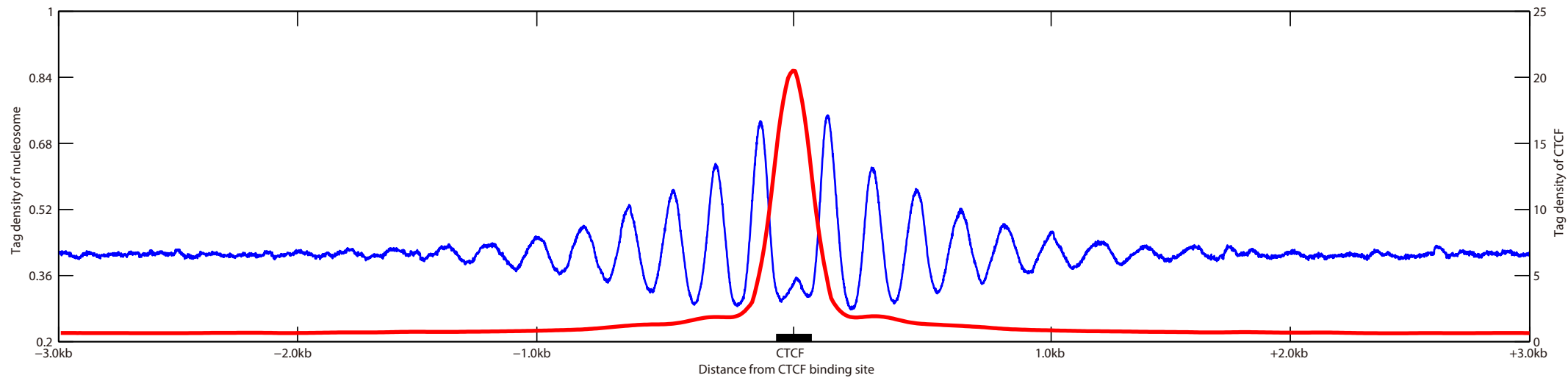

(B)

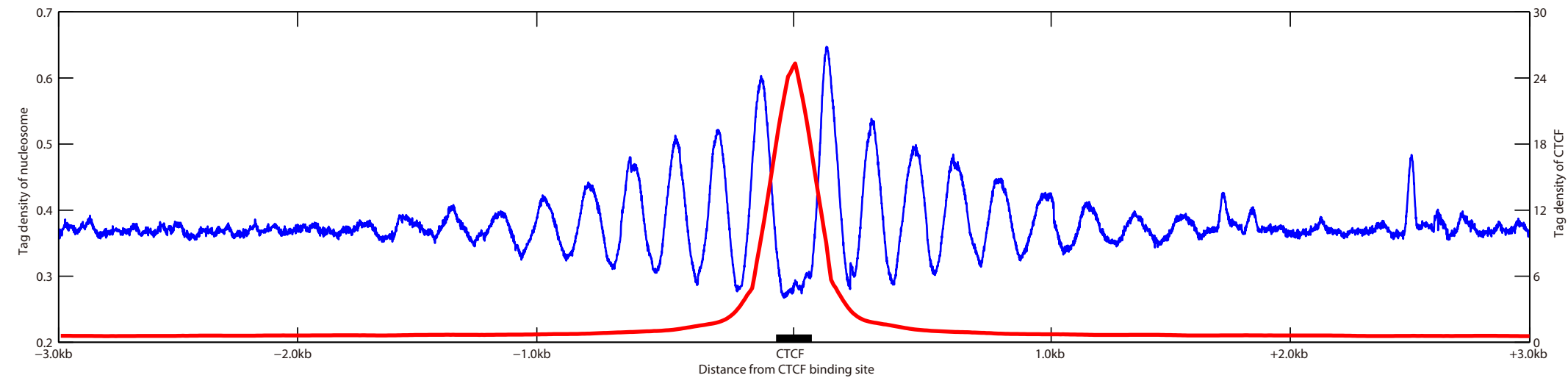

Supplement: Figure S7 — Chromatin structure near the CTCF-binding sites. Nucleosome (blue lines) and CTCF (red lines) profiles around CTCF-binding sites in K562 cells (A) and GM12878 cells (B) are illustrated. Distances from the CTCF-binding sites are plotted along the x-axis. Left and right y-axis represents the normalized tag densities of nucleosome and CTCF, respectively. (PDF) [file pone.0041374.s007.pdf]

Figure S8

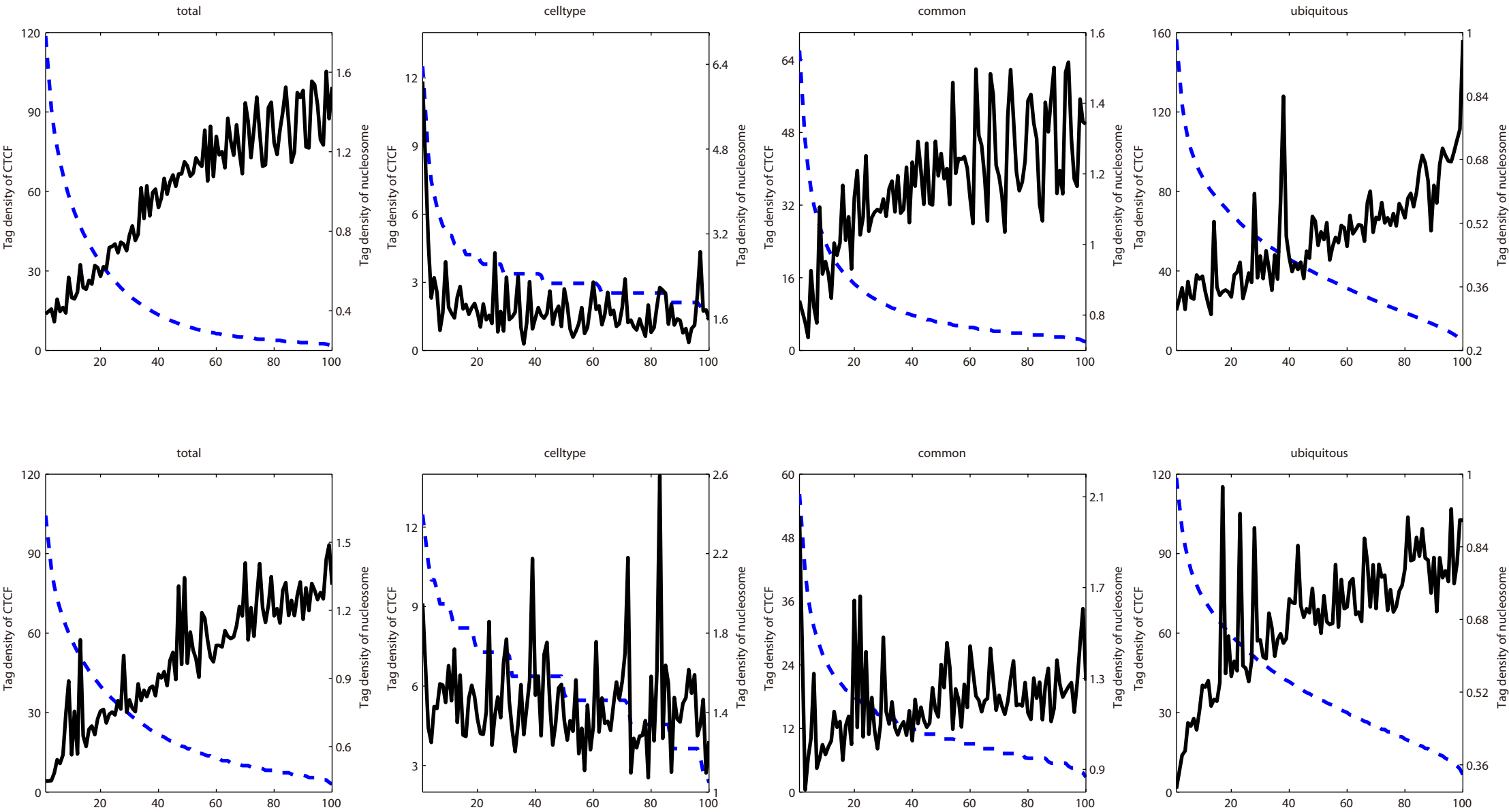

Supplement: Figure S8 — Scatter correlations between CTCF and nucleosome positioning in K562 and GM12878 cell types. Correlation between CTCF and nucleosome positioning in K562 (A–D) and GM12878 (E–H) cell types. Total (A, E), cell type-specific (B, F), common (C, G), and ubiquitous (D, H) CTCF-binding sites were grouped into 100 sets (dot) based on their levels (from high to low, left to right on the x-axis). The average tag densities of CTCF and of histone modifications were calculated for each group and plotted according to the average tag density of CTCF (right y-axis) and the histone methylation (left y-axis). (PDF) [file pone.0041374.s008.pdf]

(A)

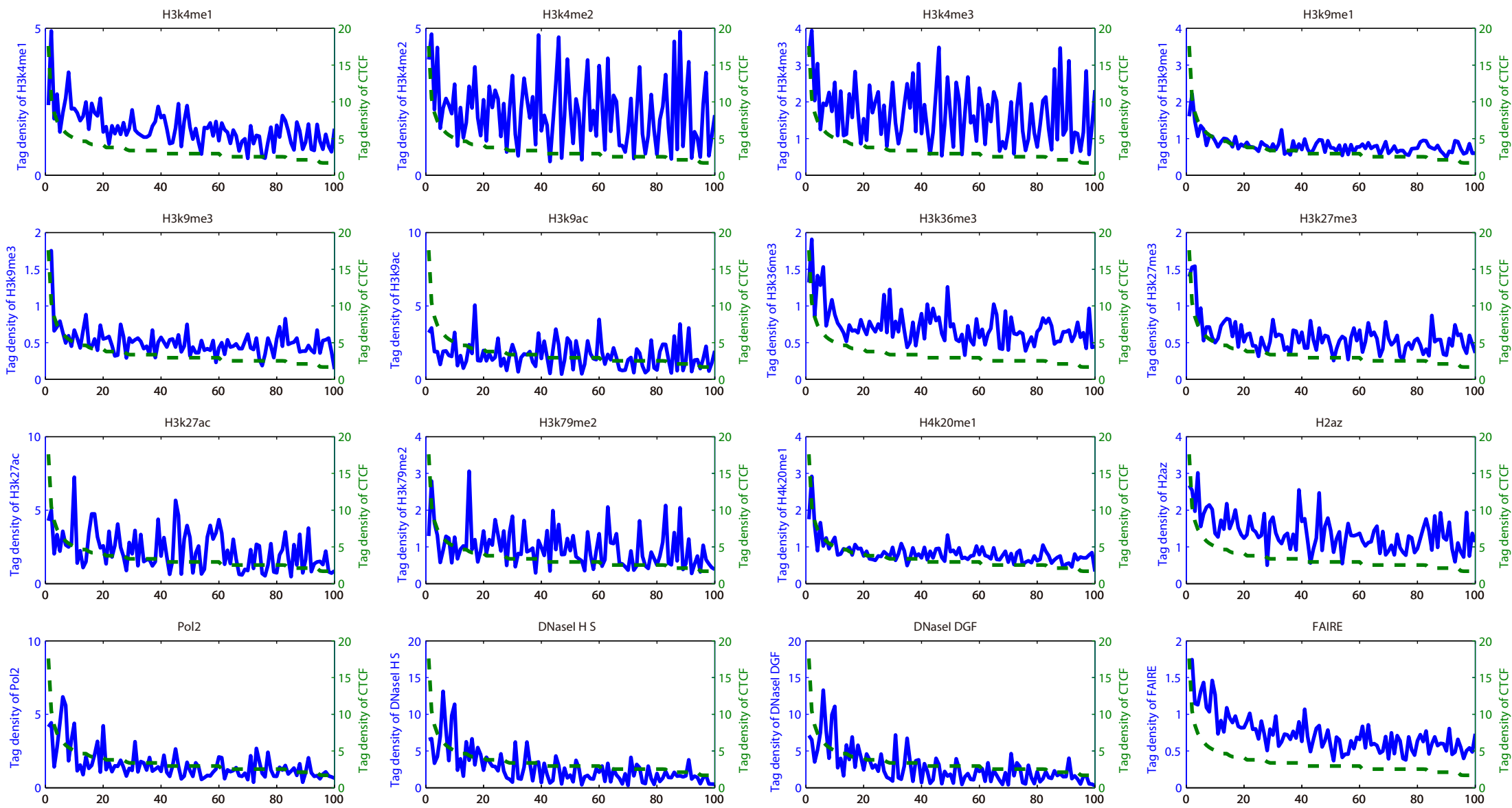

Figure S9

**(B)**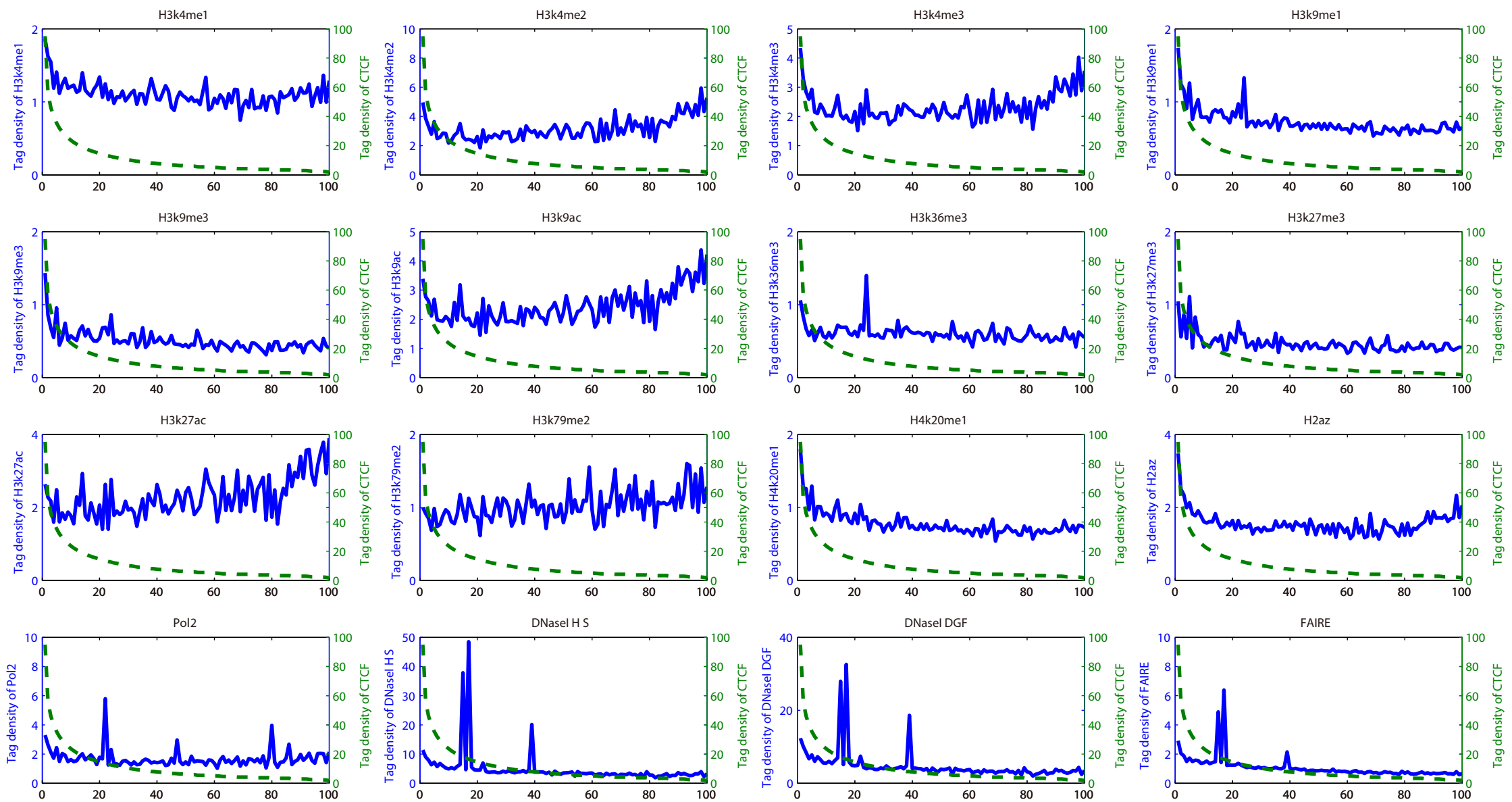

(C)

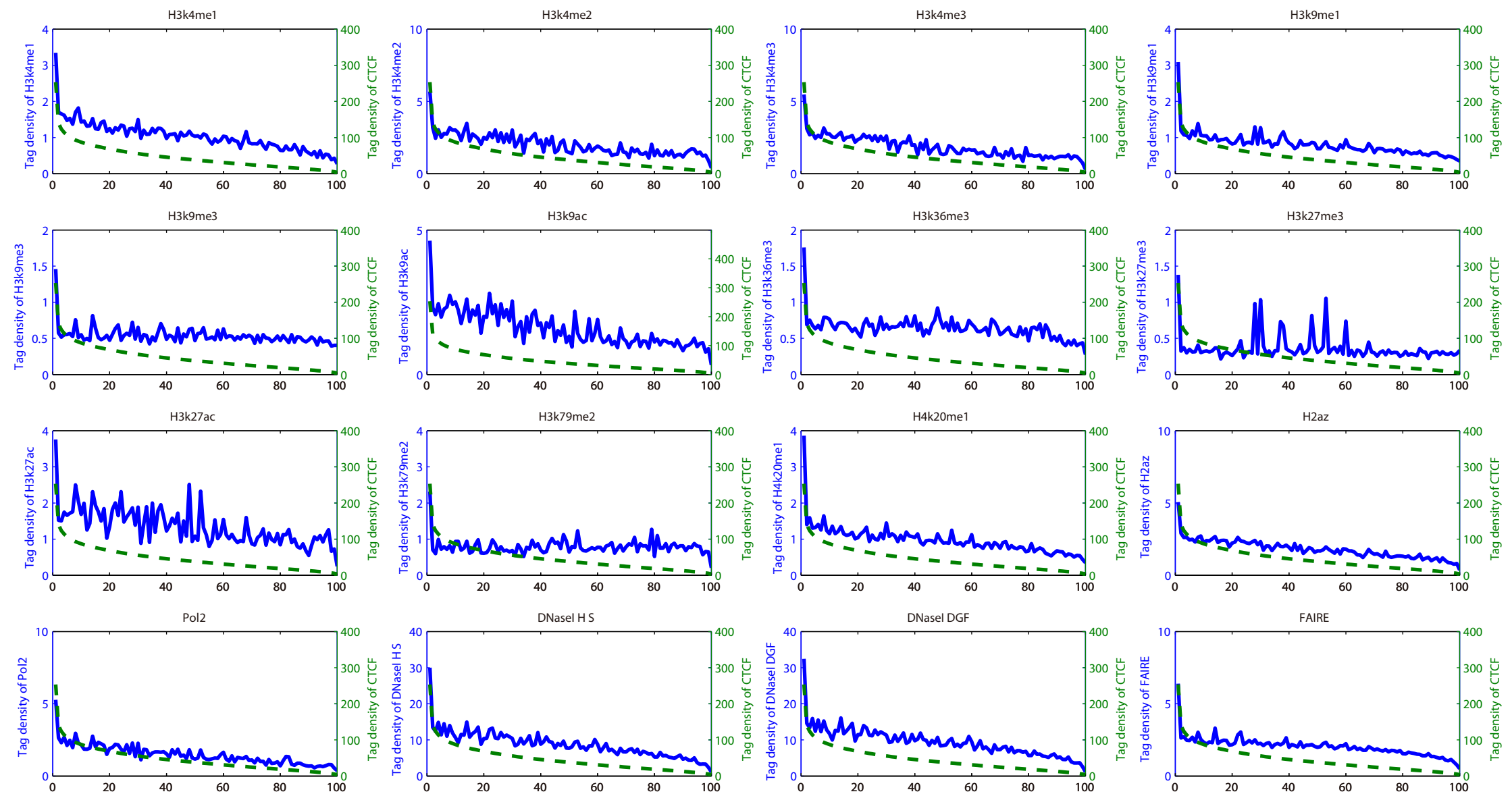

Supplement: Figure S9 — Scatter correlations between CTCF and open chromatins and histone modifications in K562 cells. Cell type-specific (A), common (B), and ubiquitous (C) CTCF-binding sites were grouped into 100 sets (dot) based on their levels (from high to low, left to right on the x-axis). The average tag densities of CTCF and of open chromatins were calculated for each group and plotted according to the average tag densities of CTCF (right y-axis) and the open chromatin (left y-axis). (PDF) [file pone.0041374.s009.pdf]

(A)

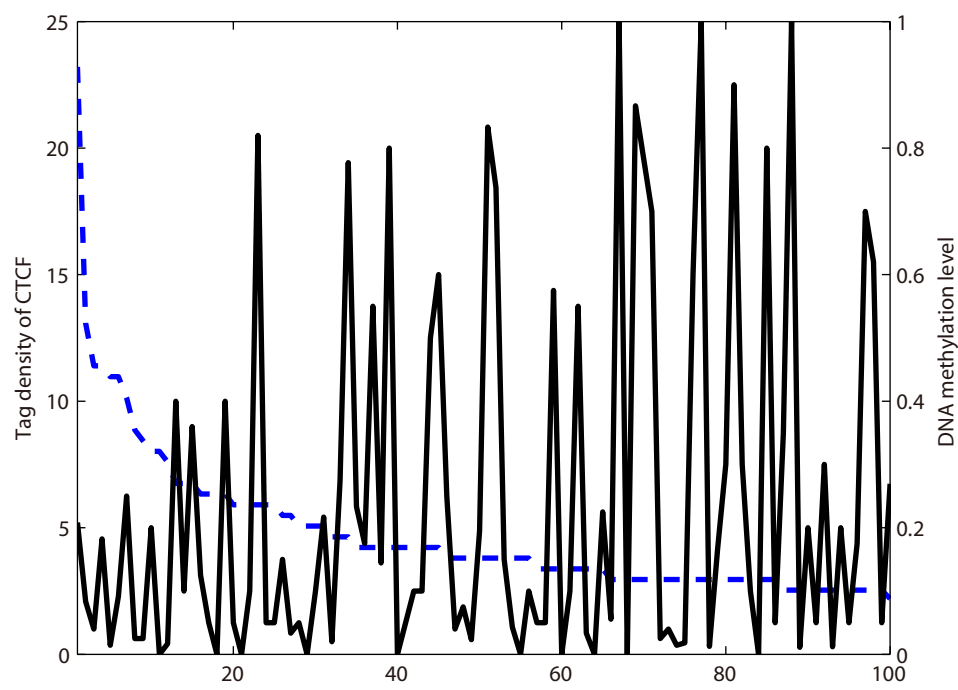

(B)

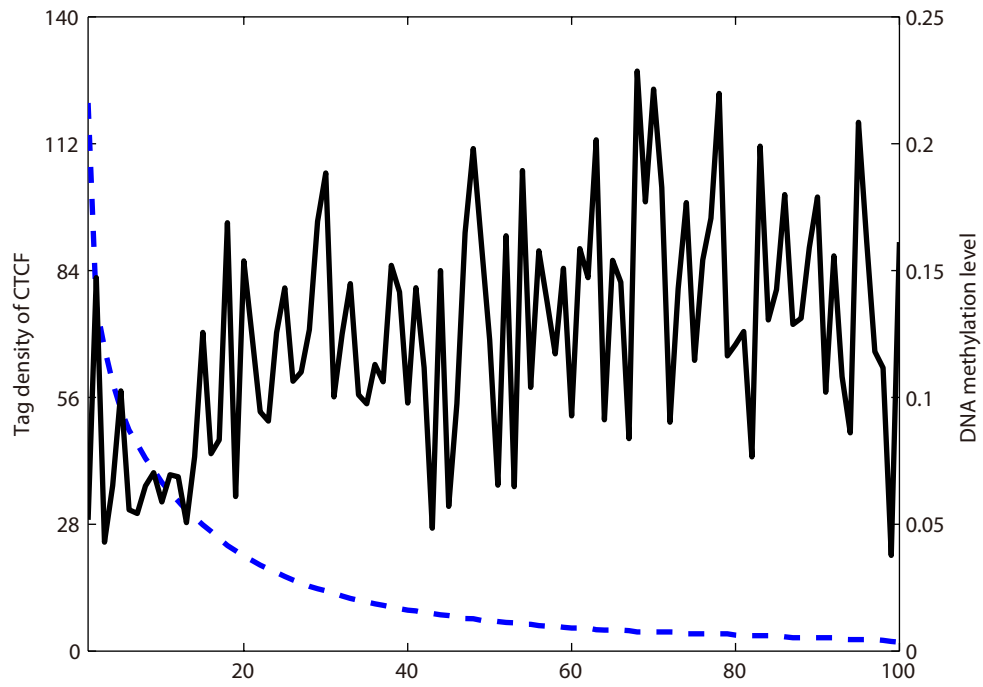

(C)

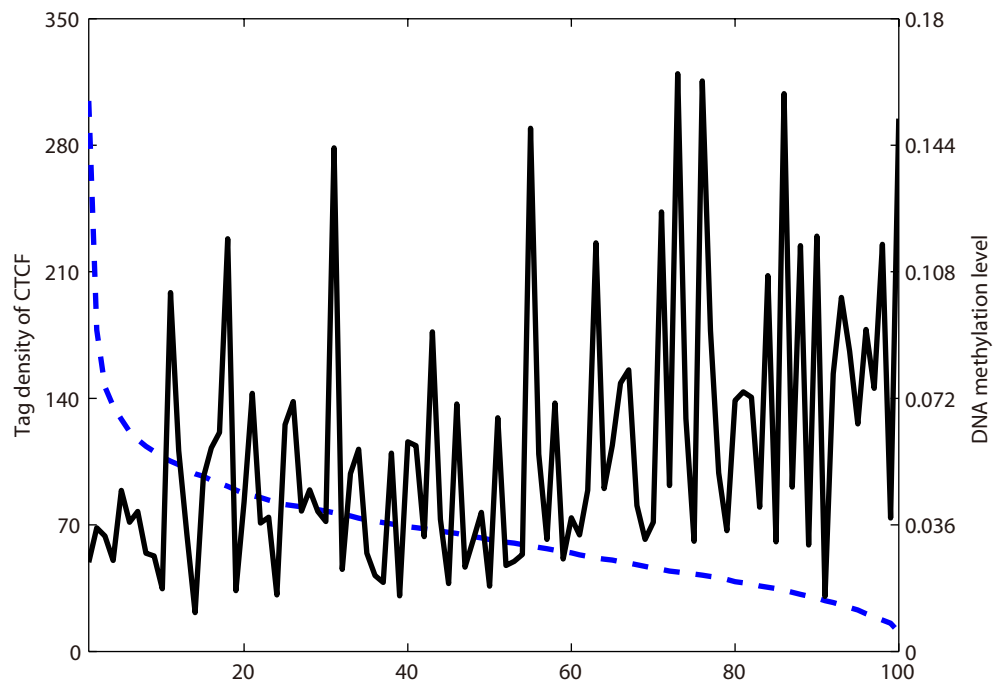

Figure S10

Supplement: Figure S10 — Correlation between CTCF and DNA methylation in K562 cells. Cell type specific (A), common (B), and ubiquitous (C) CTCF-binding sites were grouped into 100 sets (dot) based on their levels (from high to low, left to right on the x-axis). The average tag densities of CTCF and of DNA methylation levels were calculated for each group, and plotted according to the average tag density of CTCF (right y-axis) and the DNA methylation level (left y-axis). (PDF) [file pone.0041374.s010.pdf]

Figure S11

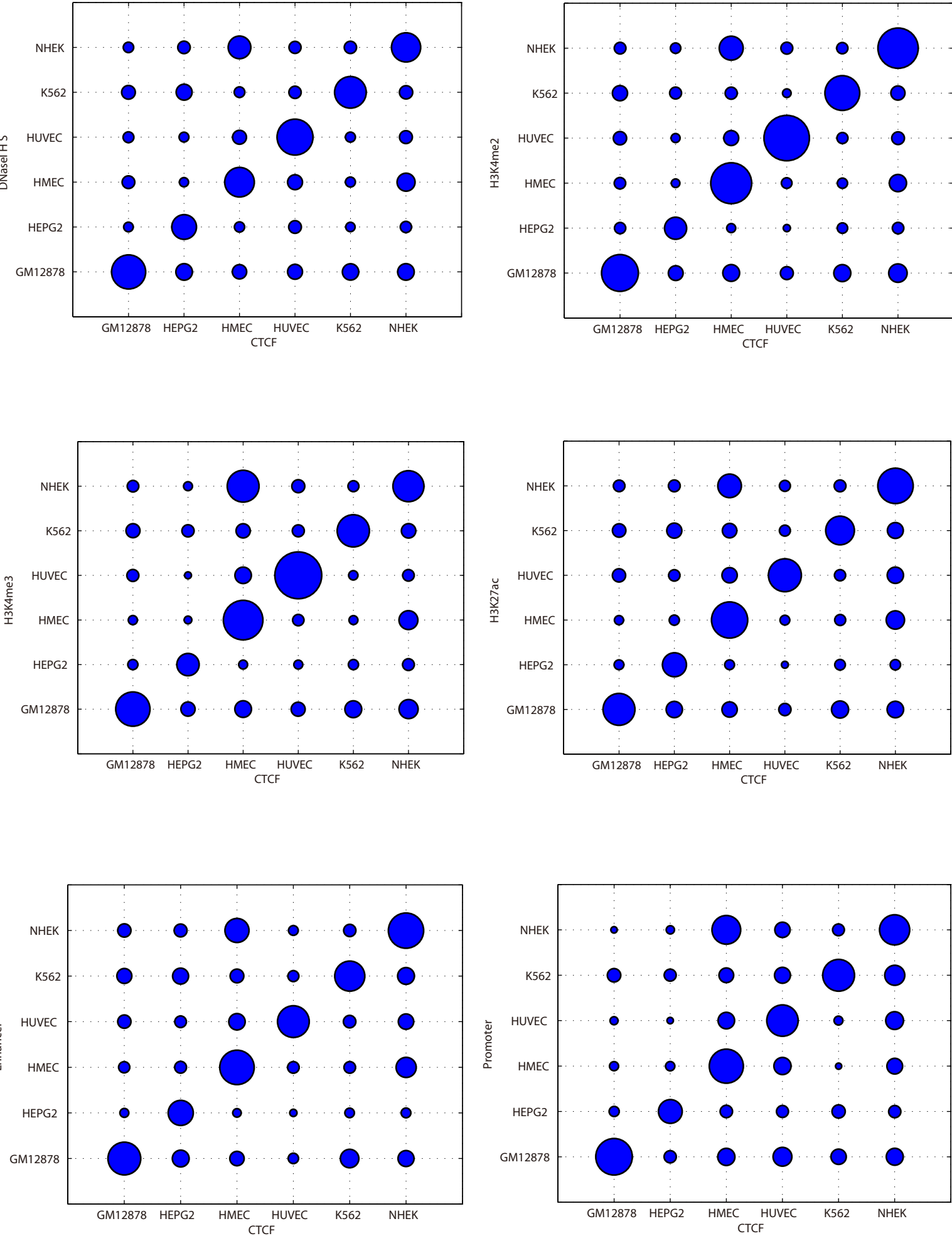

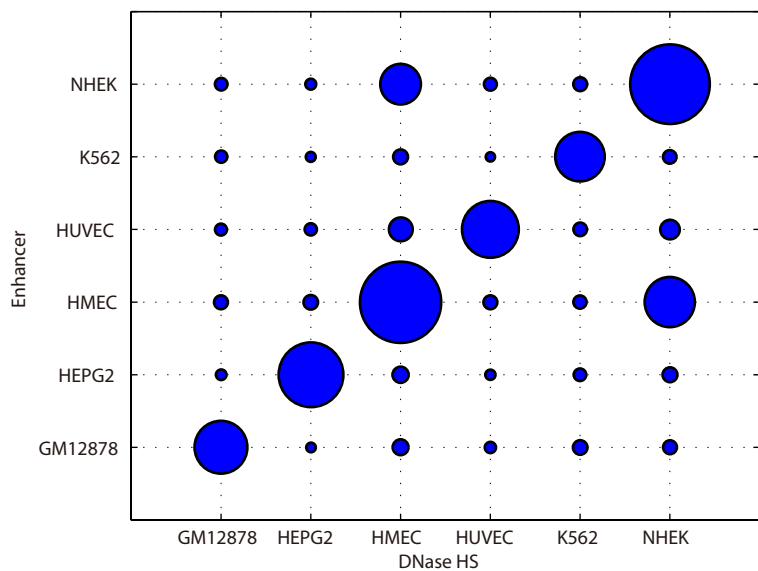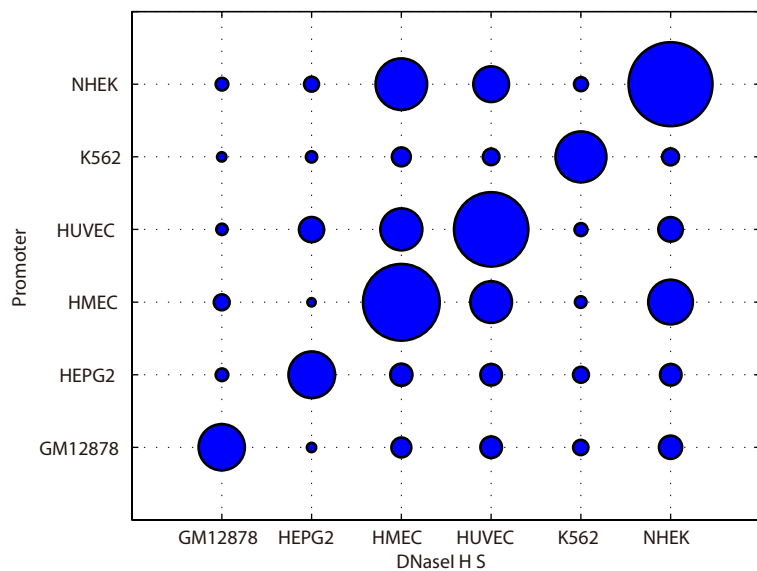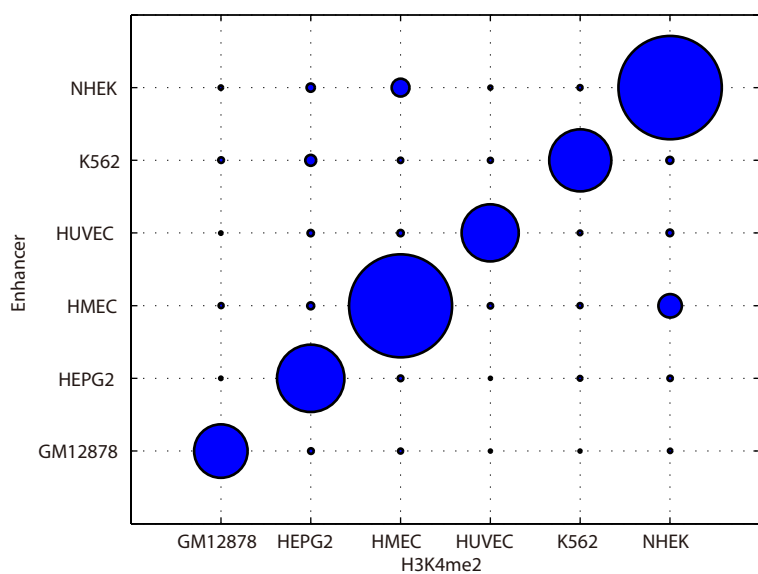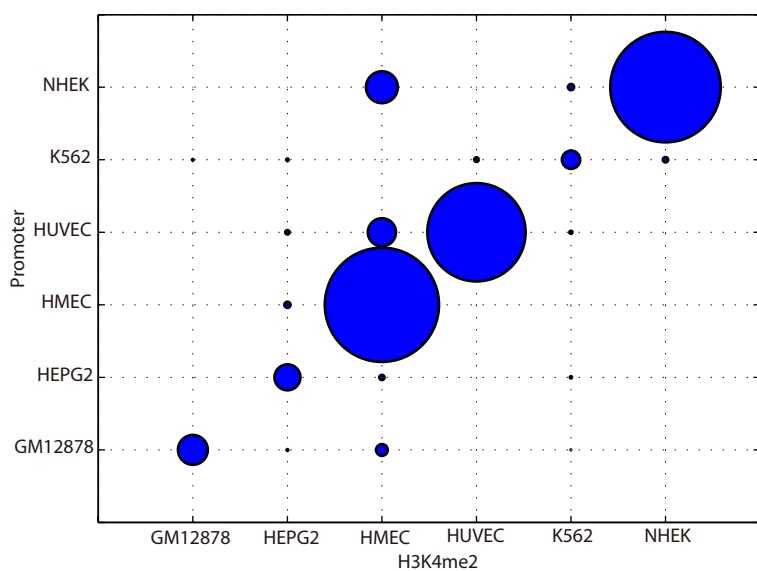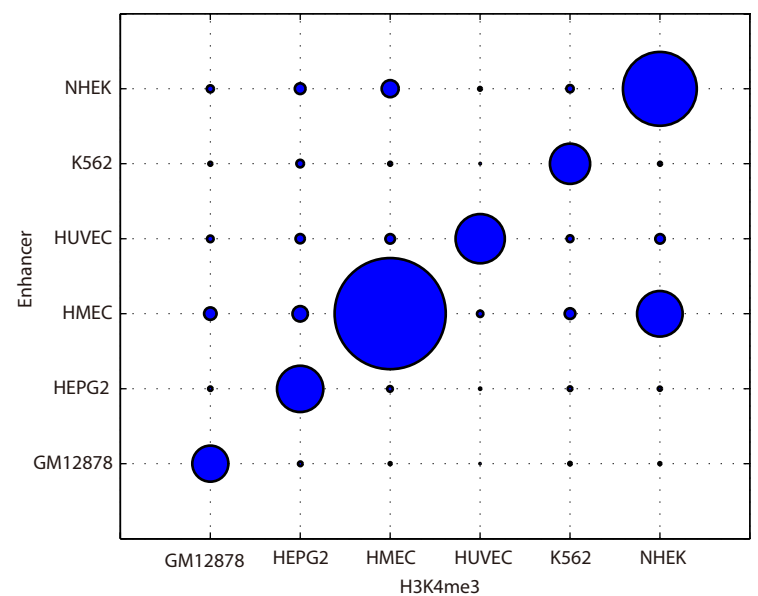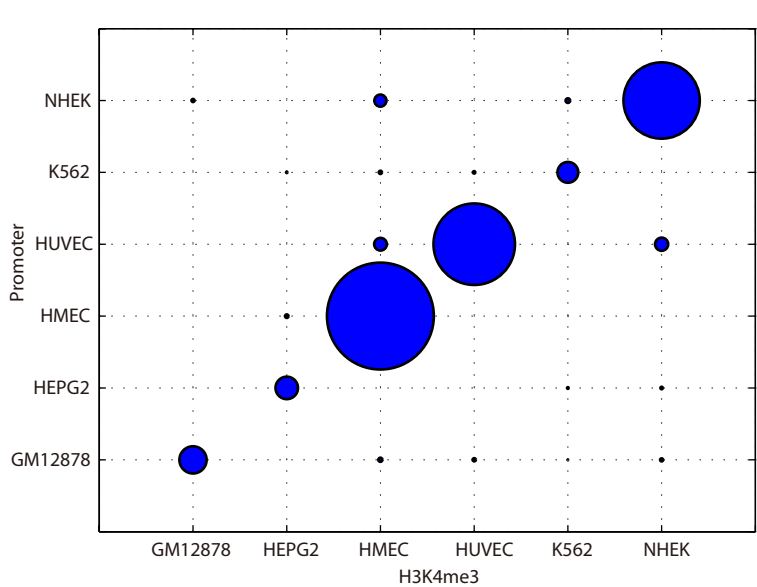

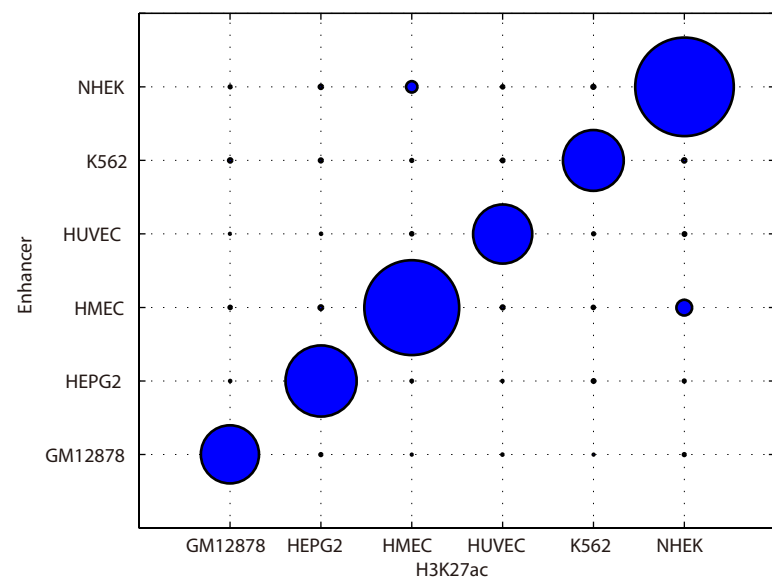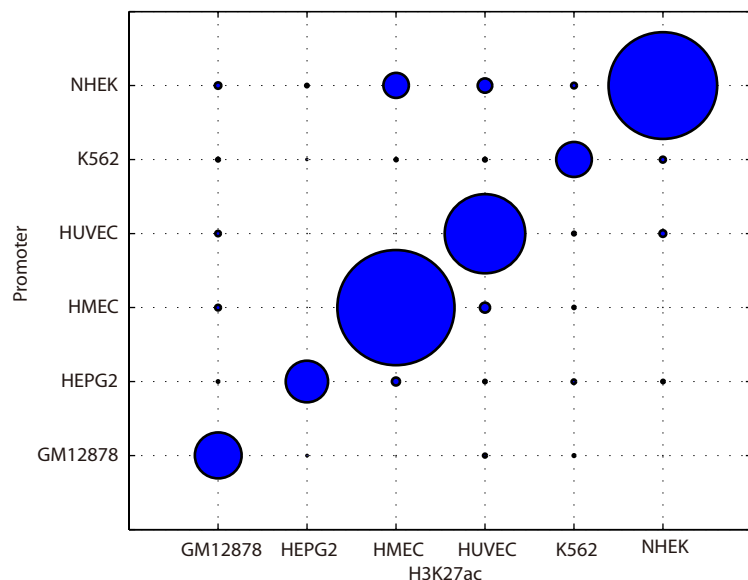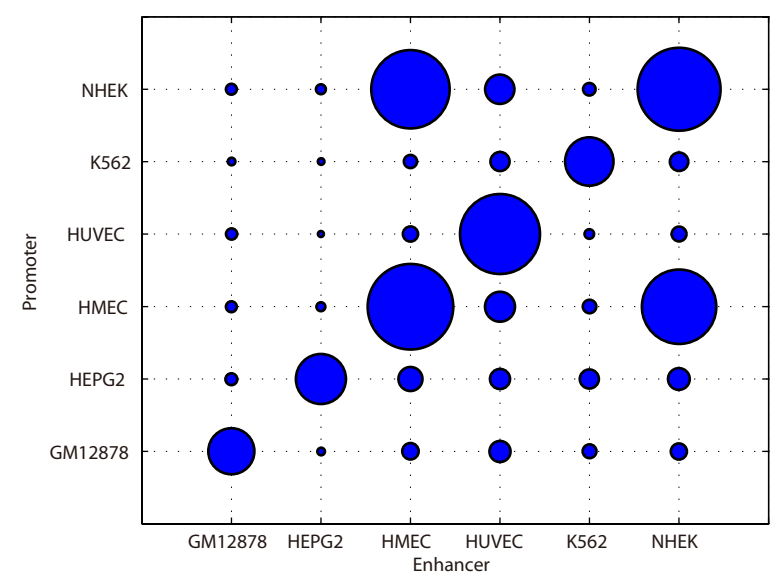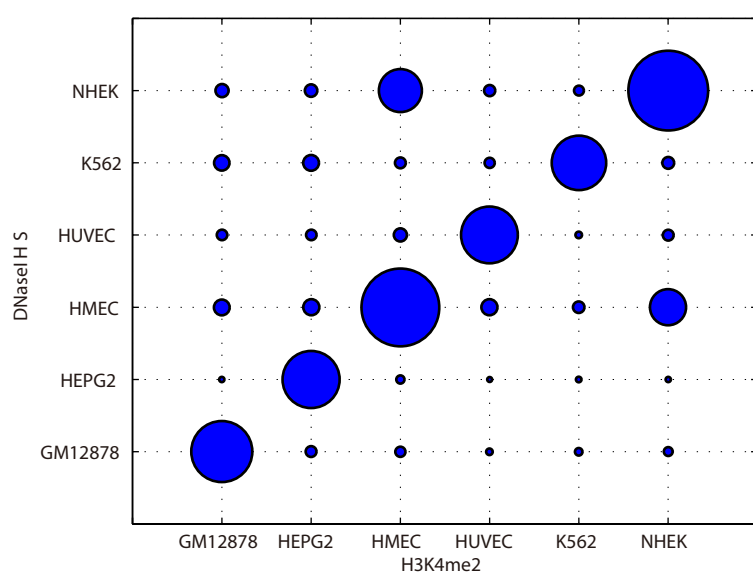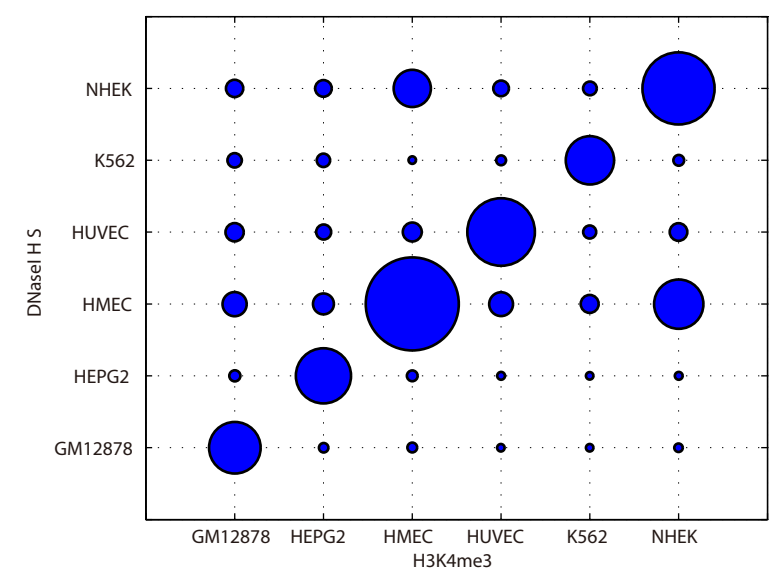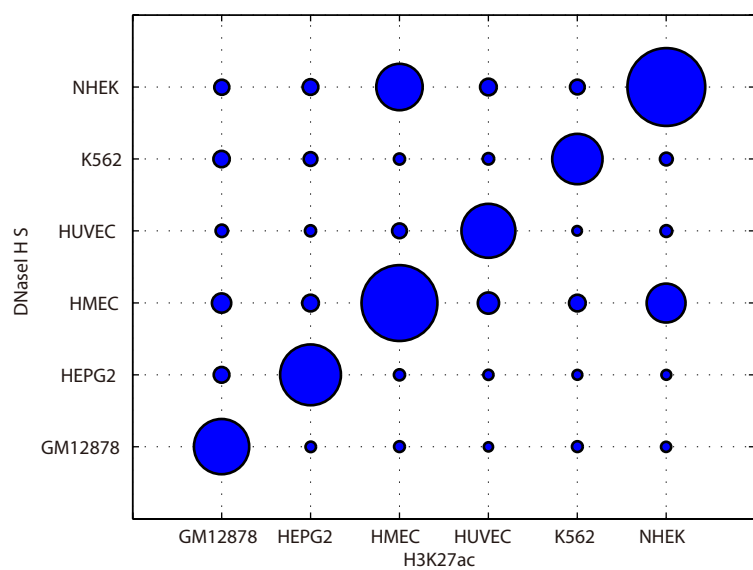

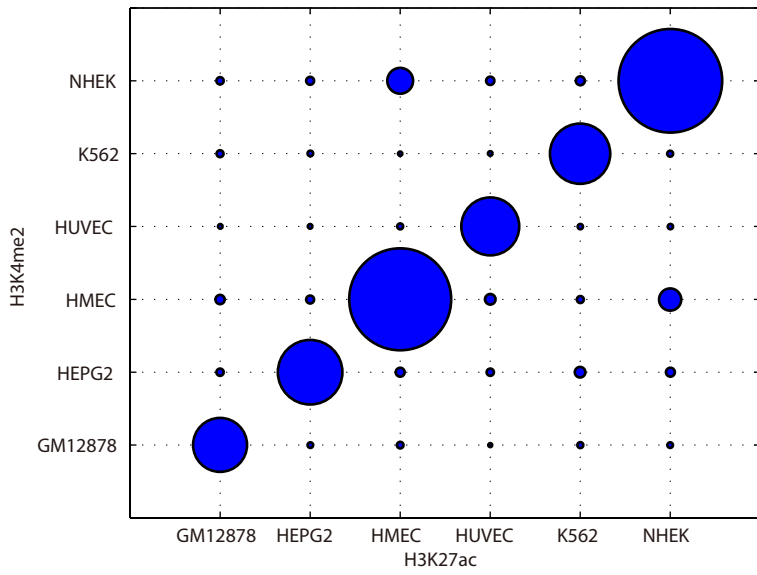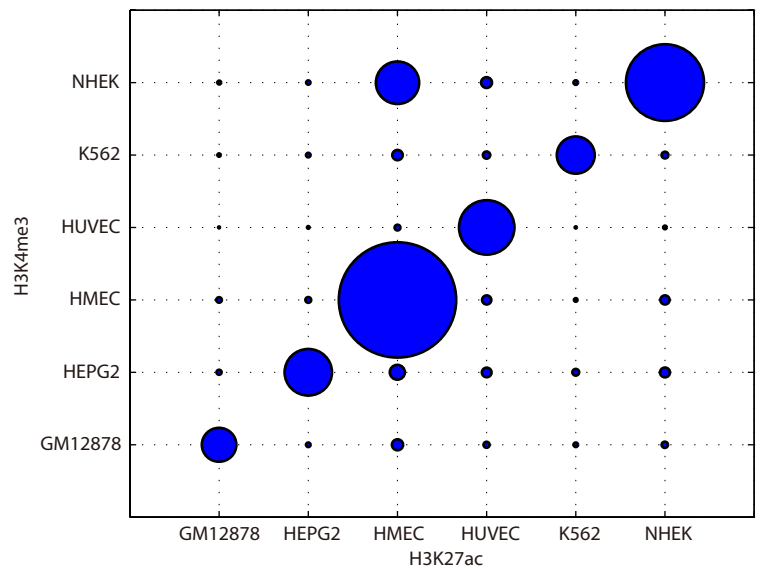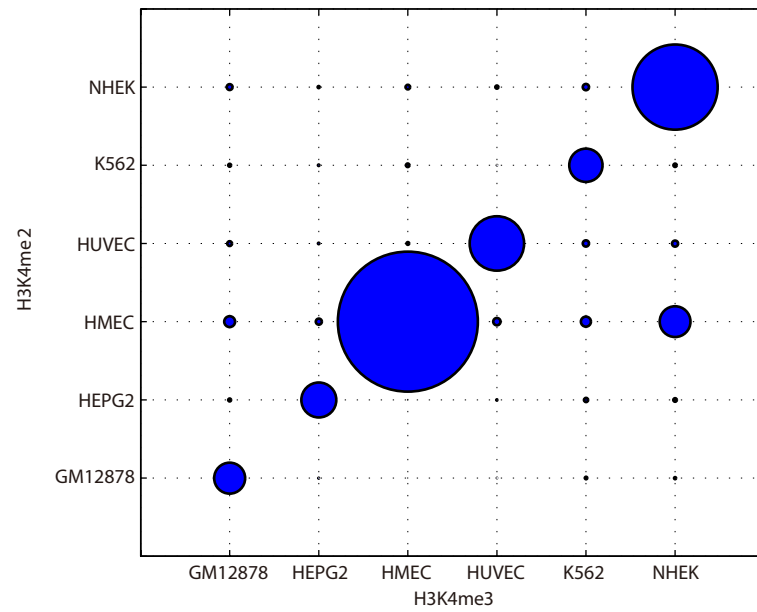

Supplement: Figure S11 — CTCF-binding sites colocalize with DNaseI HS sites, histone modified regions, enhancers, and promoters in a cell type-specific manner. CTCF-binding sites colocalize with histone modifications, DNaseI HS sites, strong enhancers and gene expression in a cell type-specific manner. Bubble size represents the level of enrichment. When no bubble is present, the value is zero (complete depletion). (PDF) [file pone.0041374.s011.pdf]

Figure S12

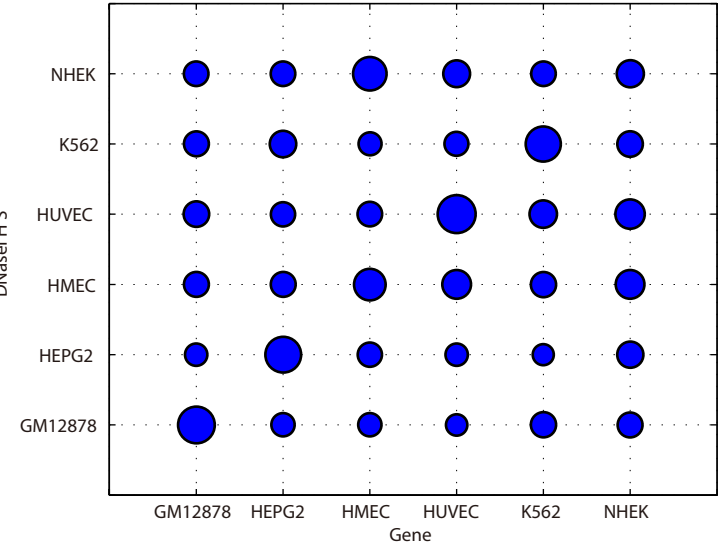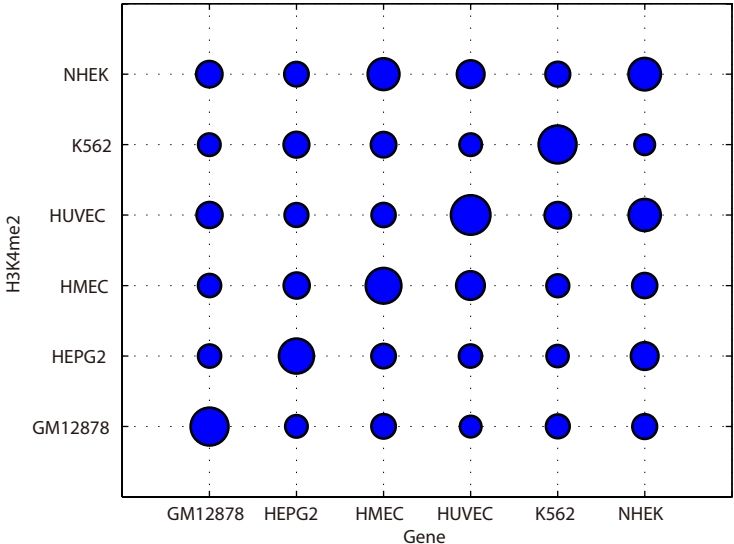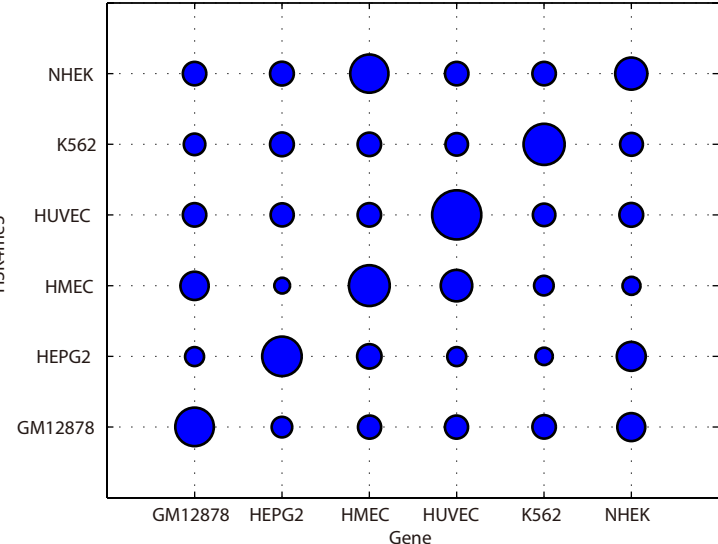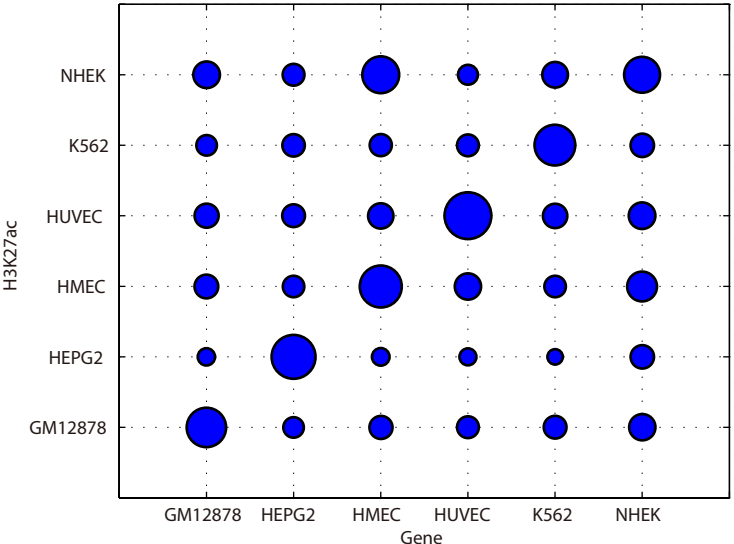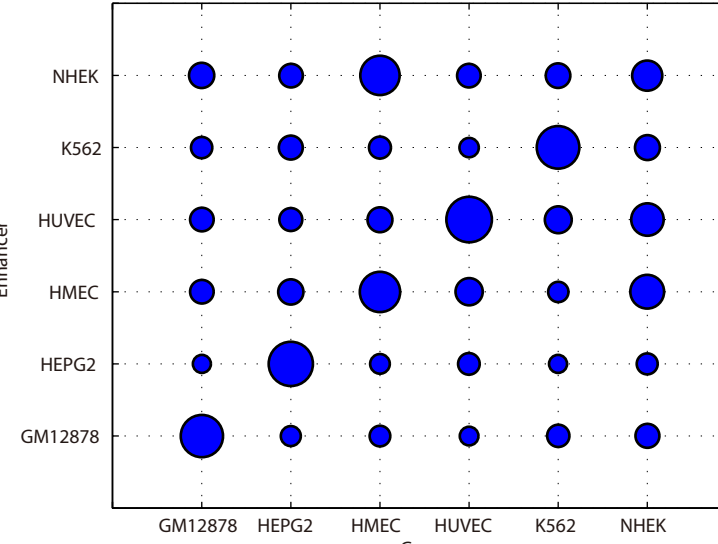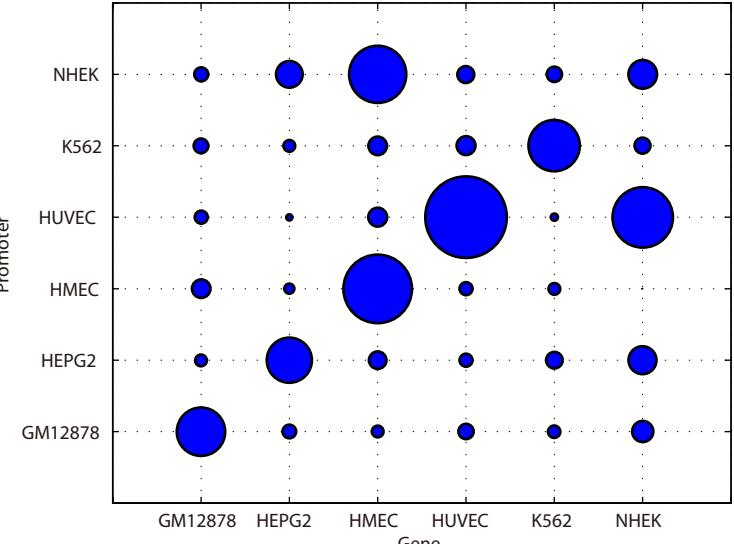

Supplement: Figure S12 — CTCF-binding sites, DNaseI HS sites, histone modification hits, enhancers, and promoters colocalize with gene expression in a cell type-specific manner. CTCF-binding sites (A), histone modifications (B–H), DNaseI HS sites (I), enhancers (J), and promoters (K) colocalize with gene expression in a cell type-specific manner. Bubble size represents the level of enrichment. When no bubble is present, the value is zero (complete depletion). (PDF) [file pone.0041374.s012.pdf]

**Chr1**

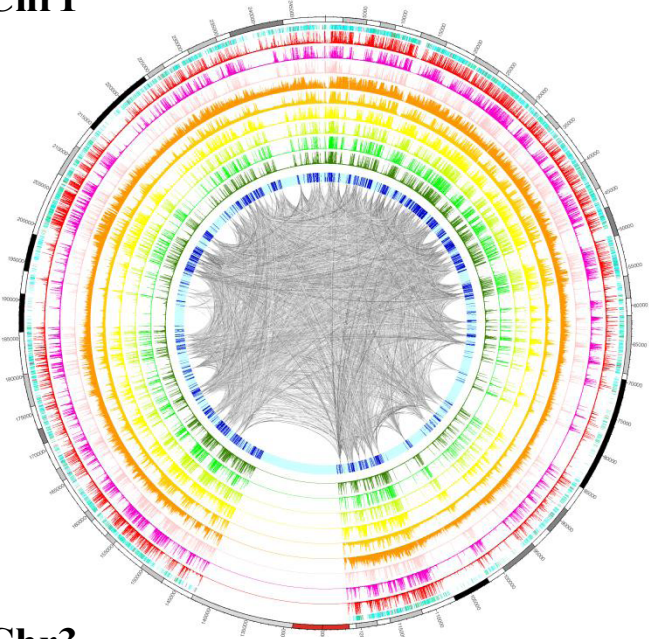

**Chr2**

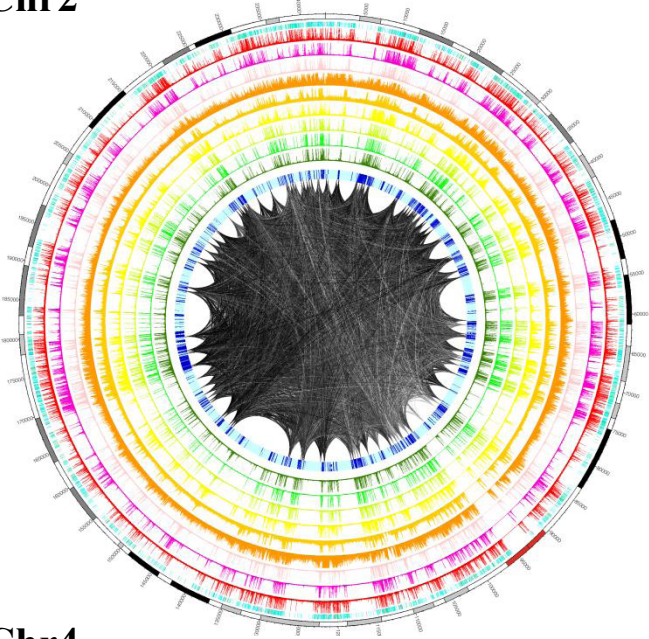

**Chr3**

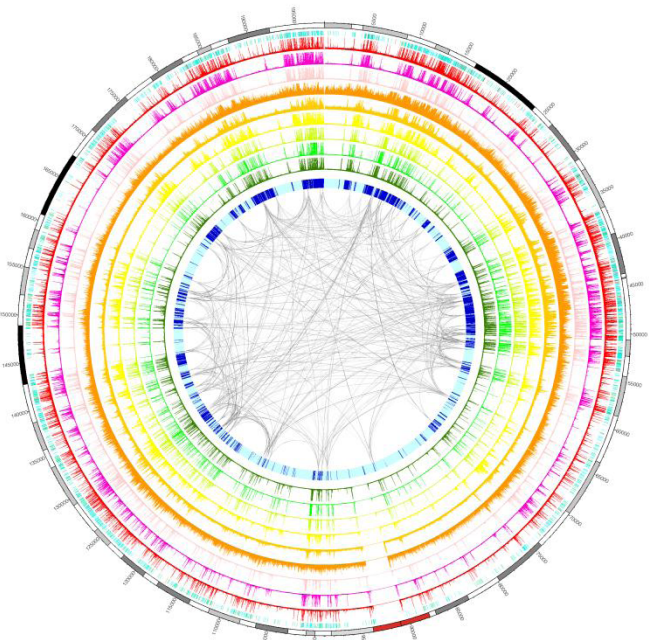

**Chr4**

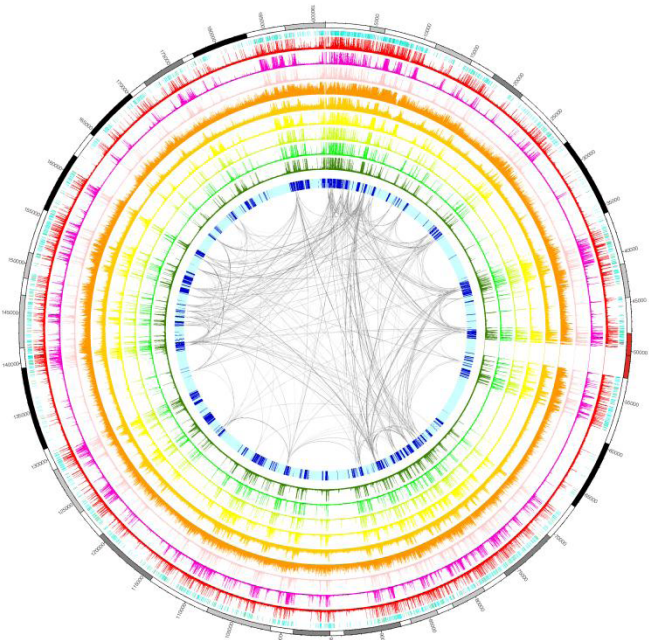

**Chr5**

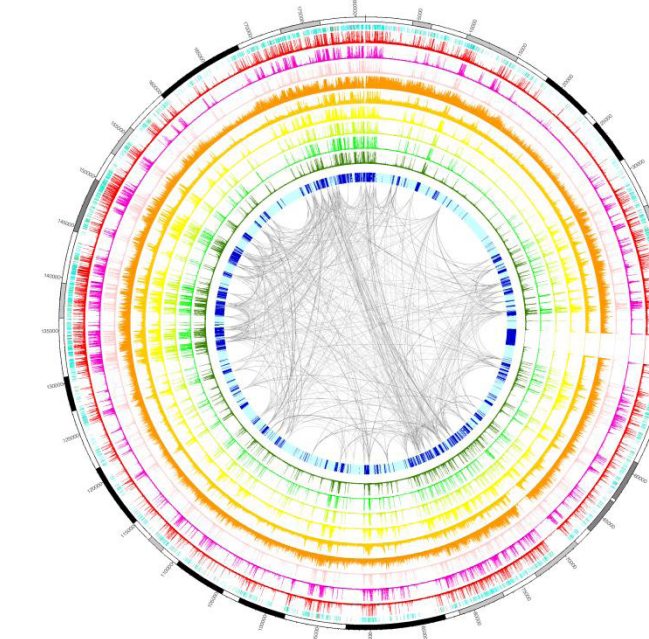

**Chr6**

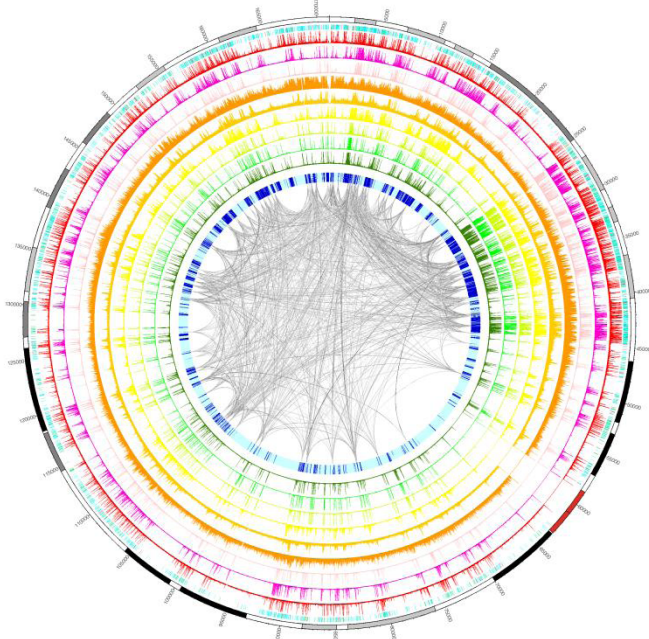

**Figure S13**

**Chr7**

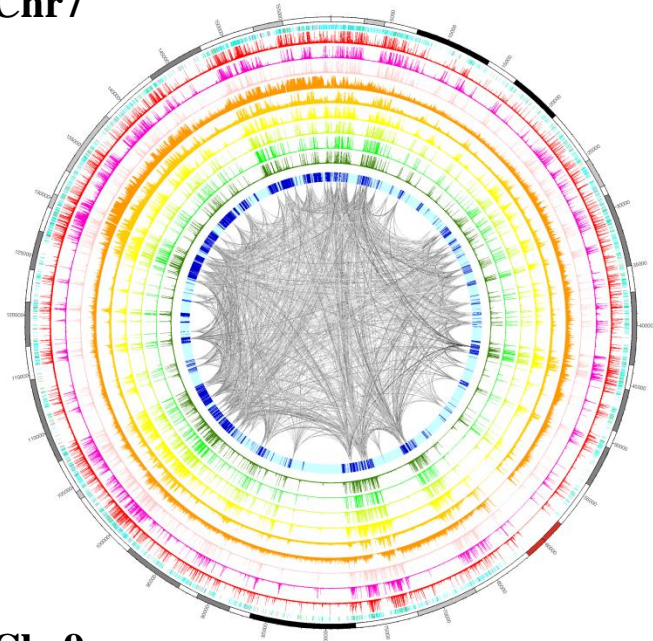

**Chr8**

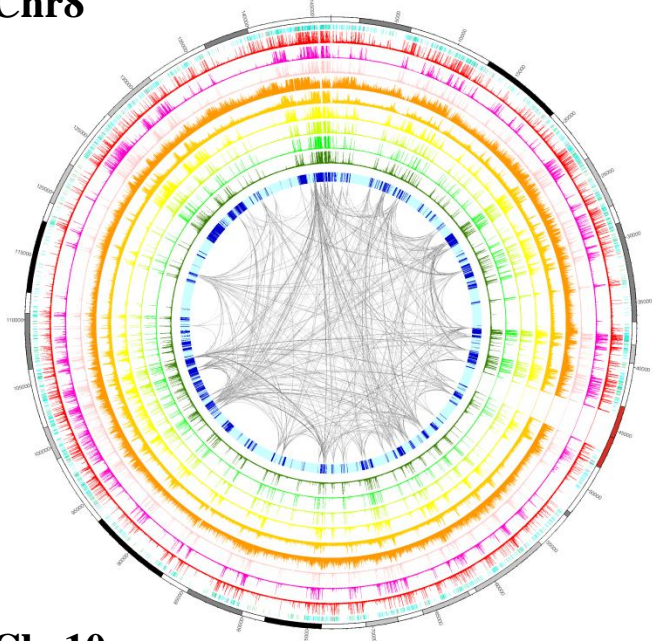

**Chr9**

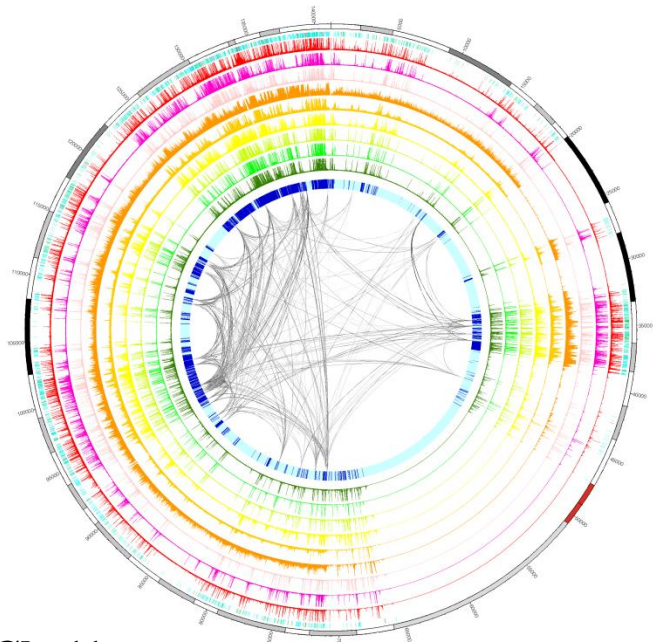

**Chr10**

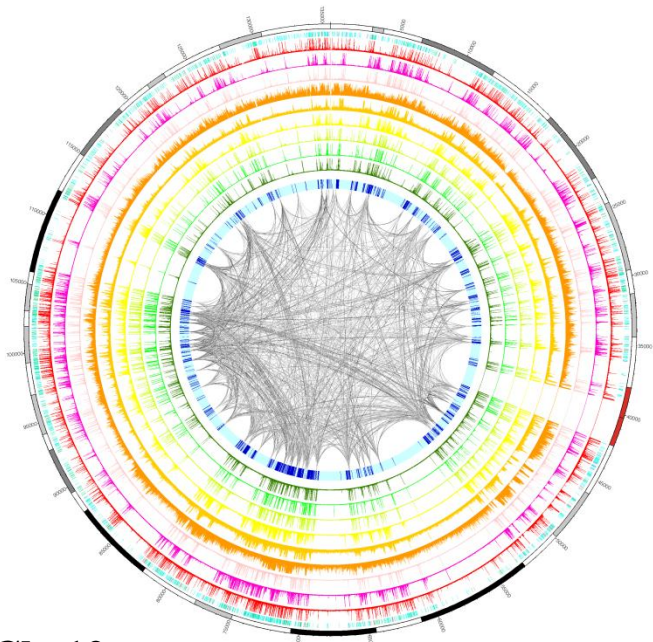

**Chr11**

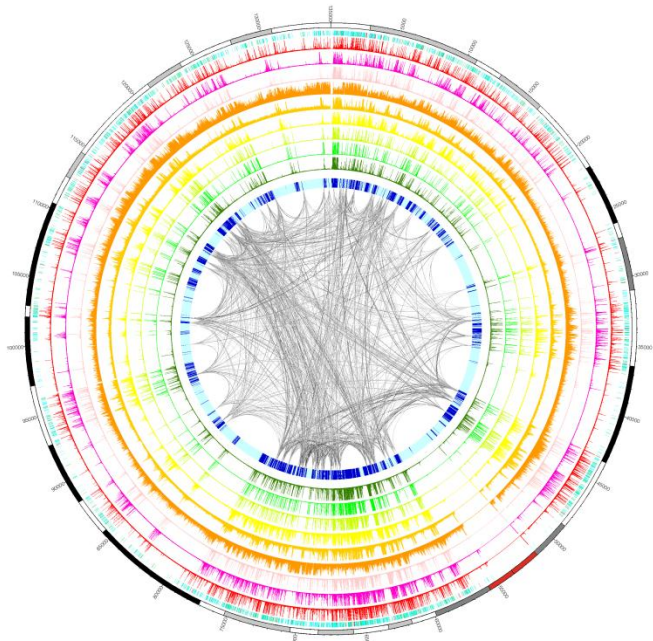

**Chr12**

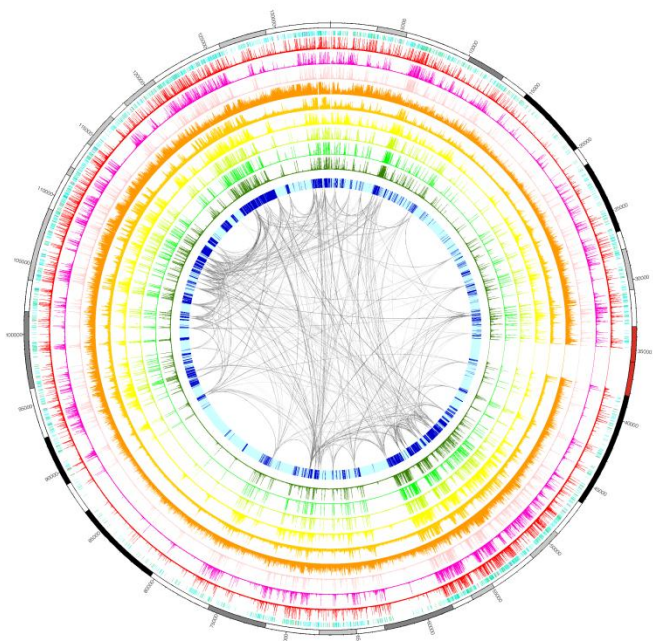

**Chr13**

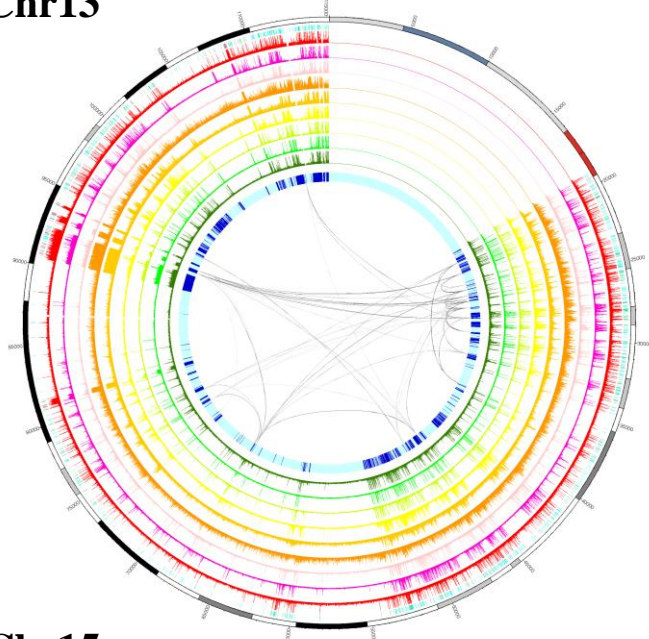

**Chr14**

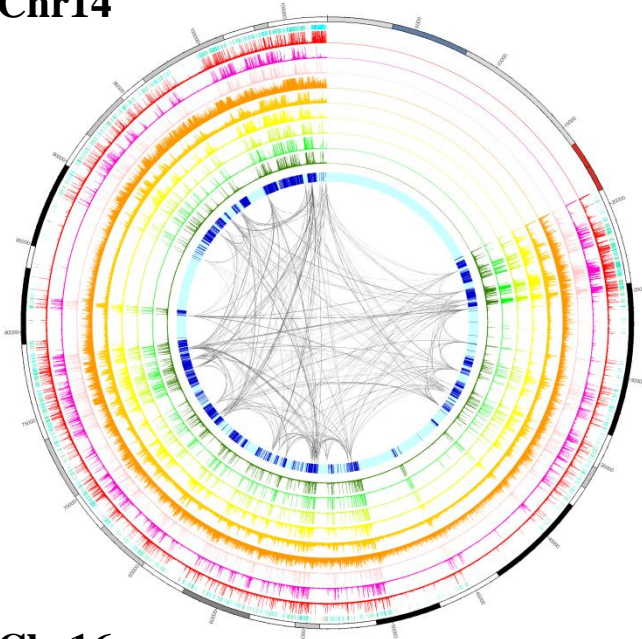

**Chr15**

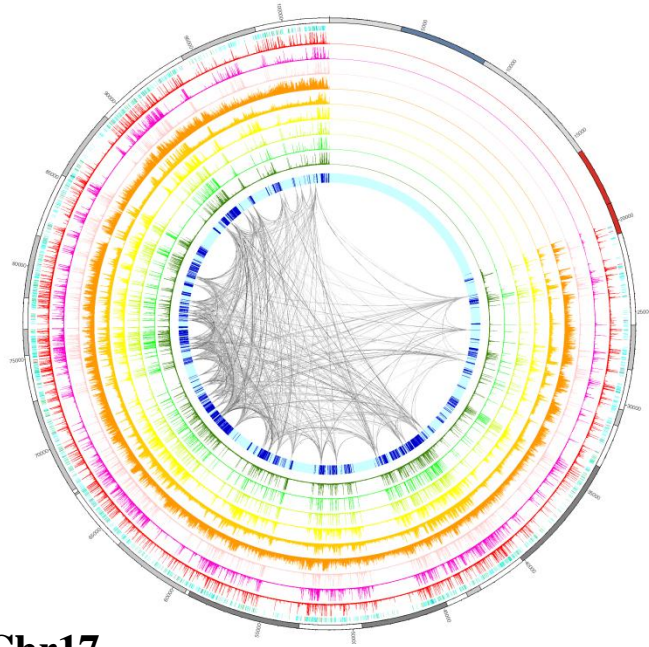

**Chr16**

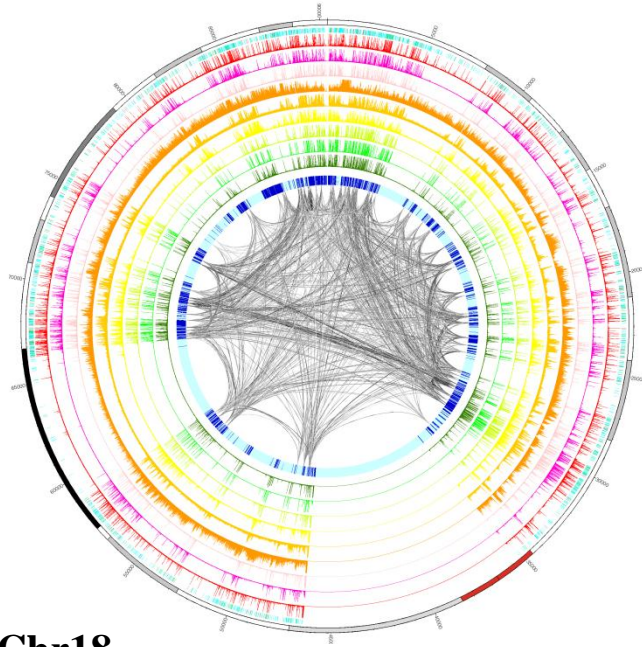

**Chr17**

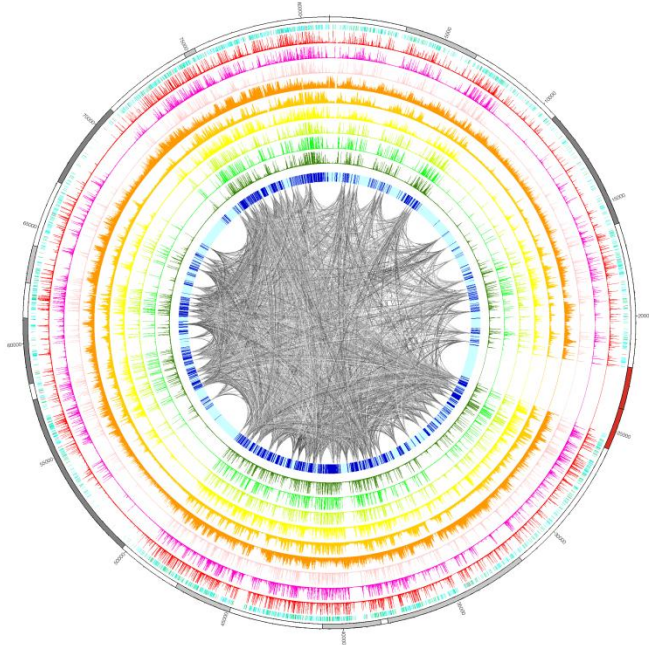

**Chr18**

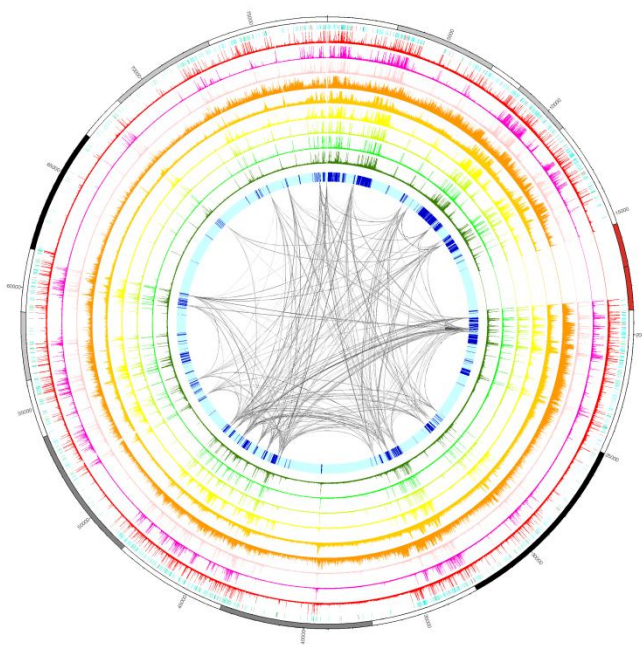

**Chr19**

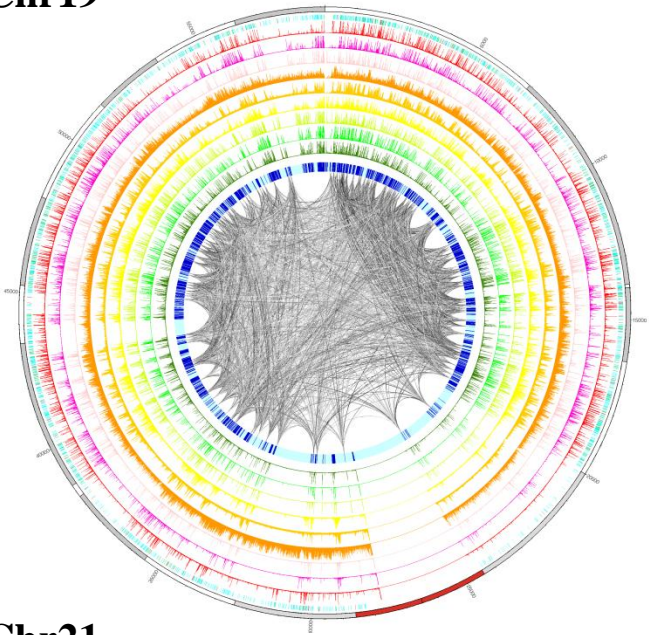

**Chr20**

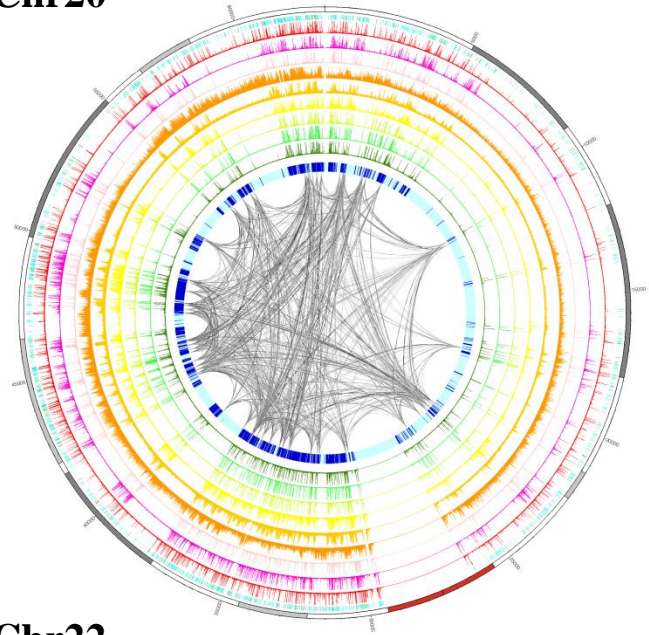

**Chr21**

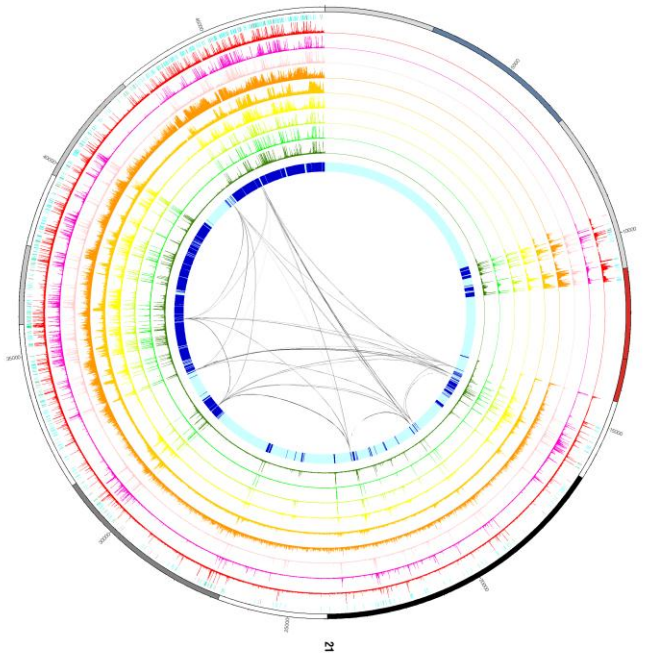

**Chr22**

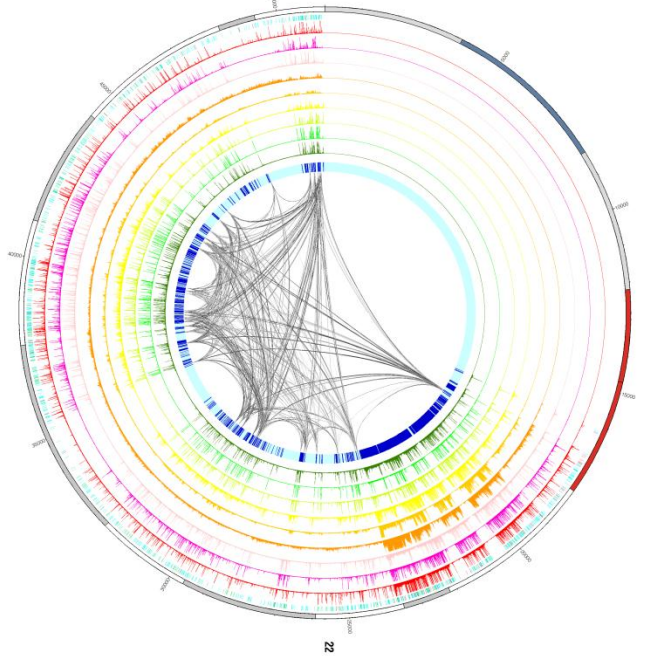

Supplement: Figure S13 — Identification of chromatin domains in K562 cells. Circos map of the whole-genome chromatin domains, associated CTCF-binding sites, DNaseI HS, and histone modifications from chromosome 1 to chromosome X, generated using the Circos software package. Chromatin domains were identified using HMMSeg. The outermost circle (circle 1) represents the chromosome band (scale in Kb). Circles 2 and 3 represent the CTCF peaks and tag density profile, respectively. Circle 4 represents the DNaseI HS profile. Circles 5–11 represent the histone modifications H3K27ac, H3K27me3, H3K36me3, H3K4me1, H3K4me2, H3K4me3, and H3K9ac, respectively. Circle 12 represents the euchromatin (medium blue) and heterochromatin (light cyan) domain. Intrachromosomal interactions are drawn in the innermost ring with color intensities (from white to gray) reflecting interaction strength (from low to high). (PDF) [file pone.0041374.s013.pdf]

**(A)**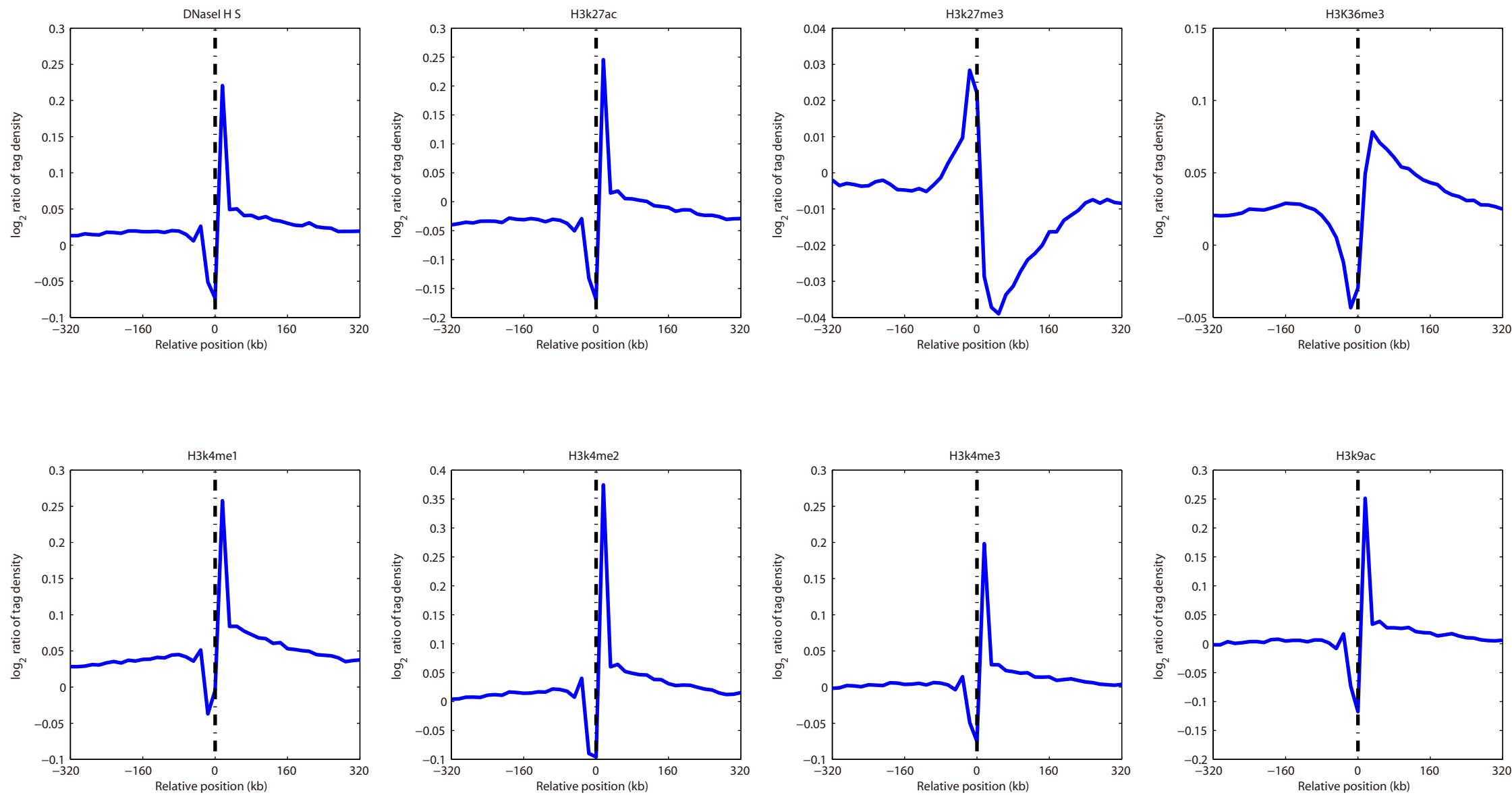**Figure S14**

**(B)**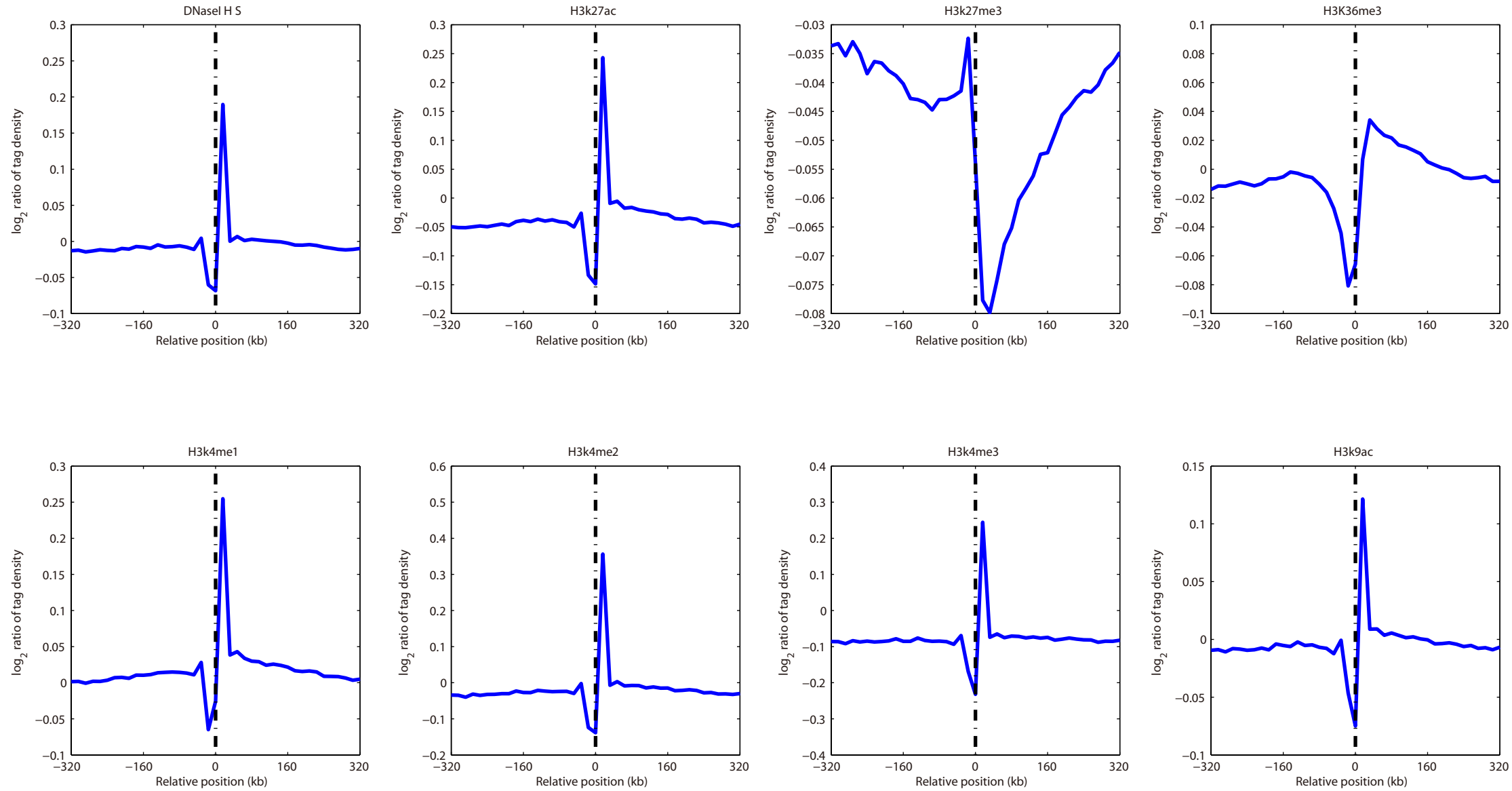

**(C)**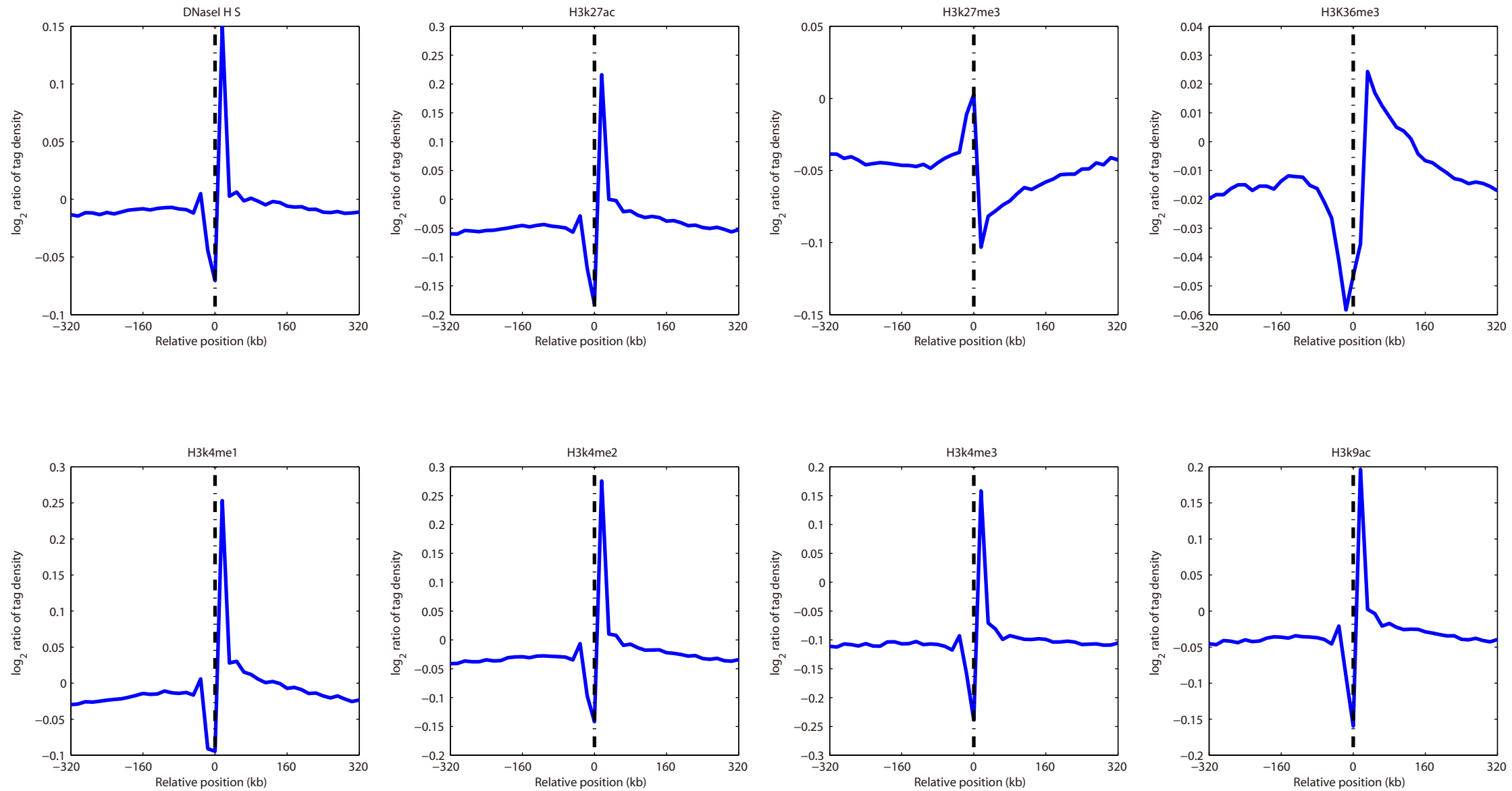

**(D)**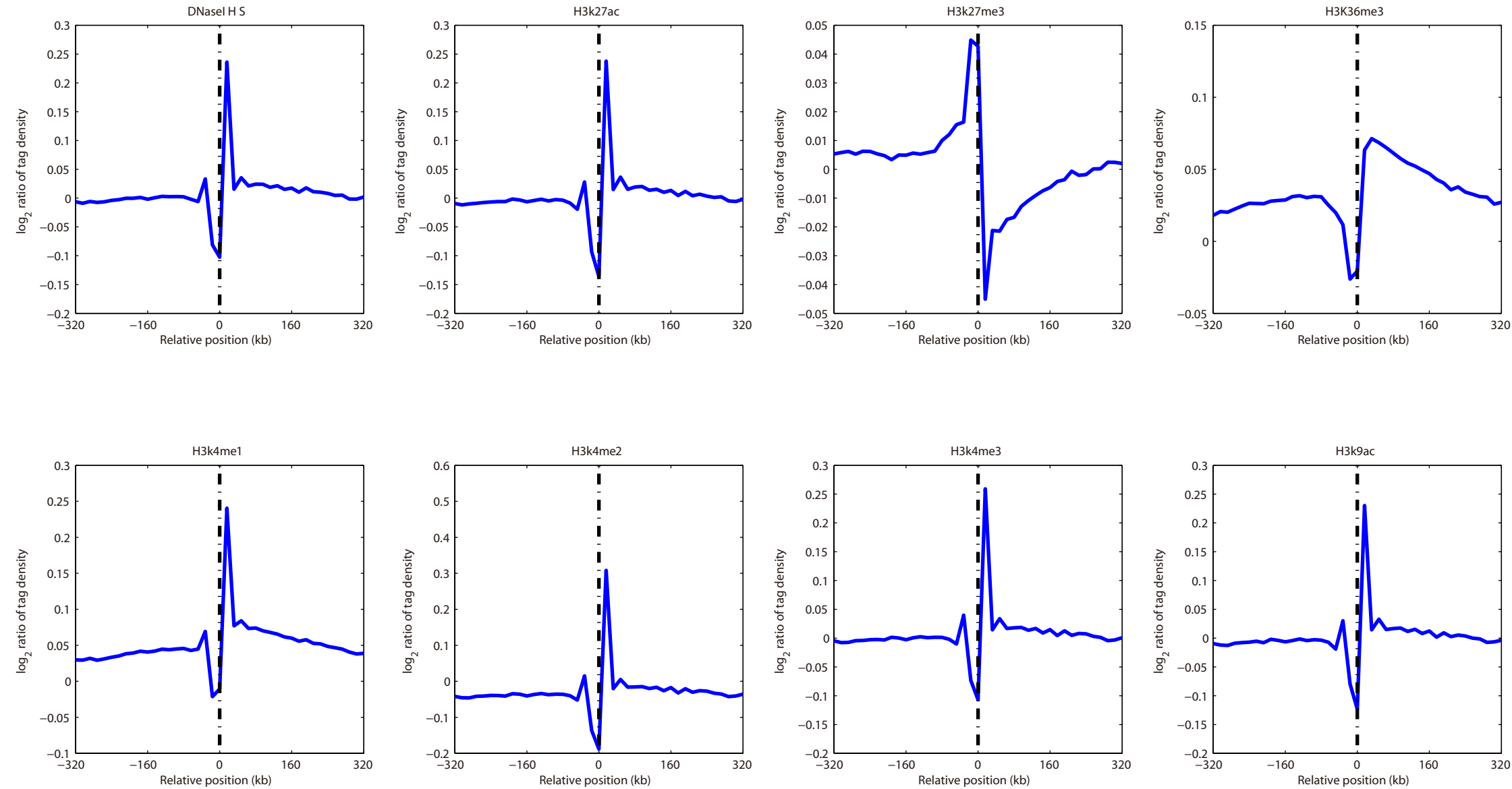

**(E)**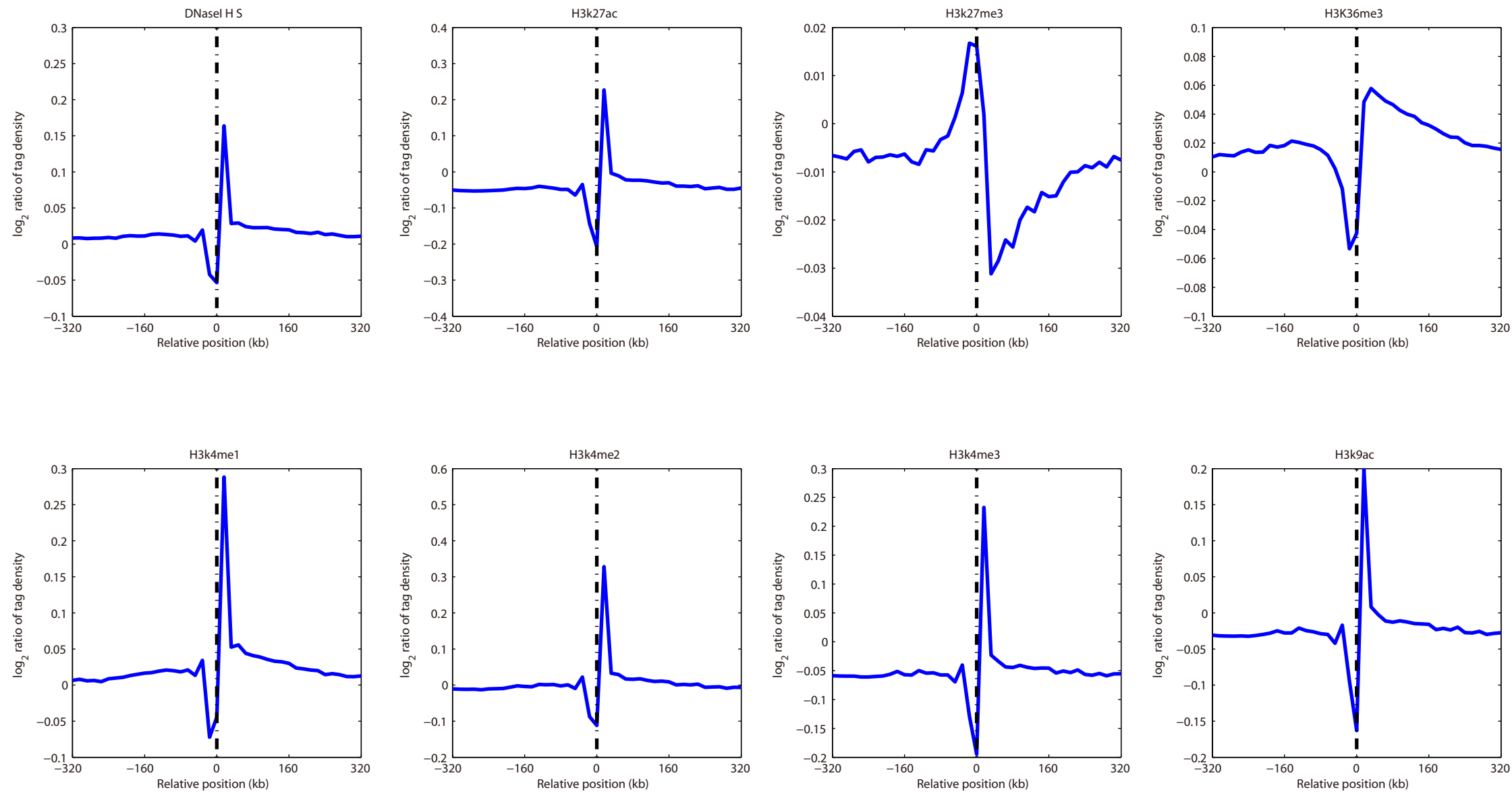

Supplement: Figure S14 — Chromatin features around the chromatin borders across cell types. Profiles of genomic and chromatin features around chromatin borders of GM12878 (A), HMEC (B), HUVEC (C), K562 (D), and NHEK (E) cell types. Log2 ratio profiles of aligned chromatin border regions (all 2,688 borders in K562 cells; left and mirrored right border regions combined) are shown for DNaseI HS, H3K4me1, H3K4me2, H3K4me3, H3K9ac, H3K27ac, H3K36me3, H4K20me1, and H3K27me3. To align chromatin borders, genome-wide positions of all analyzed features were converted to coordinates relative to the nearest border. The area to the left of the dash-dot line and all negative coordinates represent heterochromatin domains; the area to the right of the dash-dot line and all positive coordinates represent euchromatin domains. Blue lines show moving-window averages with window sizes of 16 kb. (PDF) [file pone.0041374.s014.pdf]

Figure S15

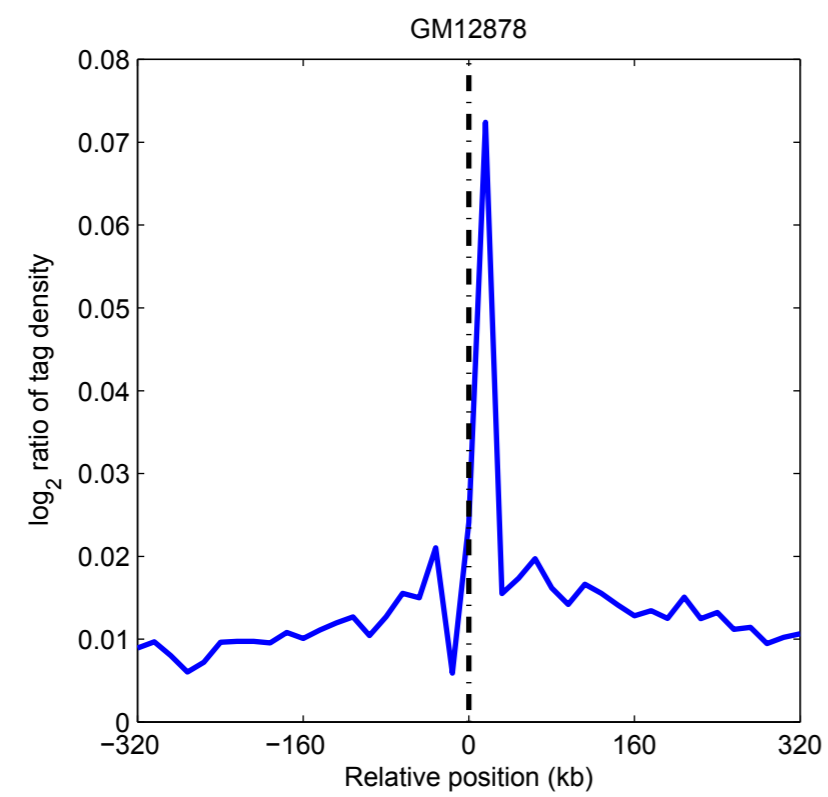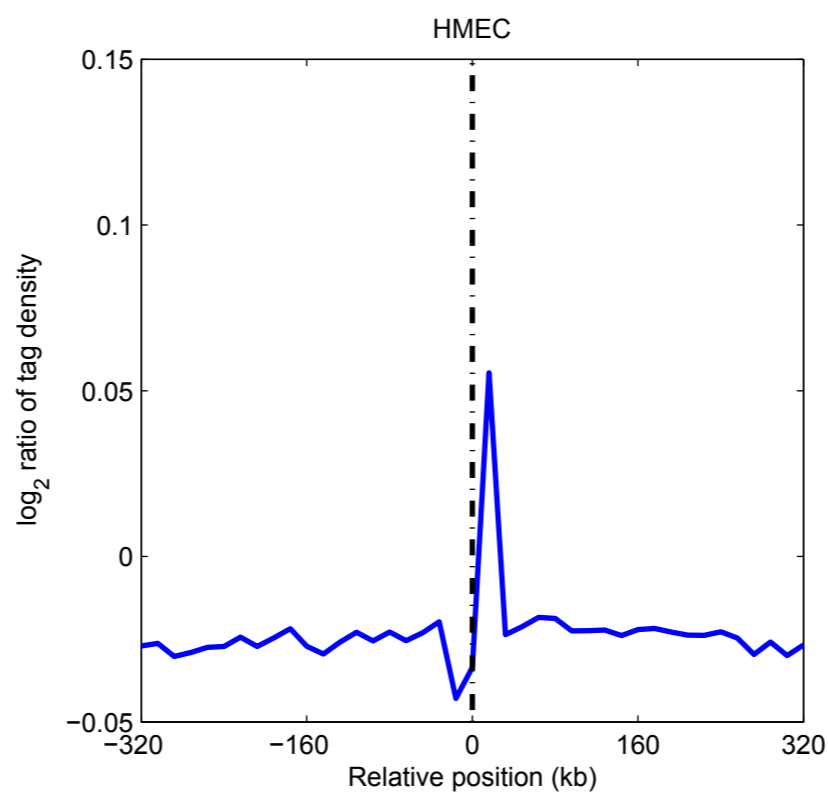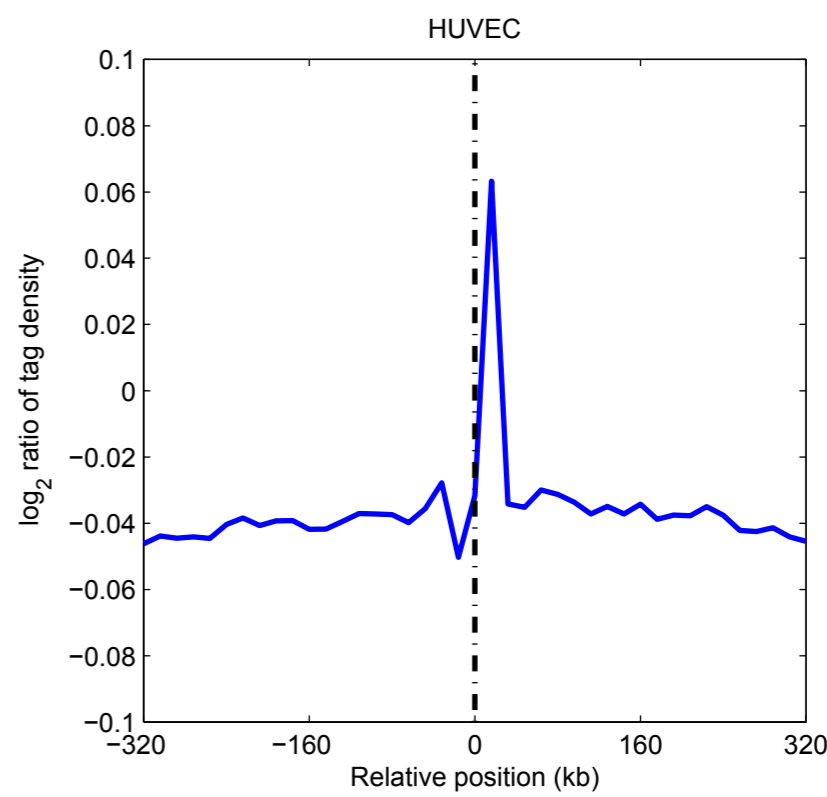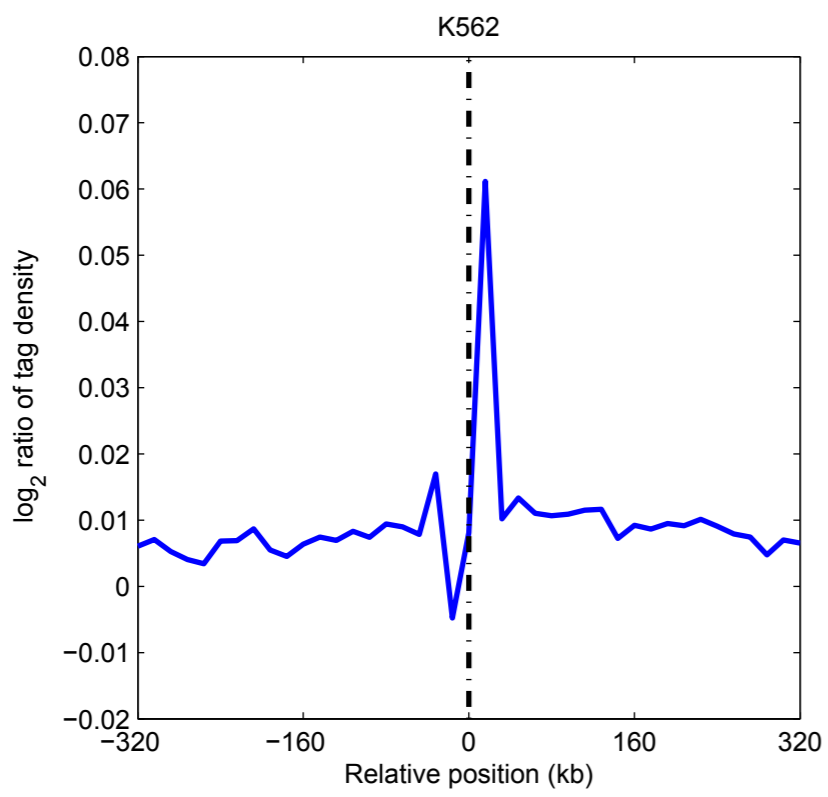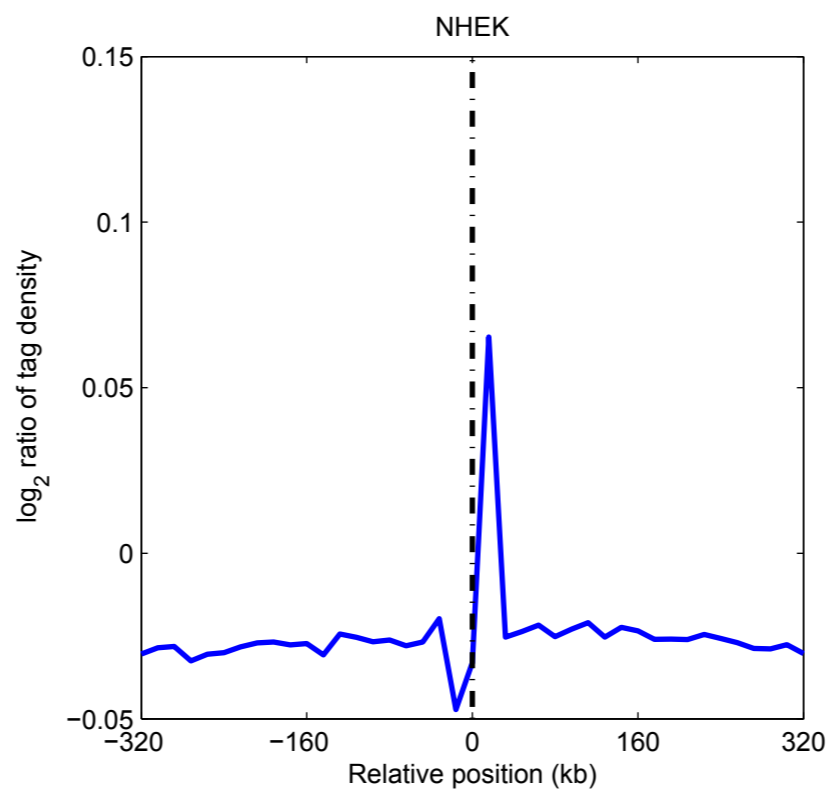

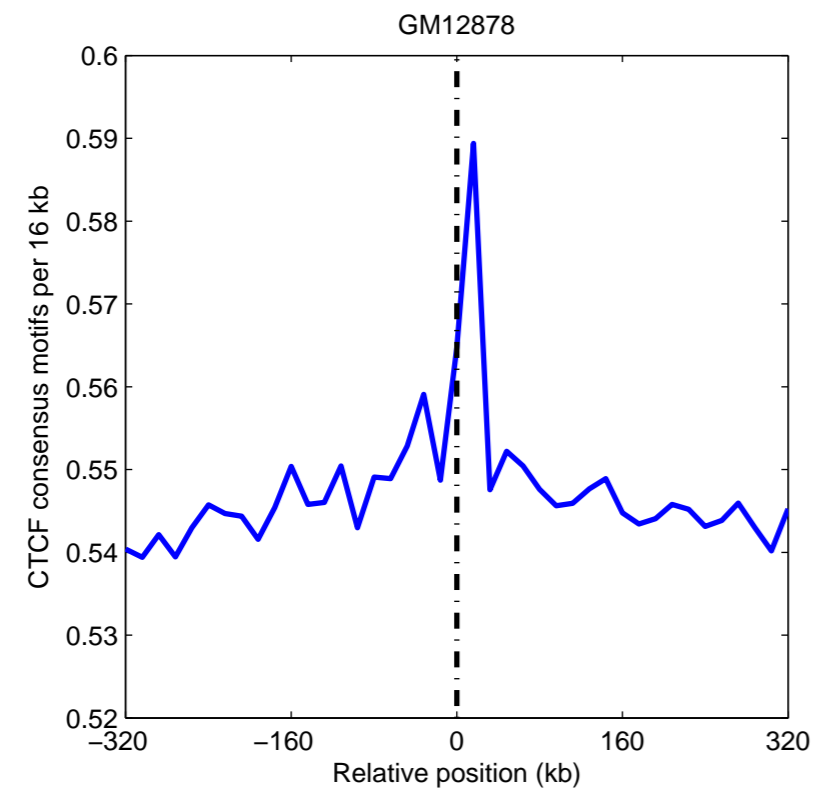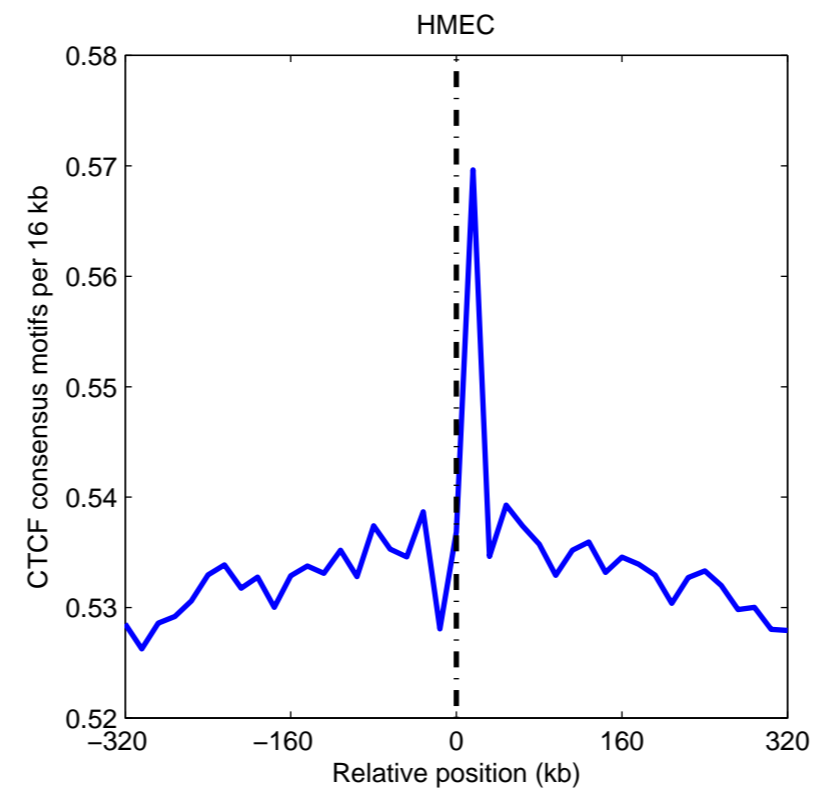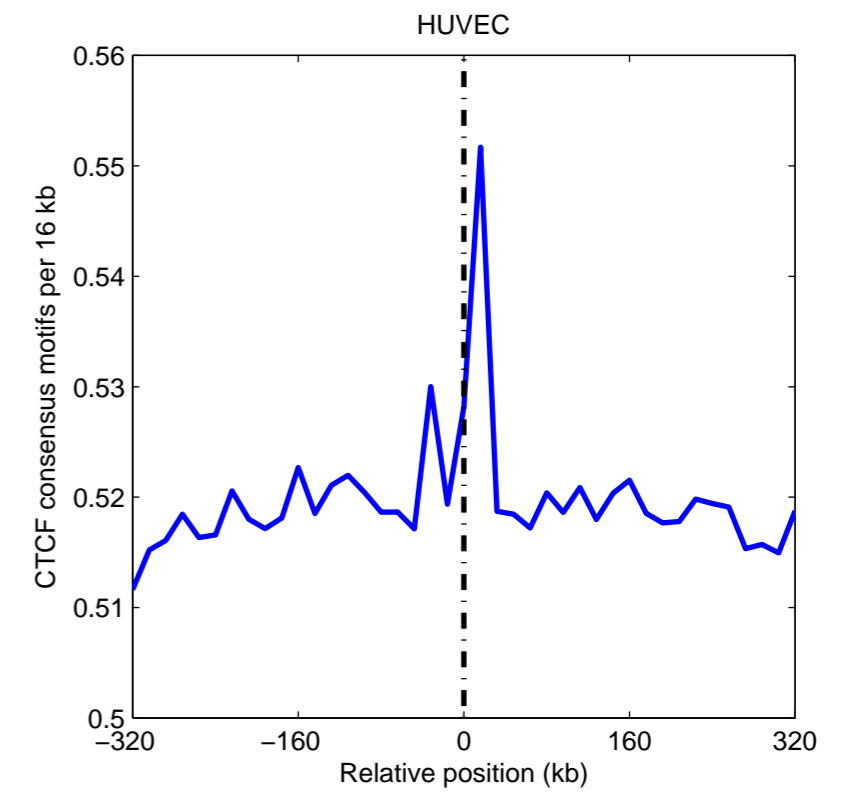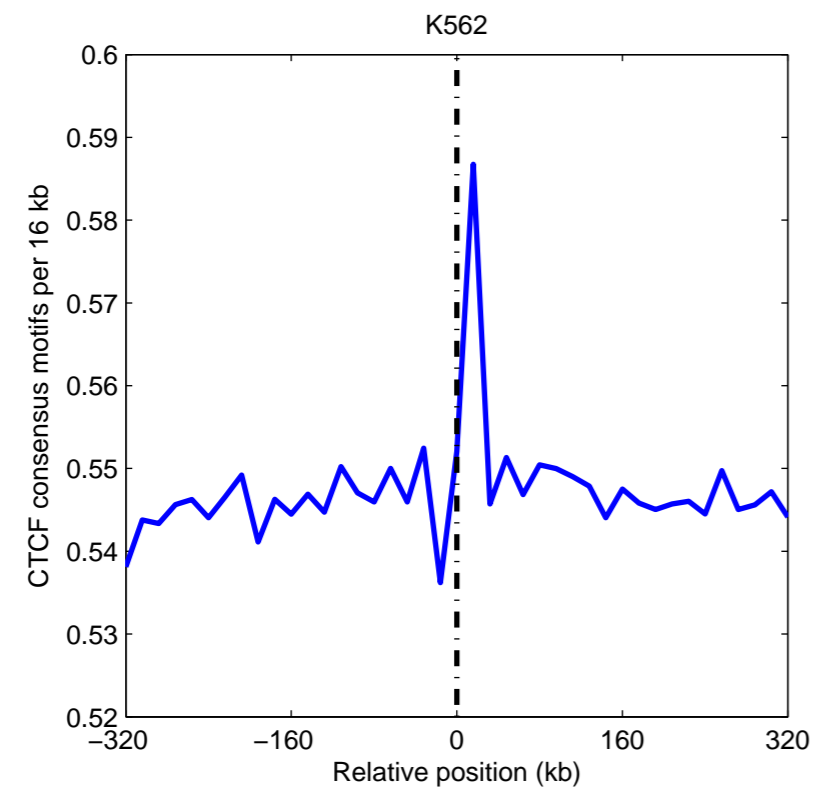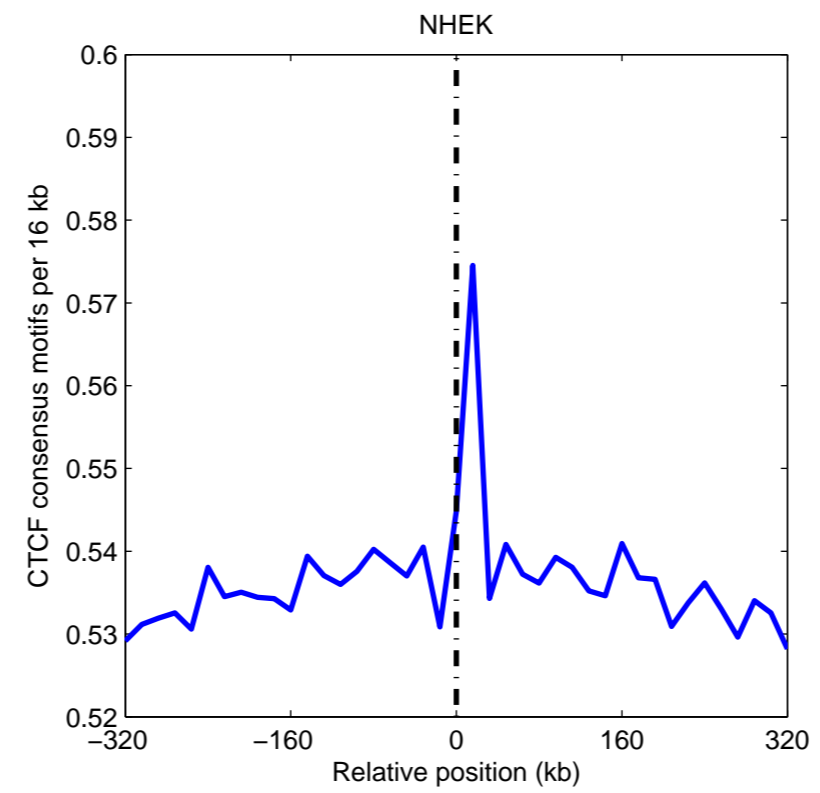

Supplement: Figure S15 — Profiles of CTCF features around chromatin borders across cell types. (A) CTCF profiles at chromatin boundary. Tag density of CTCF centered on chromatin domain boundaries, and extended 320 kb upstream and downstream of the boundary at 16 kb resolution. Plotted on the y-axis is the normalized tag density and on the x-axis is distance from the chromatin boundary. (B) CTCF consensus motifs are enriched on the chromatin boundary. Number profiles of CTCF consensus motifs centered on boundaries of chromatin domains, and extended 320 kb upstream and downstream of the boundary at 16 kb resolution. Plotted on the y-axis is the normalized number of CTCF-binding sites and on the x-axis is distance from the chromatin boundary. (PDF) [file pone.0041374.s015.pdf]

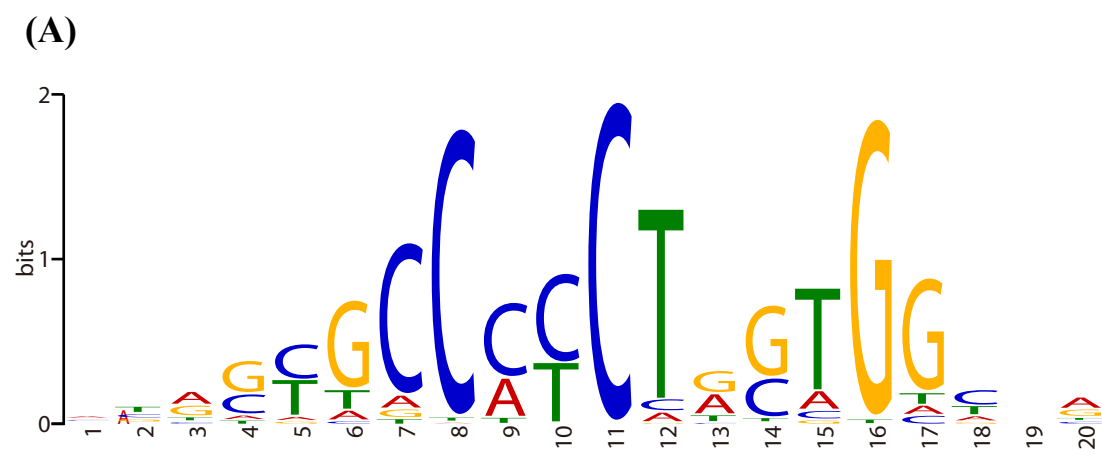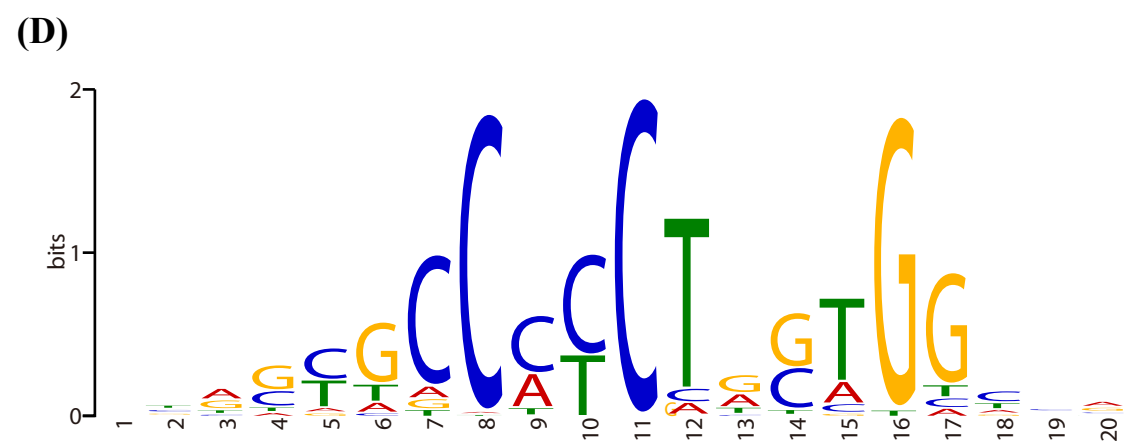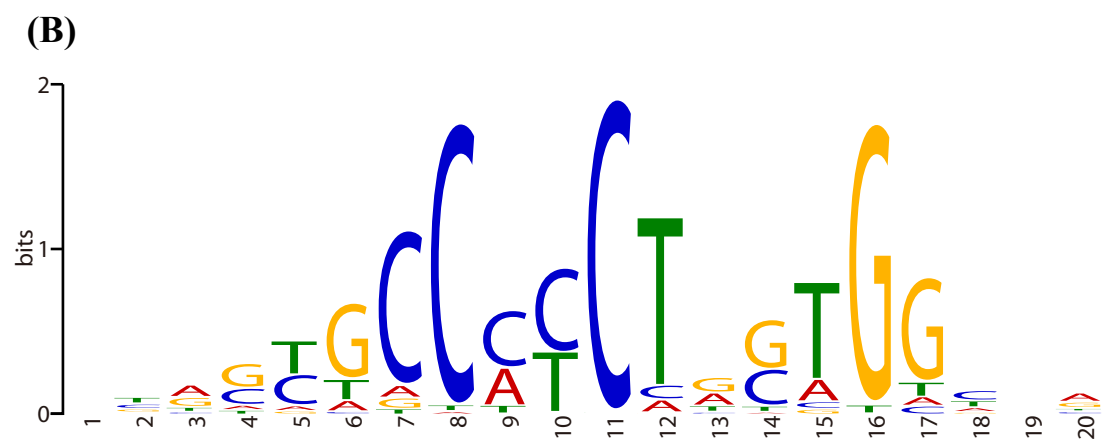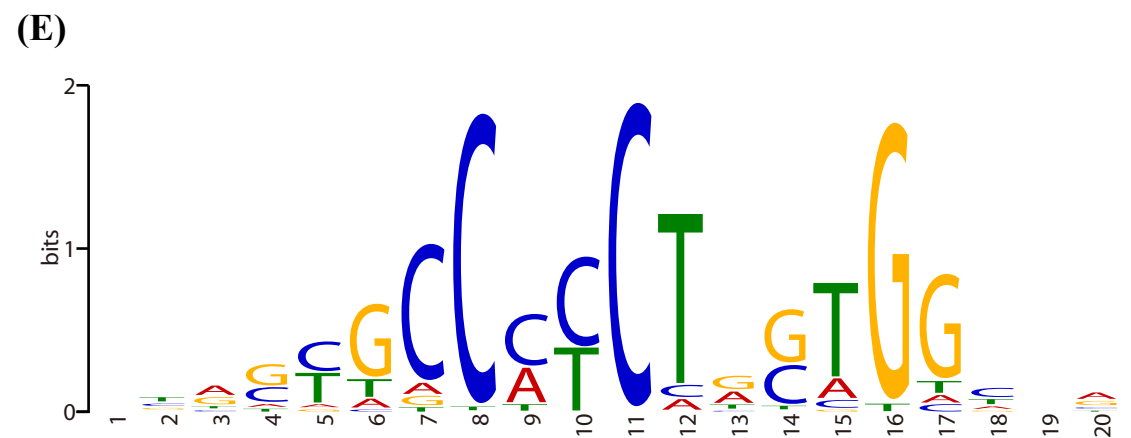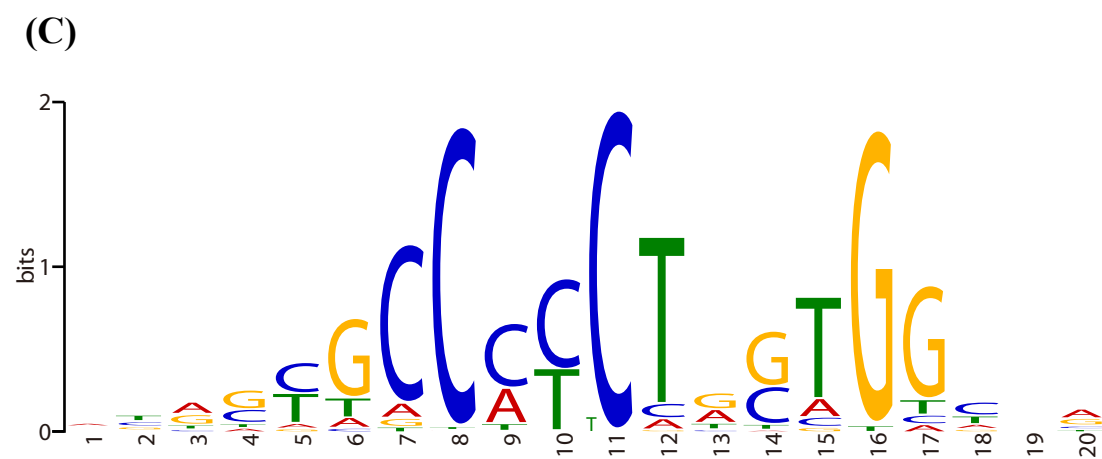

**Figure S16**

Supplement: Figure S16 — CTCF consensus motifs identified within barrier CTCF-binding sites across five cell types. Significantly enriched CTCF-consensus motifs identified within barrier CTCF-binding sites of (A) GM12878, (B) HMEC, (C) HUVEC, (D) K562, and (E) NHEK cells are graphically depicted using WebLogo. (PDF) [file pone.0041374.s016.pdf]

**(A)**

**K562**

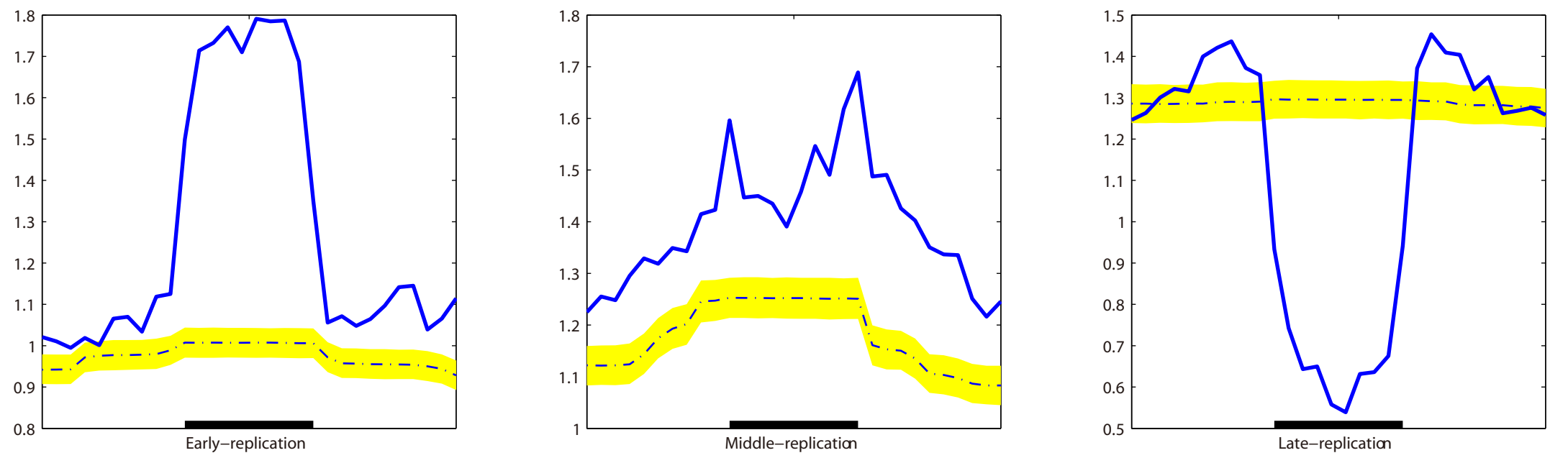

**(B)**

**GM12878**

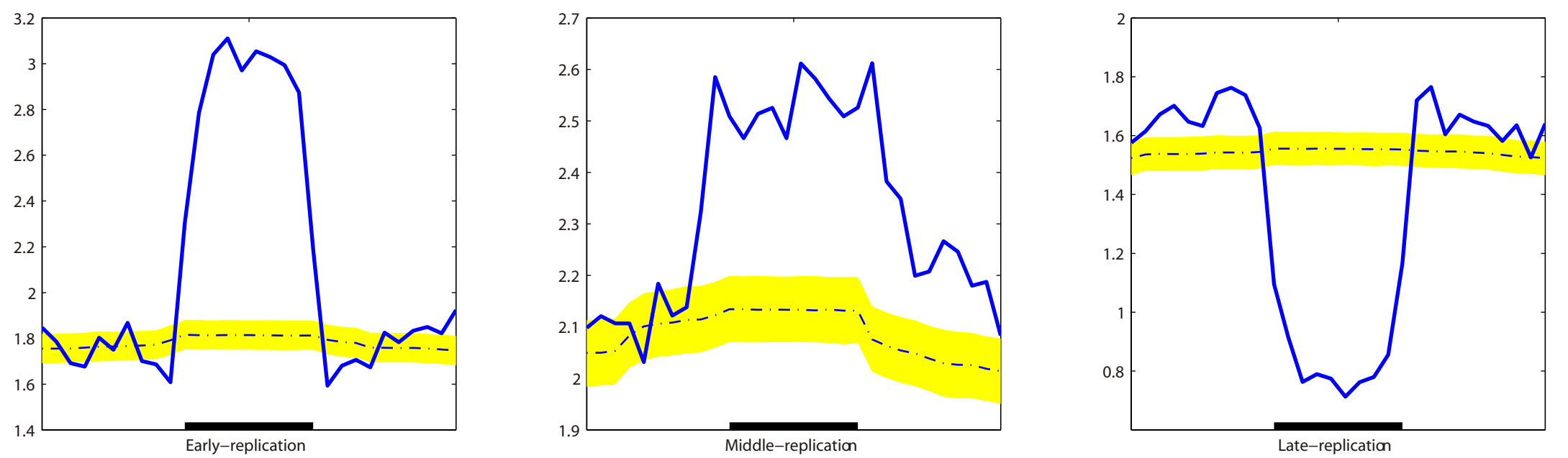

**Figure S17**

Supplement: Figure S17 — Cumulative number of CTCF-binding sites within replicating zones across cell types. Cumulative number of CTCF sites within replicating zones of K562 (A) and GM06990 (B) cell types. The cumulative normalized number of CTCF-binding sites within early-replicating zones (left), middle-replicating zones (middle) and late-replicating zones (right) were plotted for comparison of the densities of CTCF-binding sites and shuffled CTCF-binding sites within replicating zones. The intensity plots show the significantly different patterns of CTCF-binding sites and shuffled CTCF-binding sites. (PDF) [file pone.0041374.s017.pdf]

Figure S18

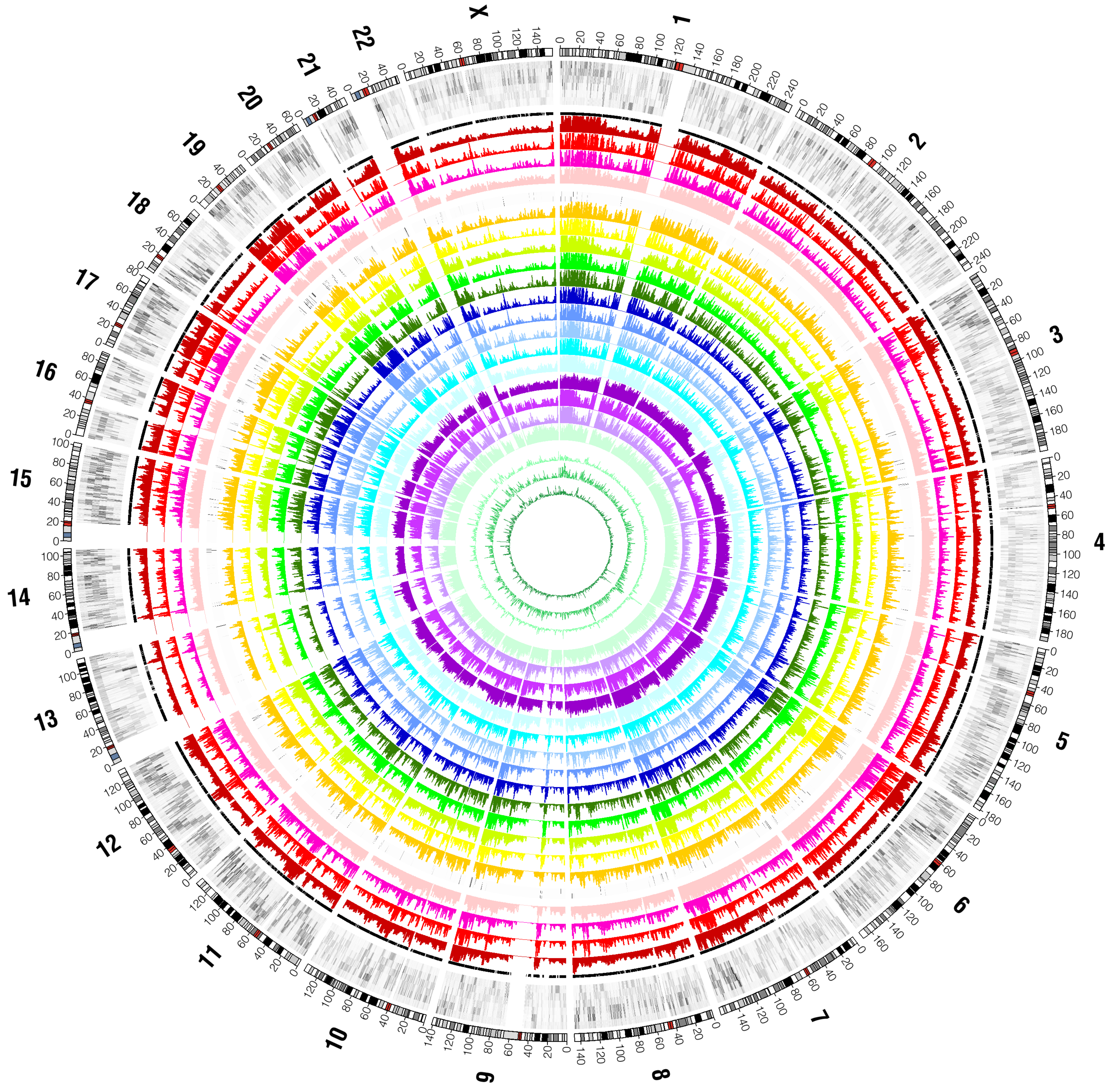

Supplement: Figure S18 — Raw data from the K562 cell line. Circos map of the whole-genome raw data from K562 cells used in this study, created with the Circos software package. The outermost circle (circle 1) represents the chromosome band (scale in Mb). Circles 2–7 represent the replicating time in G1, S1, S2, S3, S4, G2, respectively, with color intensities reflecting their interaction strength (from white to gray). Circles 8 and 9 represent the CTCF peaks and tag density profile, respectively. Circles 10–12 represent the tag density of the DNaseI DGF, DNaseI HS, and FAIRE profiles, respectively. Circle 13 represents the tag density of DNA methylation. Circles 14–26 represent the histone modifications H2A.Z, H3K27ac, H3K27me3, H3K36me3, H3K4me1, H3K4me2, H3K4me3, H3K79me2, H3K9ac, H3K9me1, H3K9me3, H4K20me1, and Pol2, respectively. Circle 27 represents the phastCons 46-way conservation. Circle 28 and 29 represent enhancers and promoters, respectively. Circle 30 represents gene density. (PDF) [file pone.0041374.s018.pdf]
