# Supplementary material for: Comprehensive Identification and Annotation of Cell Type-Specific and Ubiquitous CTCF-Binding Sites in the Human Genome
Source: PLoS One. 2012 Jul 19;7(7):e41374. doi: 10.1371/journal.pone.0041374 (PMC3400636; doi:10.1371/journal.pone.0041374)
Supplement: Table S7 — Over-represented motifs within ubiquitous and cell type-specific CTCF-binding sites across 38 cell types. (DOCX) [file pone.0041374.s025.docx]

**Table S7. Overrepresented motifs within ubiquitous and cell type specific CTCF binding sites across 38 cell types**

| Motif | 1 | 2 | 3 | 4 | 5 |
| --- | --- | --- | --- | --- | --- |
| U | 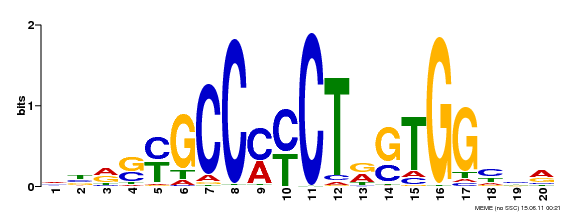  E score = 2.3e-30510 | 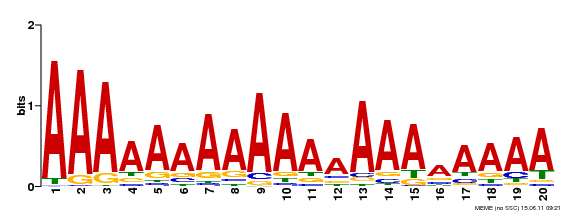  E score = 5.1e-2426 | 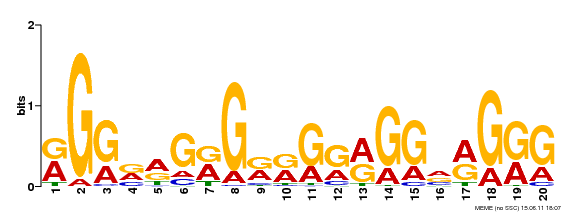  E score = 1.6e-977 | 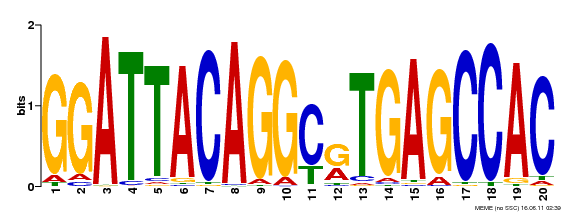  E score = 7.0e-373 | 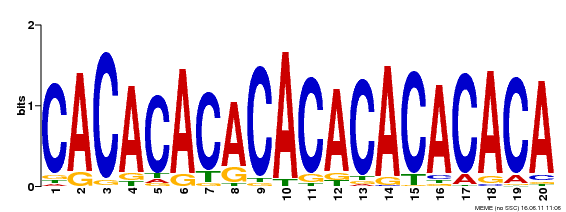  E score = 6.2e-371 |
| C1 | 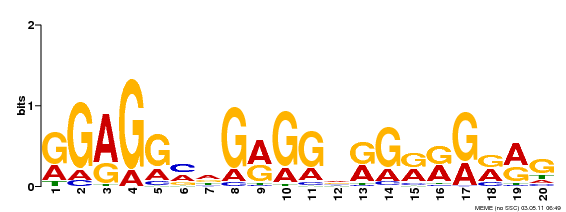  E score = 8.5e-642 | 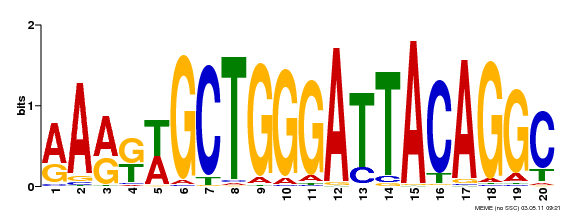  E score = 9.2e-398 | 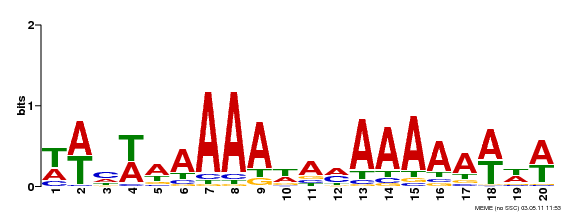  E score = 5.7e-384 | 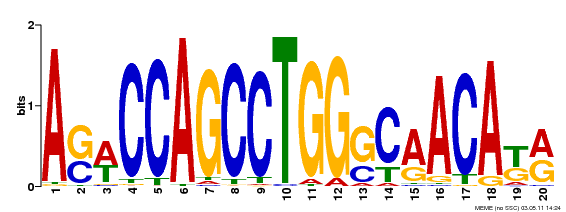  E score = 6.7e-244 | 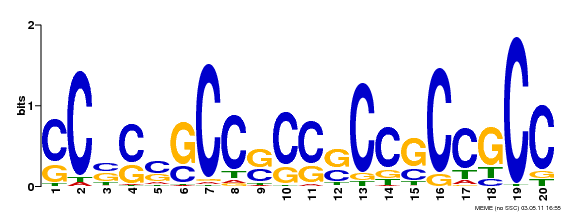  E score = 2.4e-196 |
| C2 | 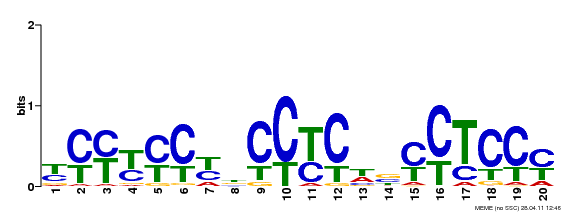  E score = 5.1e-360 | 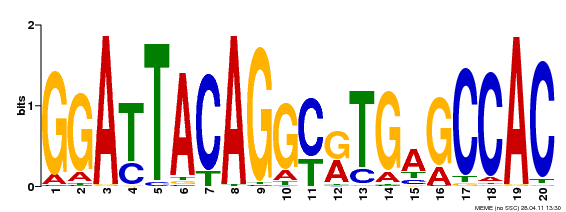  E score = 8.4e-160 | 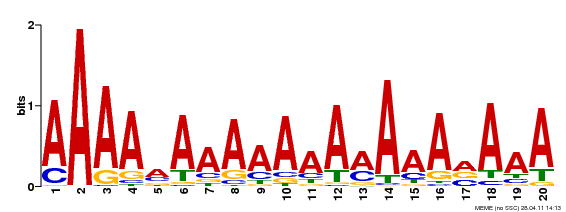  E score = 5.0e-136 | 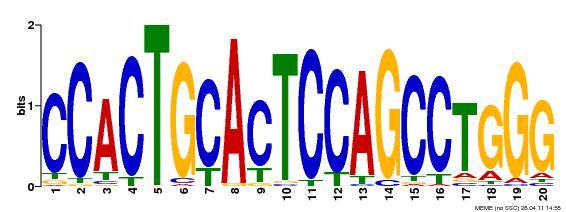  E score = 2.0e-131 | 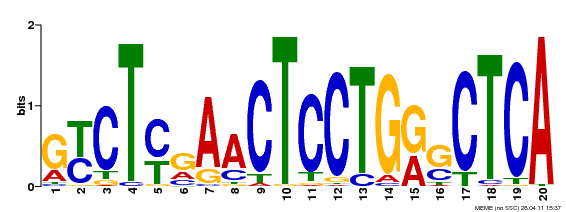  E score = 1.1e-110 |
| C3 | 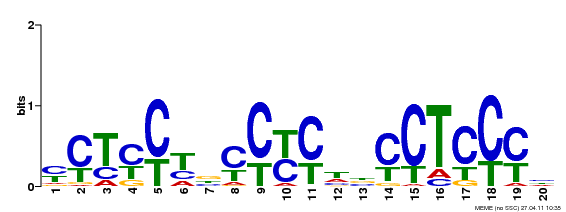  E score = 1.2e-277 | 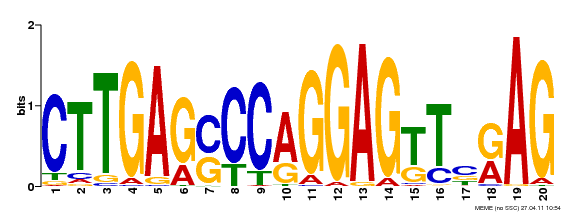  E score = 1.1e-136 | 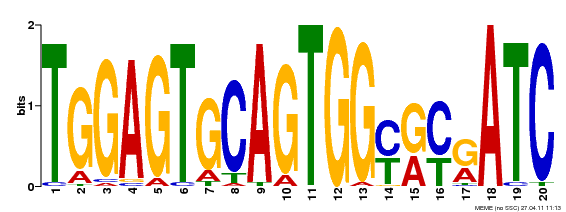  E score = 6.8e-096 | 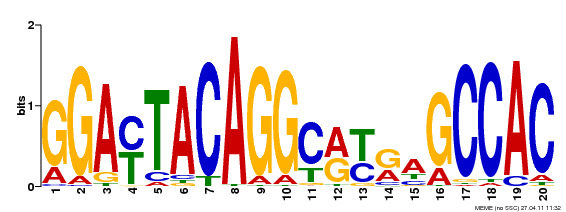  E score = 1.3e-116 | 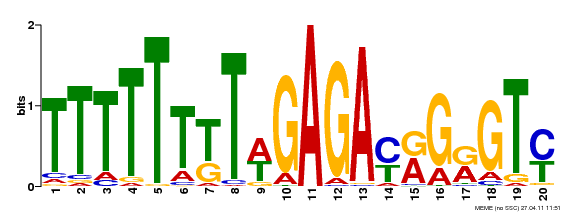  E score = 1.0e-108 |
| C4 | 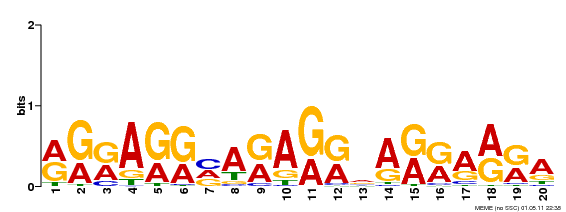  E score = 1.8e-367 | 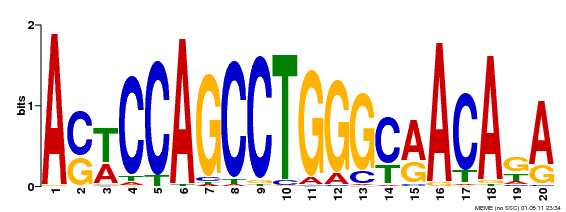  E score = 6.7e-267 | 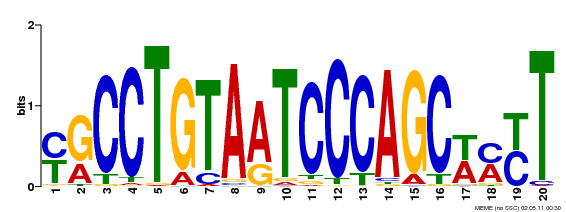  E score = 7.0e-228 | 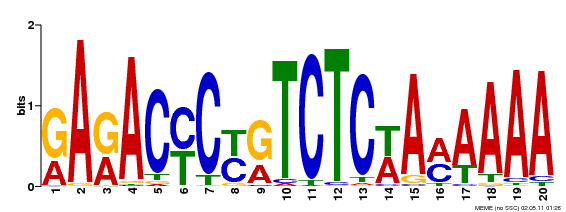  E score = 2.8e-157 | 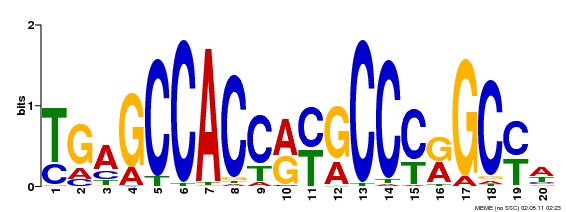  E score = 9.3e-147 |
| C5 | 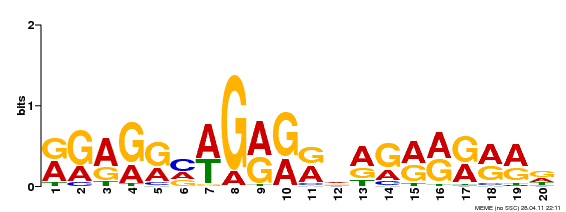  E score = 1.4e-367 | 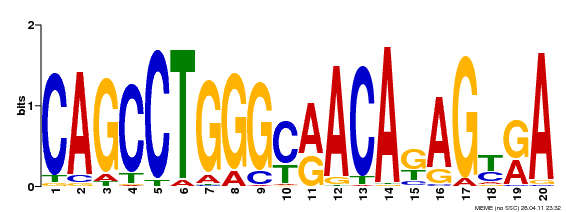  E score = 1.2e-143 | 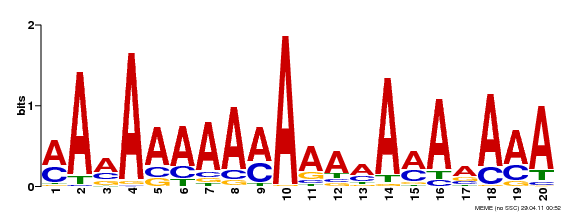  E score = 8.9e-161 | 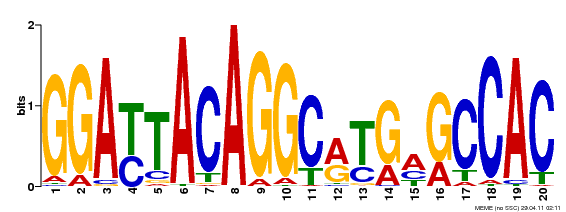  E score = 3.6e-117 | 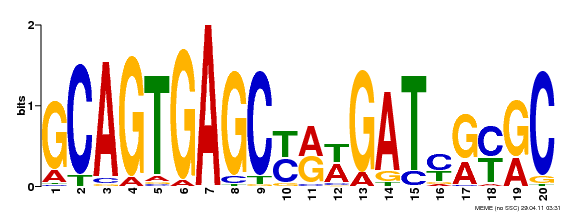  E score = 8.8e-068 |
| C6 | 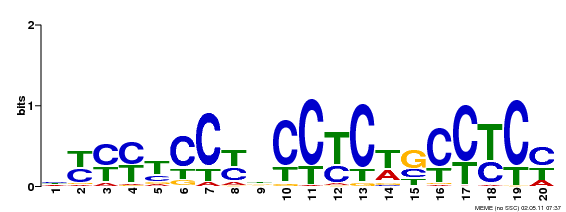  E score = 2.1e-308 | 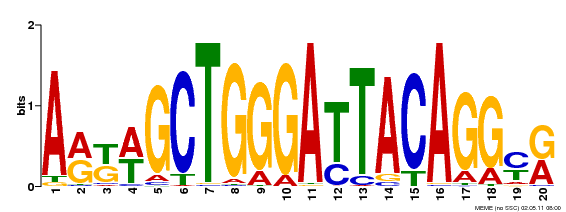  E score = 3.9e-172 | 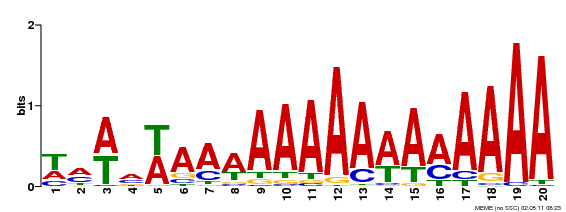  E score = 6.7e-206 | 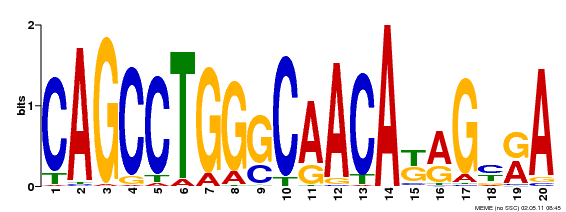  E score = 1.7e-138 | 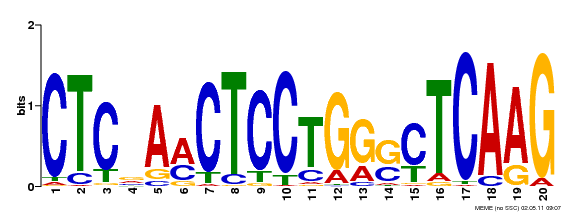  E score = 5.1e-118 |
| C7 | 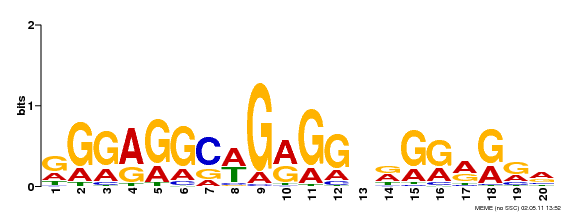  E score = 2.9e-506 | 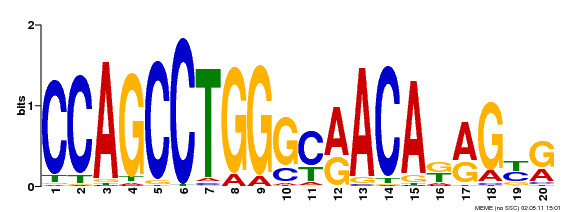  E score = 7.6e-353 | 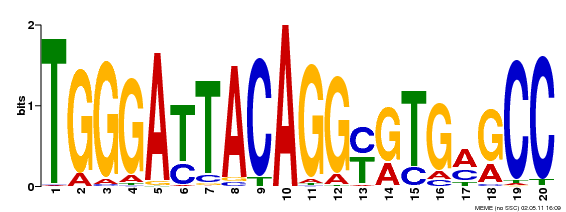  E score = 8.1e-320 | 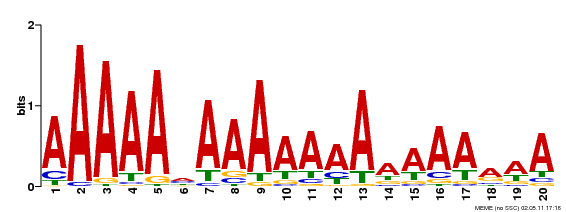  E score = 1.7e-307 | 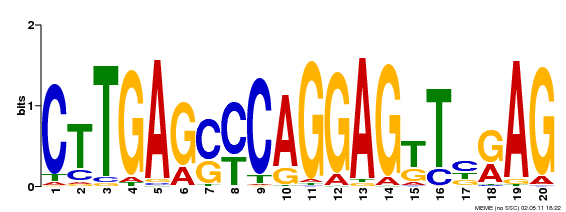  E score = 3.9e-227 |
| C8 | 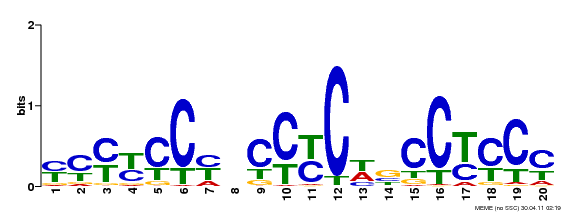  E score = 2.7e-782 | 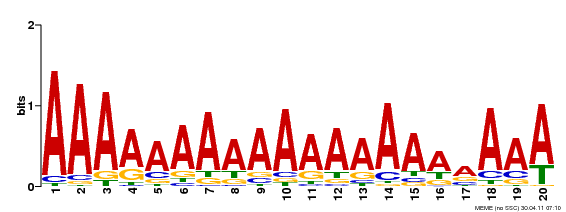  E score = 2.5e-568 | 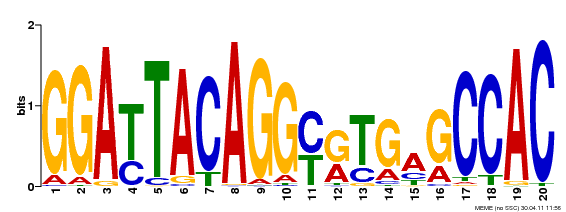  E score = 4.1e-328 | 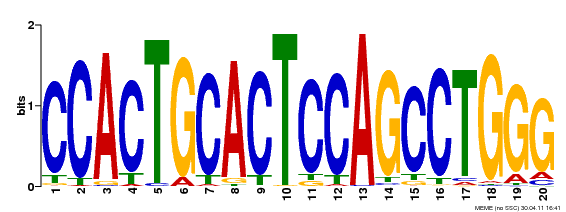  E score = 3.8e-311 | 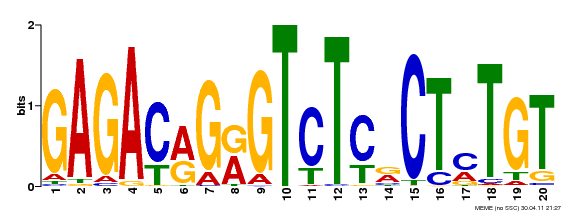  E score = 8.9e-245 |
| C9 | 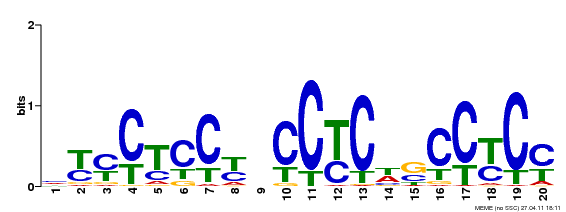  E score = 2.4e-720 | 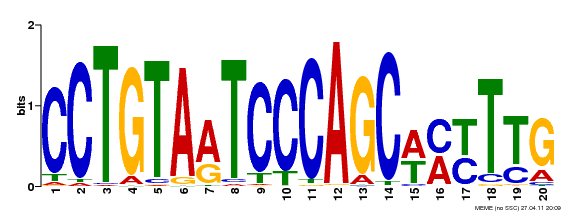  E score = 4.9e-546 | 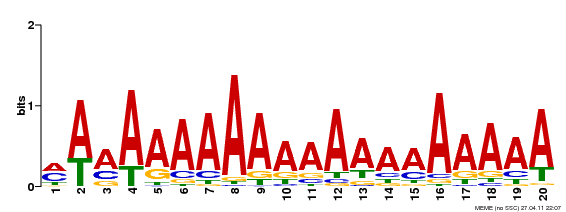  E score = 1.3e-541 | 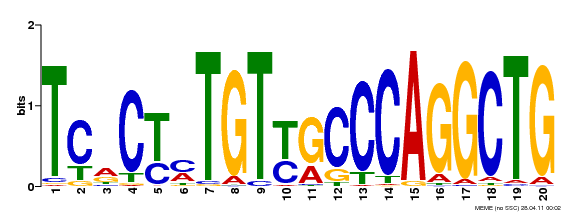  E score = 1.9e-536 | 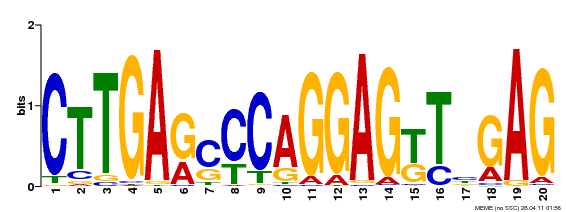  E score = 1.7e-356 |
| C10 | 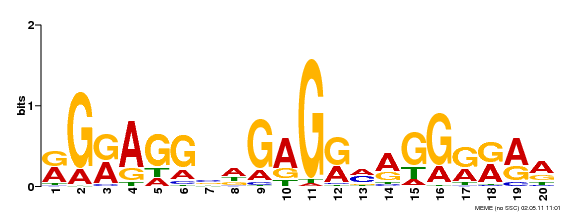  E score = 6.0e-110 | 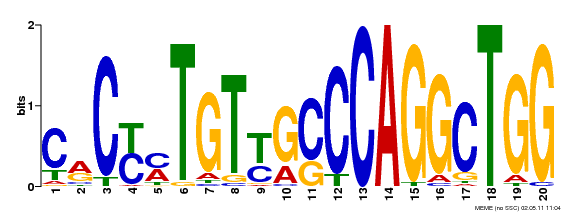  E score = 6.6e-067 | 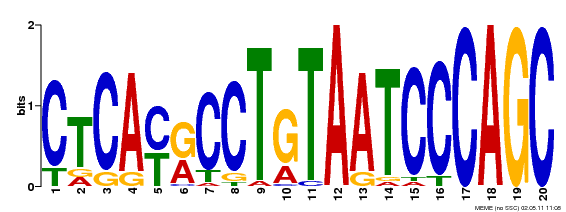  E score = 1.6e-063 | 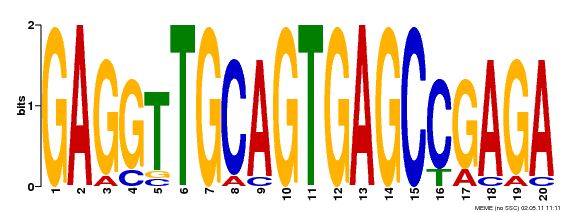  E score = 2.0e-037 | 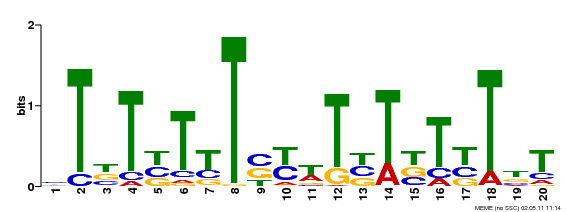  E score = 1.1e-025 |
| C11 | 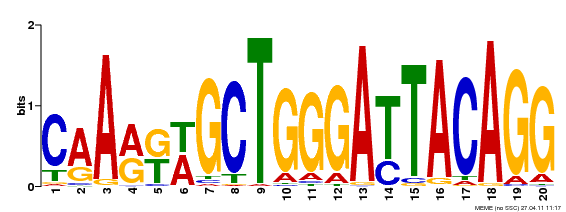  E score = 9.0e-432 | 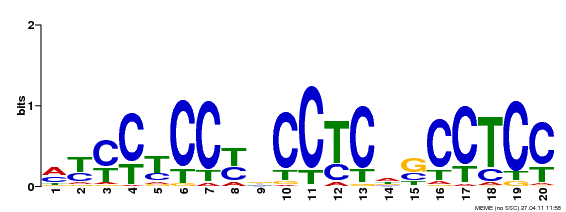  E score = 2.6e-378 | 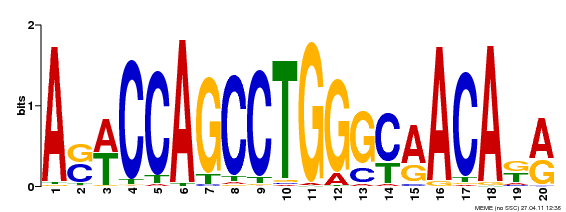  E score = 1.2e-330 | 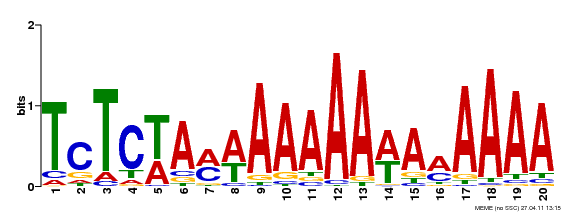  E score = 4.5e-322 | 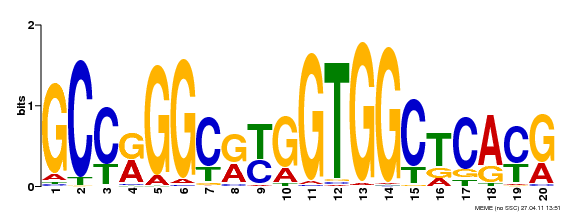  E score = 3.6e-251 |
| C12 | 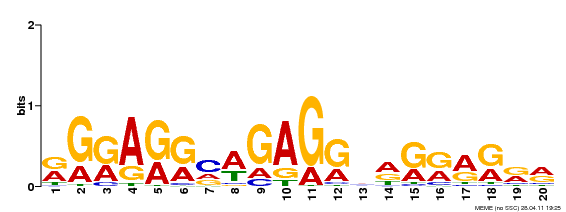  E score = 1.2e-753 | 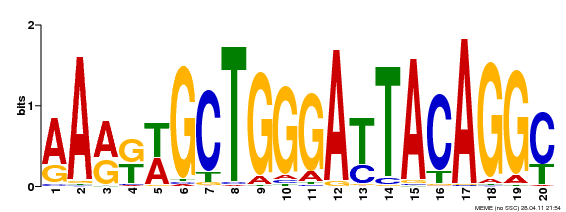  E score = 7.0e-589 | 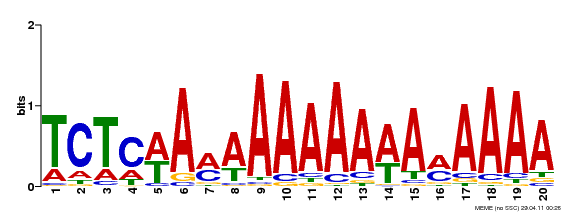  E score = 2.9e-464 | 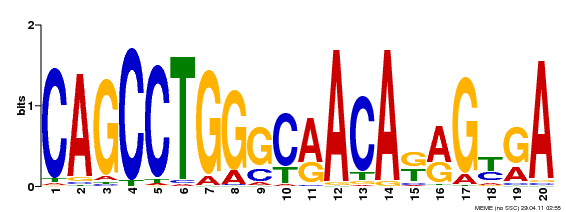  E score = 1.5e-490 | 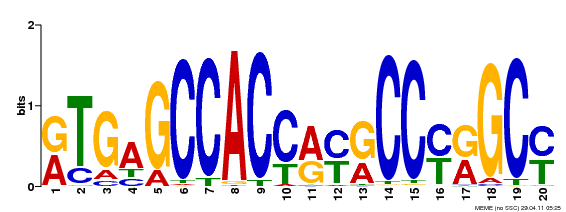  E score = 7.7e-403 |
| C13 | 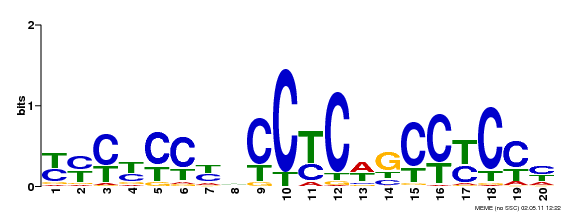  E score = 8.0e-338 | 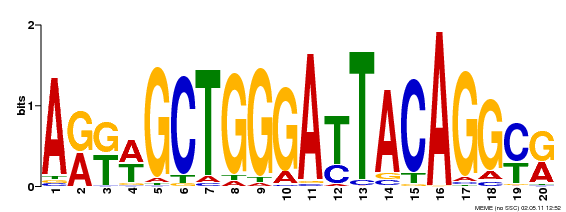  E score = 2.3e-330 | 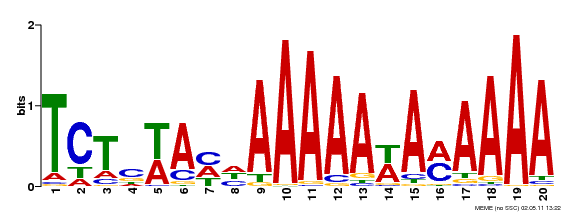  E score = 1.2e-267 | 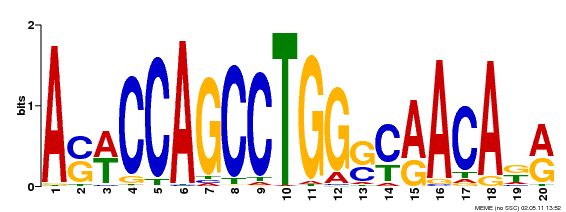  E score = 6.4e-227 | 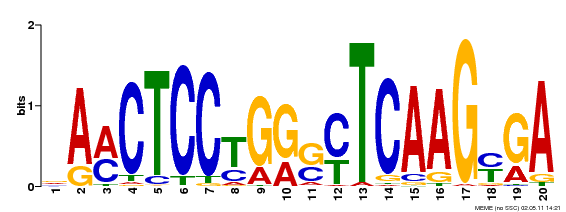  E score = 9.2e-165 |
| C14 | 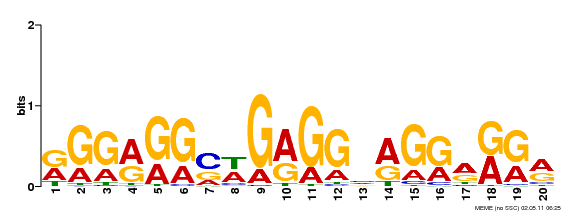  E score = 1.6e-470 | 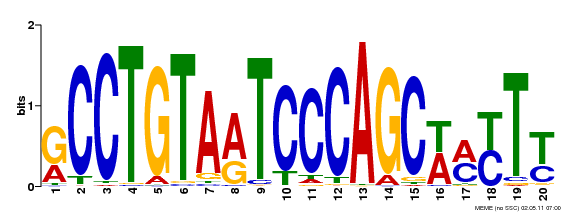  E score = 6.7e-373 | 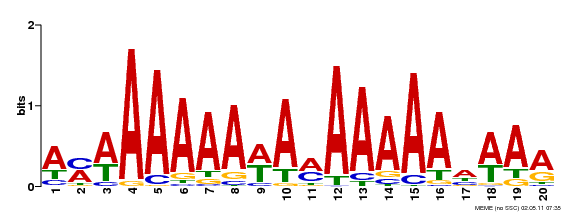  E score = 7.1e-275 | 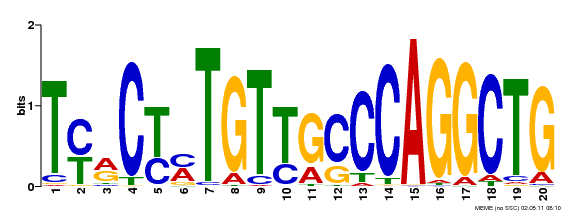  E score = 7.3e-264 | 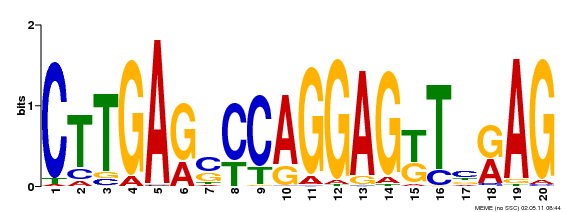  E score = 3.0e-213 |
| C15 | 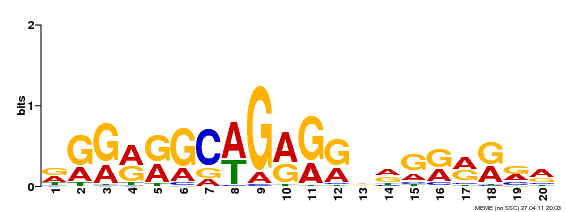  E score = 1.0e-438 | 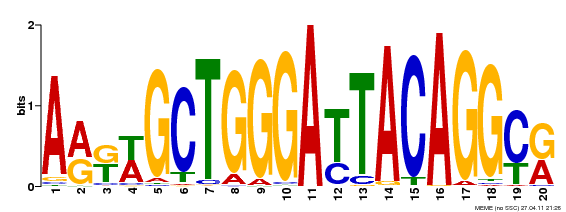  E score = 7.8e-316 | 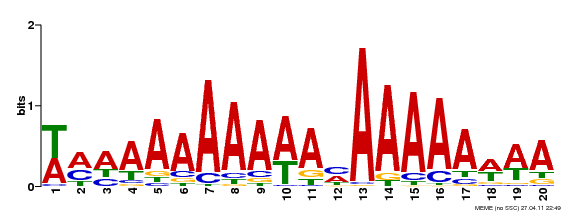  E score = 3.2e-284 | 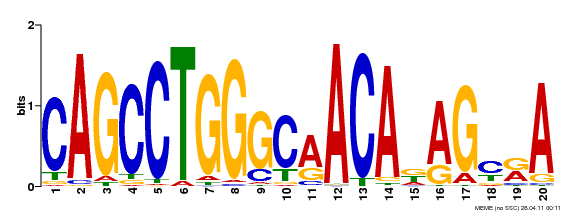  E score = 1.5e-242 | 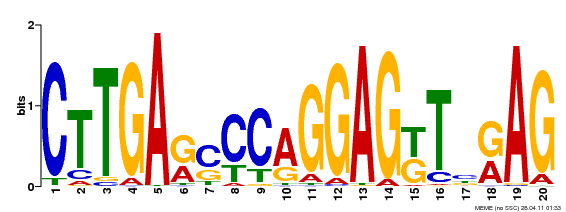  E score = 3.0e-184 |
| C16 | 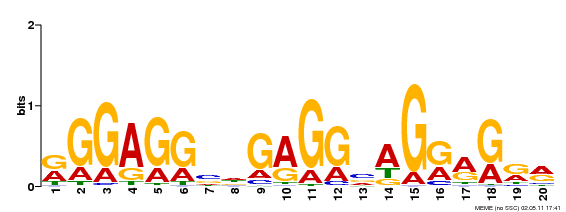  E score = 1.5e-312 | 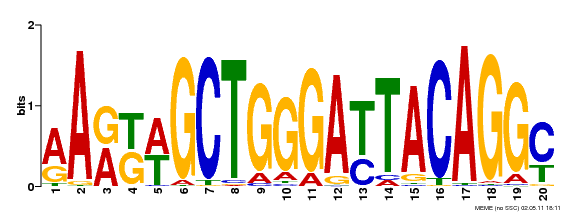  E score = 4.8e-247 | 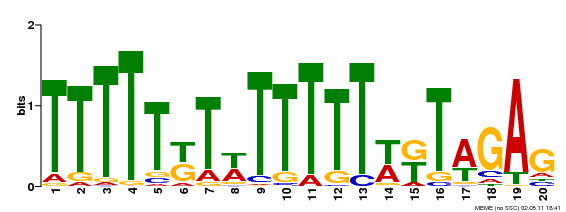  E score = 9.3e-210 | 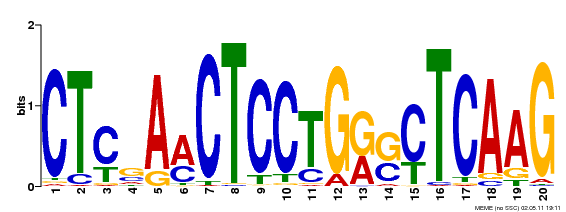  E score = 1.7e-162 | 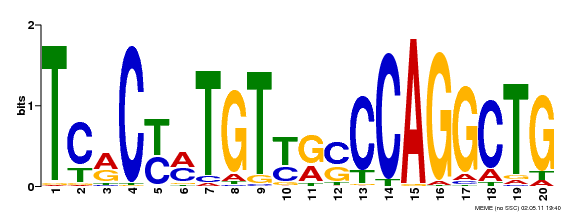  E score = 2.0e-165 |
| C17 | 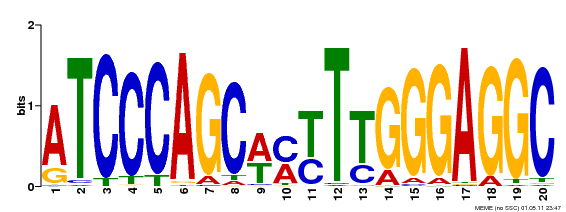  E score = 1.7e-663 | 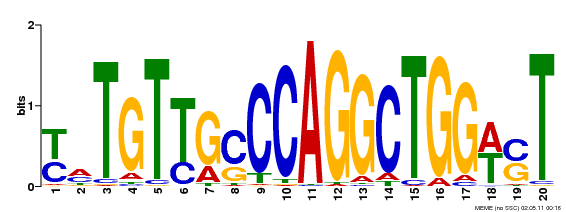  E score = 6.3e-518 | 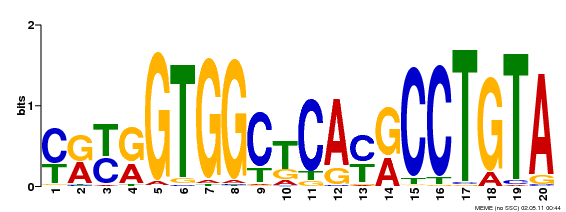  E score = 5.4e-497 | 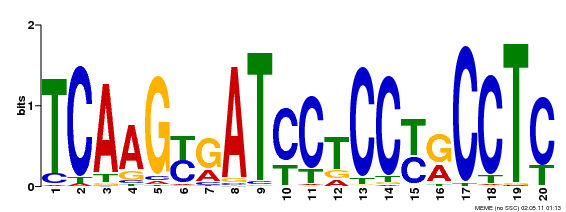  E score = 3.4e-492 | 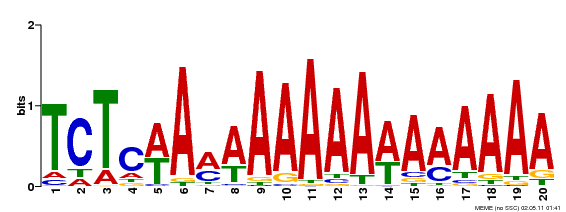  E score = 8.1e-451 |
| C18 | 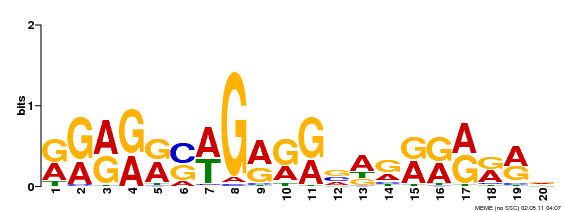  E score = 4.4e-189 | 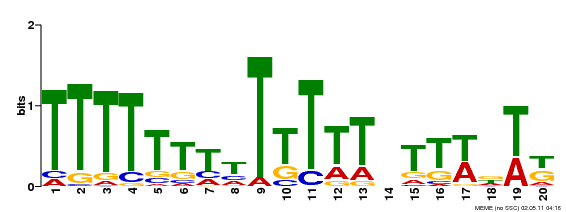  E score = 2.1e-076 | 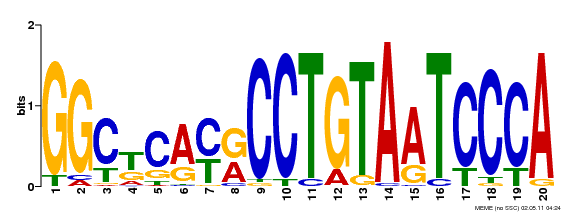  E score = 3.9e-064 | 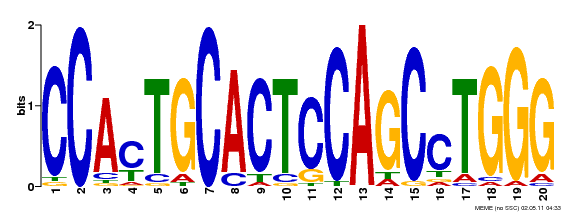  E score = 5.5e-062 | 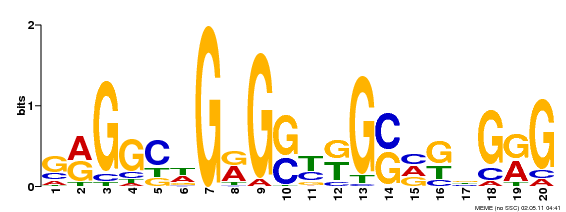  E score = 1.4e-052 |
| C19 | 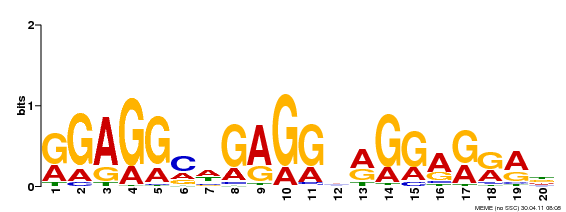  E score = 2.7e-1227 | 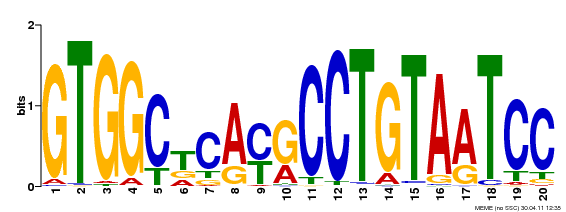  E score = 1.2e-943 | 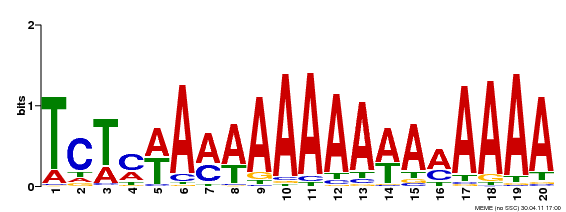  E score = 1.7e-988 | 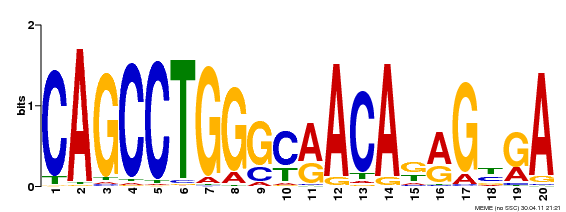  E score = 1.9e-828 | 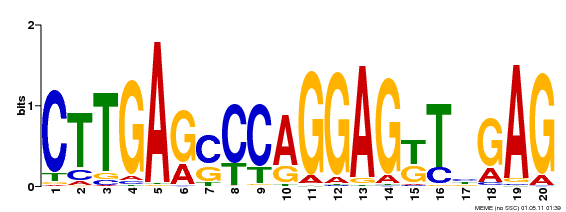  E score = 2.0e-654 |
| C20 | E score = 3.0e-277 | E score = 6.9e-222 | E score = 3.1e-172 | E score = 6.3e-183 | E score = 7.6e-125 |
| C21 | E score = 4.6e-1300 | E score = 7.0e-924 | E score = 9.9e-835 | E score = 5.3e-697 | E score = 2.4e-618 |
| C22 | E score = 3.7e-576 | E score = 2.8e-406 | E score = 6.9e-330 | E score = 7.3e-291 | E score = 4.4e-274 |
| C23 | E score = 3.5e-1315 | E score = 7.9e-808 | E score = 1.6e-778 | E score = 1.4e-742 | E score = 1.4e-934 |
| C24 | E score = 9.0e-423 | E score = 2.3e-309 | E score = 4.7e-332 | E score = 8.7e-293 | E score = 4.5e-251 |
| C25 | E score = 2.4e-1691 | E score = 2.3e-1047 | E score = 2.3e-1173 | E score = 1.1e-1026 | E score = 3.4e-802 |
| C26 | E score = 1.9e-089 | E score = 2.1e-049 | E score = 3.1e-034 | E score = 2.8e-020 | E score = 2.1e-016 |
| C27 | E score = 4.8e-449 | E score = 3.7e-217 | E score = 3.3e-214 | E score = 1.4e-144 | E score = 6.8e-085 |
| C28 | E score = 2.3e-183 | E score = 1.2e-037 | E score = 1.2e-073 | E score = 1.1e-025 | E score = 5.7e-025 |
| C29 | E score = 4.3e-330 | E score = 5.3e-127 | E score = 8.1e-102 | E score = 7.1e-101 | E score = 1.1e-080 |
| C30 | E score = 4.2e-305 | E score = 7.6e-086 | E score = 2.9e-074 | E score = 4.2e-082 | E score = 8.9e-041 |
| C31 | E score = 9.2e-422 | E score = 1.6e-240 | E score = 2.6e-194 | E score = 5.6e-177 | E score = 2.2e-098 |
| C32 | E score = 1.2e-344 | E score = 3.3e-103 | E score = 2.6e-066 | E score = 3.4e-061 | E score = 1.5e-050 |
| C33 | E score = 1.1e-364 | E score = 5.6e-135 | E score = 1.3e-129 | E score = 1.3e-090 | E score = 2.8e-075 |
| C34 | E score = 1.3e-682 | E score = 1.7e-494 | E score = 1.4e-438 | E score = 8.7e-395 | E score = 6.0e-336 |
| C35 | E score = 1.0e-481 | E score = 8.2e-222 | E score = 1.4e-195 | E score = 3.5e-190 | E score = 6.5e-190 |
| C36 | E score = 1.0e-515 | E score = 4.3e-429 | E score = 3.1e-339 | E score = 4.9e-299 | E score = 4.6e-237 |
| C37 | E score = 4.4e-593 | E score = 3.2e-552 | E score = 3.3e-267 | E score = 8.3e-229 | E score = 9.8e-180 |
| C38 | E score = 6.6e-1753 | E score = 3.8e-432 | E score = 1.7e-332 | E score = 2.9e-310 | E score = 5.8e-243 |
